# Electrostatic Potential Field Effects on Amine Macrocyclizations within Yoctoliter Spaces: Supramolecular Electron Withdrawing/Donating Groups

**DOI:** 10.1021/acs.jpcb.1c05238

**Published:** 2021-08-06

**Authors:** Wei Yao, Kaiyu Wang, Yahya A. Ismaiel, Ruiqing Wang, Xiaoyang Cai, Mary Teeler, Bruce C. Gibb

**Affiliations:** Department of Chemistry, Tulane University, New Orleans, Louisiana 70118, United States

## Abstract

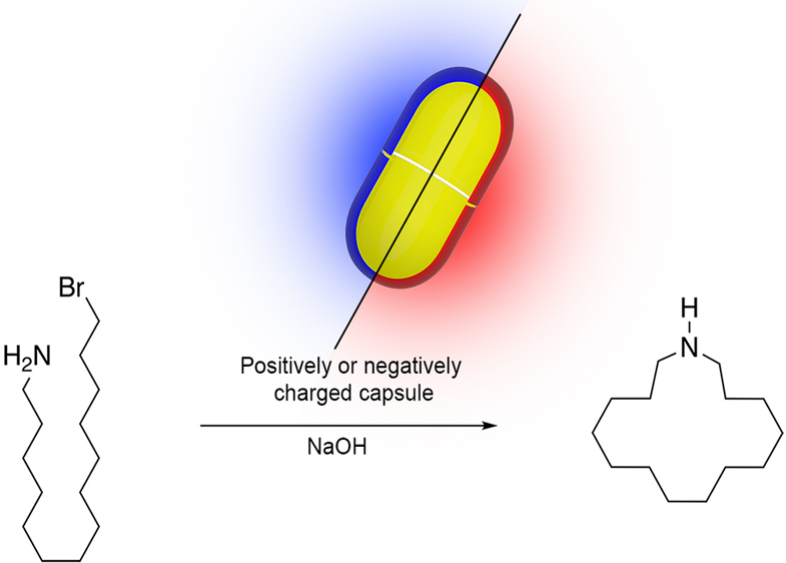

The central role of Coulombic interactions in enzyme catalysis has inspired multiple approaches to sculpting electrostatic potential fields (EPFs) for controlling chemical reactivity, including ion gradients in water microdroplets, the tips of STMs, and precisely engineered crystals. These are powerful tools because EPFs can affect all reactions, even those whose mechanisms do not involve formal charges. For some time now, supramolecular chemists have become increasingly proficient in using encapsulation to control stoichiometric and catalytic reactions. However, the field has not taken advantage of the broad range of nanocontainers available to systematically explore how EPFs can affect reactions within their inner-spaces. With that idea in mind, previously, we reported on how positively and negatively charged supramolecular capsules can modulate the acidity and reactivity of thiol guests bound within their inner, yoctoliter spaces (Cai, X.; Kataria, R.; Gibb, B. C. *J. Am. Chem. Soc*. **2020**, *142*, 8291–8298; Wang, K.; Cai, X.; Yao, W.; Tang, D.; Kataria, R.; Ashbaugh, H. S.; Byers, L. D.; Gibb, B. C. *J. Am. Chem. Soc.***2019**, *141*, 6740–6747). Building on this, we report here on the cyclization of 14-bromotetradecan-1-amine inside these yoctoliter containers. We examine the rate and activation thermodynamics of cyclization (Eyring analysis), both in the absence and presence of exogenous salts whose complementary ion can bind to the outside of the capsule and hence attenuate its EPF. We find the cyclization rates and activation thermodynamics in the two capsules to be similar, but that for either capsule attenuation of the EPF slows the reaction down considerably. We conclude the capsules behave in a manner akin to covalently attached electron donating/withdrawing groups in a substrate, with each capsule enforcing their own deviations from the idealized S_N_2 mechanism by moving electron density and charge in the activated complex and TS, and that the idealized S_N_2 mechanism inside the theoretical neutral host is relatively difficult because of the lack of solvation of the TS.

## Introduction

Inspired by the strength and long range of Coulombic interactions and their central role in the properties of enzymes,^1−6^ multiple approaches for controlling chemical reactions by sculpting electrostatic potential fields (EPFs) have been considered.^7^ Examples include utilizing the concentration gradients and the resulting EPF gradients in water microdroplets,^8^ using the intense EPFs of STM tips,^9−12^ or employing crystal engineering to sculpt EPFs within crystals.^13,14^ These represent powerful potential tools for chemists; even reactions whose mechanisms do not involve formal charges, e.g., the classic Diels–Alder reaction, can be accelerated by external EPFs because many formally covalent species along the reaction pathway are stabilized by minor charge-separated resonance contributors that are themselves affected by the applied field.^9,15^

One of the first examples of solution-based control of EPFs in reactions utilized diethyl ether solutions of 5 M LiClO_4_ and took advantage of the dissimilar coordination (supramolecular) properties of Li^+^ and ClO_4_^–^ ions.^16^ Since that time, although supramolecular chemistry has become increasingly proficient in using encapsulation to control stoichiometric and catalytic reactions,^17−36^ the field has not taken advantage of the broad range of nanocontainers available to systematically explore how EPFs can affect reactions within their inner-spaces.^37^

**Figure 1:**
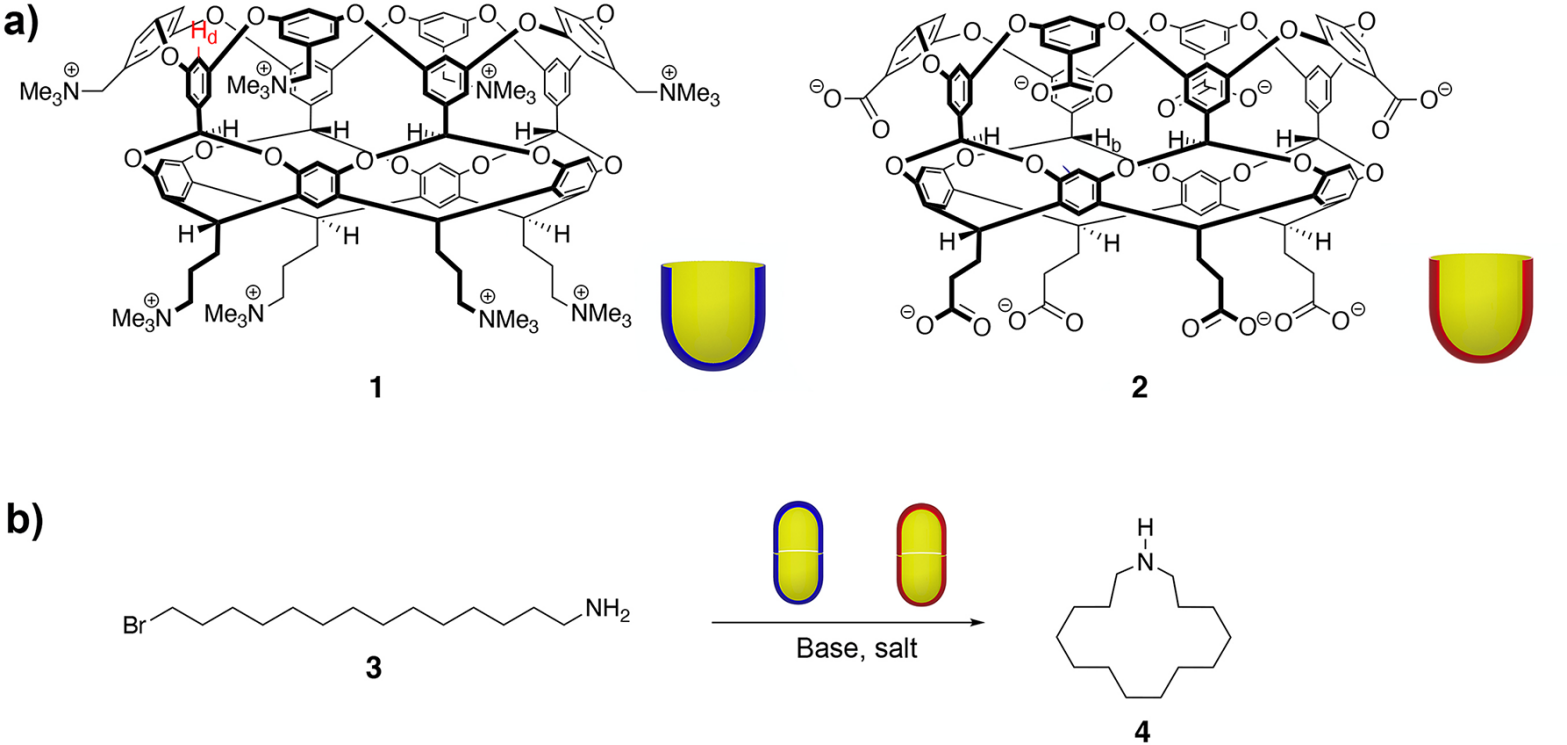
(a) Hosts utilized in this study: positand **1** and octa-acid (carboxylate) **2**. Also shown are schematic representations of the hosts as, respectively, blue and red bowls. (b) Cyclization of 14-bromotetradecan-1-amine **3** inside capsules **1**_2_ and **2**_2_ to give azacyclopentadecane **4**.

As a step toward understanding how the EPFs of hosts can affect the reactions of internalized guests, we recently reported on two, oppositely charged, yoctoliter containers built from positand **1** and octa-acid **2** (Figure 1).^38−40^ Through the hydrophobic effect, these cavitands dimerize around guests to form container complexes possessing identically shaped, low dielectric inner-spaces that differ in one key point; namely, the yoctoliter inner-spaces of **1**_2_ and **2**_2_ are, respectively, enveloped in a positive and negative EPF generated by the water-solubilizing charged groups of each cavitand. How does this difference affect chemical reactivity? We have shown that bound thiol guests are more acidic inside **1**_2_, by up to 2.5 p*K*_a_ units (it transpires that guest motif has a much larger effect on p*K*_a_).^41^ We have also observed that rates of macrocyclization of α,ω-thio-alkane halides inside **1**_2_ are almost 4 orders of magnitude faster than inside **2**_2_.^42^ These findings also showed that the stabilization of the transition states (TS) for cyclization was greater than that of thiolate stabilization, suggesting a rather complex mechanism by which these capsules affect chemical reactivity.

To extend our understanding of these yoctoliter reaction flasks further, we report here on the cyclization of 14-bromotetradecan-1-amine (**3**, Figure 1) inside yoctoliter containers **1**_2_ and **2**_2_. We examine the rate of formation of azacyclopentadecane (**4**), both in the presence of a minimum amount of exogenous salt, as well as in the presence of excesses of a range of different salts to examine how capsule counterion changes affect its EPF and hence the rate of cyclization within. We also perform Eyring analysis in the absence and presence of selected salts to determine the activation thermodynamics and how these are affected by exogenous salt binding. Overall, we find the cyclization rates and activation thermodynamics to be relatively insensitive to the nature of the EPF (**1**_2_ versus **2**_2_), but that for either capsule attenuation of the EPF slows the reaction down considerably. We conclude the capsules behave in a manner akin to electron donating/withdrawing groups covalently attached to the reaction center of **3**, with each capsule enforcing their own deviations from the idealized S_N_2 mechanism by moving electron density and charge in the activated complex and TS. Additionally, we conclude that the idealized S_N_2 mechanism inside the theoretical neutral host is relatively difficult because of the lack of solvation of the TS.

## Results and Discussion

### Host and Guest Synthesis, and Complex Formation

Cavitands **1** and **2** were formed using previously described procedures.^38−40^ Guest **3** was synthesized via a four-step process from the corresponding diacid: double reduction (BH_3_-Me_2_S/B(OMe)_3_) and bromination (NBS), followed by monoamination (phthalimide/K_2_CO_3_, then AcOH/HBr). Full details are given in the Supporting Information (SI, Section 2).

**Figure 2:**
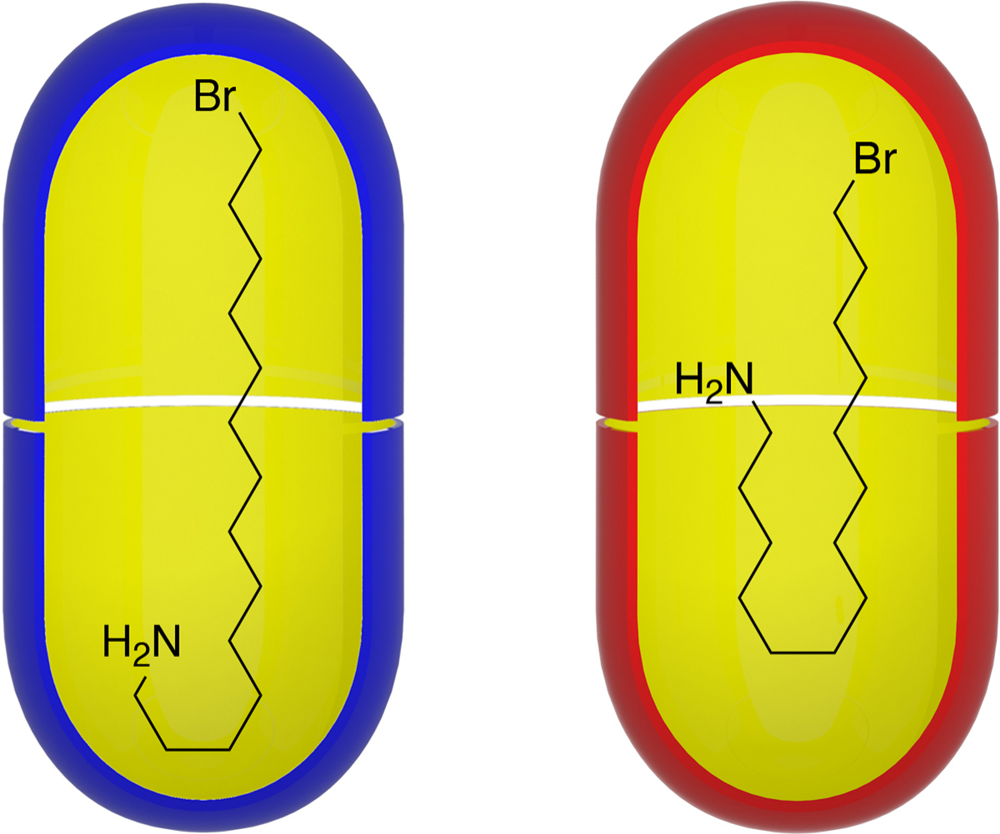
Representations of the average motif of guest 14-bromotetradecan-1-amine (**3**) inside capsules **1**_2_ and **2**_2_.

Solutions of **1** and **2** were formed by dissolving the material in D_2_O containing a small excess of NaOD. The capsular complexes with the two hosts were then formed by the addition of a slight excess of half an equivalent of guest **3**·HBr in D_2_O (SI Section 3). Figures S5–S12 show the ^1^H NMR, DOSY NMR, and COSY NMR spectra of the two complexes. The ^1^H NMR signals of guest **3** in the free (in D_2_O) and encapsulated states were used to calculate the Δδ values for each signal. It is well-established that, because of the *D*_4*h*_ point group of the inner-space (approximately a spheroid or ellipsoid of revolution), the Δδ value of a group is reflective of its average location within the inner-space; as a rule of thumb, the closer a group is to a pole, the more its signal is upfield shifted because of its inevitable proximity to one or more aromatic rings.^43^ Hence, with 16 atoms in the chain, we anticipated that a Δδ value analysis would reveal that in both capsules the guest adopt a U- or J-motif.^21,44−49^ This was indeed the case, but despite the two inner-spaces being identical in shape, the guest adopted a slightly different motif within the two hosts (Figure 2, and SI Figures S8 and S12). The Δδ values for guest **3** within **1**_2_ suggest an average motif in which four contiguous carbons at the amine terminus are all located deep in one polar region, the section of the mainchain around C-9 is located at the equator, and the halide terminus is deep within the opposing polar region of the capsule. In contrast, within **2**_2_ the guest adopts a more distinct J-shaped motif in which the C-6/C-7 region of the guest is a turn located in one polar region of the capsule, the halide terminus occupies the other, and the amino group/ammonium is located at the equator.

Three principal factors likely contribute to the subtle difference in average conformation of the guest in the two capsules. First, it may be the case that the hemispheres of capsule **1**_2_ are slightly further apart than in **2**_2_; the solubilizing groups of the former are more weakly solvated than the carboxylates of the latter,^50^ and hence, the Coulombic repulsion between the hemispheres of **1**_2_ is likely higher. This would lead to a slightly greater inner-space volume in **1**_2_. Second, the different motifs may arise from the different ion–dipole interactions between the positively charged solubilizing groups of **1**_2_ or the negatively charged groups of **2**_2_, and the two terminal dipoles (CH_2_→Br and CH_2_→NH_2_) of the guest. Prior modeling suggests a reasonably homogeneous EPF within each yoctoliter space,^42^ but it may be the case that the intrinsic asymmetry of the different ion–dipole interactions, combined with small gradients within each, alters the preferred motif of the guest. Finally, differences in the p*K*_a_ of the bound guest may be the cause of the motif changes. Although it was not possible to observe the ^1^H NMR signal for a −NH_2_ or −NH_3_^+^ group of the bound guest, and therefore not possible to use ^1^H NMR spectroscopy to determine ammonium p*K*_a_ values, we can use the previously determined p*K*_a_ values for thiols within **1**_2_, and **2**_2_ as a guide.^41^ This data suggests that the p*K*_a_ of a bound ammonium can range from 6 to 12; the amine would be a weak base inside **1**_2_, and a relatively strong base inside **2**_2_. That trace protonation of the guest inside **2**_2_ is a possibility that was suggested by a small change in the guest binding region of the ^1^H NMR spectrum of the complex when an additional 50 equiv of NaOD was added. However, this change did not itself discernably change the overall guest binding motif. In short, our evidence to date suggests that changes in p*K*_a_ may play somewhat of a role in the differences in guest motif in the two capsules, but that other possibilities (capsule volume differences or changes in ion-dipole interactions between capsule and guest) cannot be excluded.

### Cyclization of Guest **3** in the Absence of Excess Salt

Cavitands are now well-established agents for inducing cyclization reactions,^21,42,51−53^ and heating of either complex led to a quantitative yield of azacyclopentadecane **4**. There was no evidence of side reactions such as elimination or hydrolysis, nor polymer formation; this latter point confirms that cyclization occurred exclusively within the inner-spaces of the capsules. To gather information about these cyclization reactions, we performed Eyring analyses using ^1^H NMR spectroscopy (SI, Section 4).^54^ In these experiments, the H_d_ proton of each cavitand (Figure 1, highlighted in red in **1**) proved to be a useful reporter to monitor product formation. Thus, we determined the cyclization rate constants at five different temperatures for each complex. In the case of the cyclization of **3** within **1**_2_, the reaction was monitored between 339 and 351 K, while in the case of the reaction inside **2**_2_ the temperature range was 325–338 K. The obtained thermodynamic data shown in Table 1 reveals that the *ΔH*^⧧^ for cyclization is considerably lower within **1**_2_ than within **2**_2_ (ΔΔ*H*^‡^ = 14.7 kJ mol^–1^). Interestingly, this difference is smaller than that seen in the cyclization of thiolates within the two capsules (ΔΔ*H*^⧧^ = 24.3 kJ mol^–1^), suggesting that the difference in charge development in the TSs for the formation of **4** inside **1**_2_ versus inside **2**_2_ is quite small. Although the reaction within **1**_2_ possesses a lower enthalpy barrier, cyclization is more entropically penalized. One possible cause for this difference in −*T*Δ*S*^⧧^ is the location of the two termini of the guest in each complex (Figure 2); the greater average distance between the termini of the guest in the **1**_2_ capsule would result in a higher entropic penalty to reach the TS. Because of these noted enthalpy and entropy differences, in the two capsules the reaction free energies of activation, rate constant, and half-life are not significantly different.

**Table 1:** Eyring Data for the Cyclization of Guest **3** within Capsules **1**_2_ and **2**_2_^a^

	**3** in **1**_2_	**3** in **2**_2_
*k* (s^–1^, 338 K)	3.47 × 10^–5^	6.25 × 10^–5^
*k* (s^–1^, 298.15 K)	1.77 × 10^–6^	1.58 × 10^–6^
half-life (s, 338 K)^a^	2.00 × 10^4^	1.10 × 10^4^
half-life (s, 298.15 K)^a^	3.90 × 10^5^	4.38 × 10^5^
Δ*G*^⧧^ (kJ mol^–1^)	105.8	106.2
Δ*H*^⧧^ (kJ mol^–1^)	59.8	74.5
–*T*Δ*S*^⧧^ (kJ mol^–1^)	45.9	31.7

a Errors in individual rate constant determinations <10%. Error in Eyring analysis (Δ*G*^⧧^, −Δ*H*^⧧^, *T*Δ*S*^⧧^) 5%.

**Figure 3:**
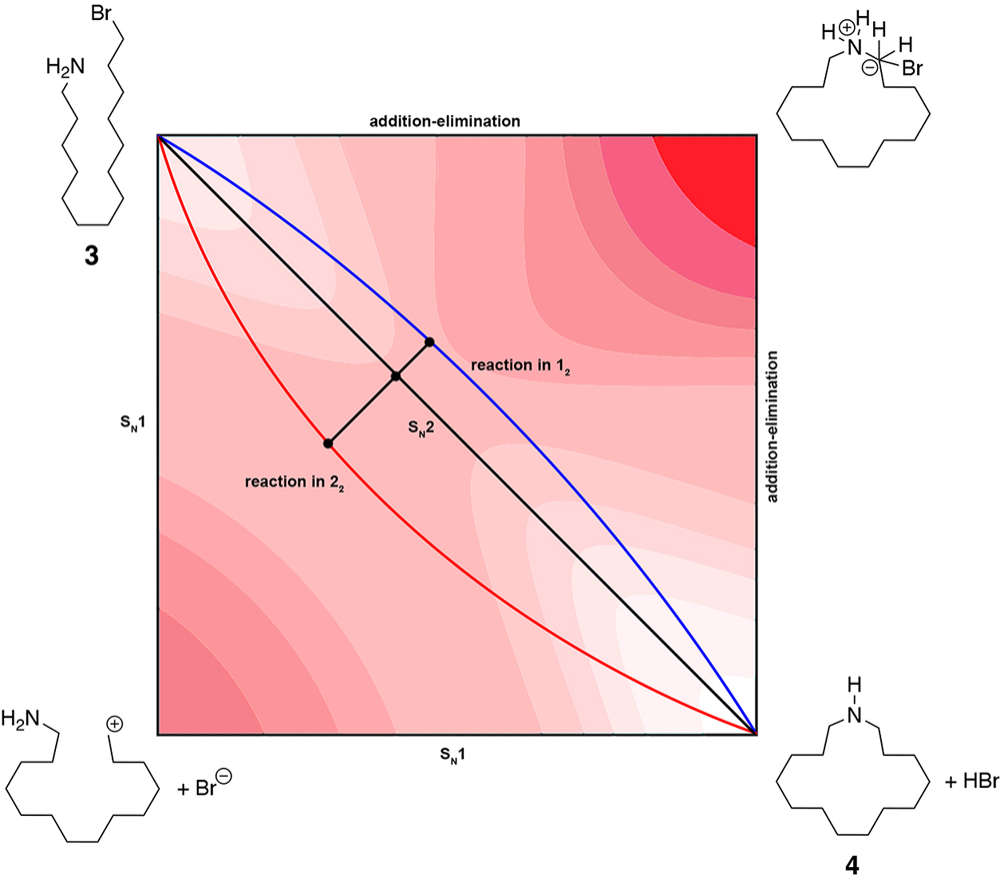
Representative More O’Ferrall–Jencks diagram illustrating the effects upon the cyclization of 14-bromotetradecan-1-amine (**3**) within the inner-spaces of positively and negatively charged capsules **1**_2_ and **2**_2_. The black diagonal from top left to bottom right corresponds to the reaction coordinate of the idealized S_N_2 mechanism. The left and lower axes correspond to the reaction coordinate of the idealized S_N_1 mechanism, and top and right axes correspond to the reaction coordinate of an addition–elimination process involving a pentavalent carbanionic TS. The blue and red curves correspond to the enforced mechanisms within the inner-spaces of positively and negatively charged capsules **1**_2_ and **2**_2_.

As we have not observed encapsulation to shift p*K*_a_ values of ionizable guests by more than 6 units, mechanistically we envision the S_N_2 cyclization of **3** to involve the free amine; there is no amide ion formation since in the most favorable situation (inside positively charged **1**_2_) the p*K*_a_ of an amine is unlikely to be below 30–32. Assuming an amine nucleophile, how do the capsules affect the mechanism? One way to envisage the effects of the EPF of the two capsules is by a More O’Ferrall–Jencks diagram (Figure 3), i.e., a plan projection of a reaction energy surface.^55,56^ In the following discussion, we assume that the stabilization of the (partially) charged carbon center is energetically key, because its relatively high instability is disproportionally affected by the EPF of the capsule. In Figure 3, the black diagonal from top left to bottom right corresponds to the reaction coordinate of the idealized S_N_2 mechanism from **3** to **4**, the left and lower axes correspond to the reaction coordinate of the idealized S_N_1 mechanism, and the top and right axes correspond to the reaction coordinate of an addition–elimination process involving a pentavalent carbanionic TS.^57,58^ The transition state for the S_N_2 mechanism (•) occurs relatively early in the reaction coordinate because of the overall exergonic nature of the cyclization (Hammond postulate). When the reaction is carried out in the negative EPF of capsule **2**_2_, the reaction coordinate (red) is pulled toward the lower left as the field of the host enforces a mechanism involving the development of a greater degree of carbocation character in the TS. Compared to the idealized S_N_2 mechanism, the activated complex and TS involve relatively long N···C and C···Br distances. In other words, capsule **2**_2_ behaves as the supramolecular equivalent of a typical (covalently attached) electron donating group (EDG), with the EPF influencing electron distributions in the TS in a manner similar to the orbital effects arising from electronegativity differences between covalently attached EDGs. In contrast, within the positive EPF of capsule **1**_2_, the reaction coordinate (blue) is curved toward the upper right as the field of the host enforces the development of a greater degree of carbanion character in the TS with relatively short N···C and C···Br distances. Thus, capsule **1**_2_ behaves as the supramolecular equivalent of an electron withdrawing group (EWG) on the reaction center. We show an asymmetry in the degree of deviation from the idealized S_N_2 coordinate for the positive and negative capsules to reflect the idea that a trigonal bipyramidal (*D*_3*h*_), hypervalent carbon represents a particularly high energy TS.^57−59^

To gain further insight into the cyclization of **3**, we opted to probe how the addition of excess (exogenous) salts affected the rate and activation thermodynamics of the reaction. As we discuss, studies of the effects of salts not only provide more insight into the reaction mechanism but also reveal the differences in solvation of the two capsules and how this affects reactions in the inner-space, as well as some of the limitations of these hosts as yoctoliter reaction flasks.

### The Effects of Exogenous Salts on Cyclization

The effective charge of the two host capsules is less than their formal ±16 because of attenuation from associating counterions, and charge transfer to the solvation shell waters. Regarding ion association, we have previously demonstrated both specific ion binding to the crown of four ammonium groups defined by the pendent groups in **1**,^60^ as well as nonspecific association (ion condensation) to the host and its dimer capsule.^41,61^ In contrast, there are no specific cation binding sites in **2** or its capsule; only weak ion condensation has been observed.^41,62^ Irrespective of the type of binding, increases in the ionic strength and/or swapping the capsule counterions by the addition of ions with higher cavitand affinity lead to a reduction in its EPF. In the case of the capsules, this can induce a change in the nature of an assembly^62^ or modulate the chemical reactivity of an internalized guest.^42^

To gain more insight into the mechanism of the cyclization of **3** within the capsules, we investigated the effects on the nature of exogenous salts upon reaction rates within the two hosts (SI, Section 5). To minimize pairing between the ions of the added salt, we selected highly solvated sodium and chloride counterions for, respectively, studying how anions and cations affect cyclization rates within **1**_2_ and within **2**_2_.^61,62^ In all cases, the rate constants for cyclization were measured in the presence of 10 mM added salt and compared to the rate of the reaction with only base (NaOD) present. In most cases, ^1^H NMR spectroscopy revealed that the added salt had no direct effect on the bound guest. However, in the presence of anions that bind strongly to the pocket of host **2**, namely, TfO^–^, ReO_4_^–^, ClO_4_^–^, and SCN^–^,^60^ the bound guest region of the ^1^H NMR spectrum did undergo small changes indicative of an alteration of the guest ingression–egression kinetics, and/or partial guest displacement by anion competition (see below).

**Figure 4:**
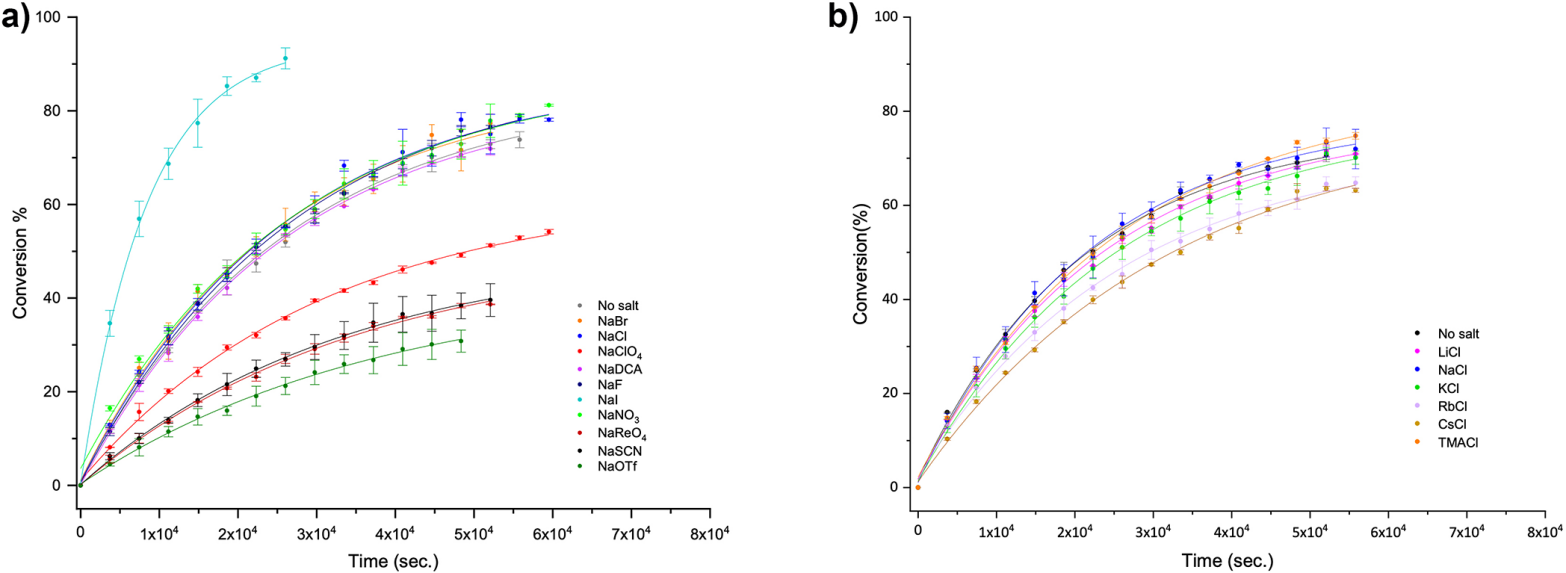
Plots of the conversion percentage as a function of time and salt for the formation of **4**. In both cases (a, b), the host-**3** complex concentration was 1 mM, and the total NaOD and/or salt concentration was 10 mM. For the effects of anions on the formation of **4** inside **1**_2_ (a), the reaction was carried out at 339 ± 1 K. For the effects of cations on the formation of **4** inside **2**_2_ (b), the cyclization temperature was 336 ± 1 K.

Figure 4a shows how anion association to the outside of **1**_2_ affects the formation of **4**, while Table 2 presents the corresponding rate constant data. Iodide is the outlier here, accelerating the reaction considerably; all other anions either have little effect or reduced the rate constant. Iodide is known to enter the inner-space via capsule breathing, the microsecond time scale, the partial opening of the complex that allows the entry, and the exit of small solutes without allowing principal guest egression.^35,63,64^ Thus, we believe that in the presence of NaI there are two sequential reactions occurring within **1**_2_: halogen exchange to form the corresponding iodinated guest, and then cyclization. Iodide is known to associate with the outside of **1** and its capsule,^60^ so a rate attenuation mechanism (see below) is also likely to be in operation. However, this is evidently small compared to the increased cyclization rate arising from the *in situ* iodination.

**Table 2:** Reaction Rate Constant (*k*) for the Cyclization of **3** in **1**_2_ or **2**_2_ in the Presence of Different Salts^a^

Na^**+**^ salt	rate constant,^b^*k* in 10^–5^ s^–1^	Cl^–^ salt	rate constant,^c^*k* in 10^–5^ s^–1^
I^–^	12.00	no salt	4.81
Br^–^	4.07	Na^+^	4.61
Cl^–^	3.91	Li^+^	4.26
CHCl_2_CO_2_^–^	3.83	K^+^	3.97
no salt	3.80	N(Me_4_)^+^	3.92
NO_3_^–^	3.76	Rb^+^	3.88
F^–^	3.69	Cs^+^	3.15
ClO_4_^–^	3.25		
SCN^–^	2.98		
ReO_4_^–^	2.86		
TfO^–^	2.62		

a Errors in individual rate constant determinations <10%.

b At 339 ± 1 K.

c At 336 ± 1 K.

The other anions either had little effect on the rate constant or caused its reduction. Reaction rate constants in the presence of strongly solvated F^–^, Cl^–^, Br^–^, and NO_3_^–^, and amphiphilic CHCl_2_CO_2_^–^, were very similar to salt free conditions. These have been shown to be the weakest of binders to the outside of **1**,^60^ so this finding is not unexpected. In contrast, stronger binding, charge diffusion and weakly solvated SCN^–^, ClO_4_^–^, ReO_4_^–^, and TfO^–^ all attenuated cyclization rates. In these cases, extrapolation of the data indicated low yields of **4** in the region of 50–70%, suggesting that anion competition for the inner-space of the capsule (see above) leads to leakage of guest **3** and its ultimate polymerization or hydrolysis in free solution.

Figure 4b shows the raw conversion data as a function of time and cation for the cyclization of **3** within **2**_2_, while Table 2 summarizes the obtained rate constants. In general, the effect of alkali metal cations on the cyclization of **3** within **2**_2_ was found to be considerably smaller than the anion effect with **1**_2_ (Figure 4). This result is consistent with the high solvation free energy of carboxylate groups; capsule **2**_2_ cannot pair strongly with its counterions.

As with the anion effect on **1**_2_, all cations were observed to reduce the rate of cyclization inside **2**_2_. Although the effects of Na^+^ or Li^+^ were not significant, and in many cases differences between two salts were similar, a distinct trend was observed in which the larger and more polarizable cations attenuated cyclization more. Thus, the ability to attenuate the reaction was as follows: Cs^+^ > Rb^+^ > K^+^ > Li^+^ ≈ Na^+^ ≈ no salt. This trend was very similar to the ability of cations to reduce the net charge of the host and switch a dimeric capsule into a tetrameric cavitand assembly.^62^ Thus, contrary to Collins’ law of matching water affinities which predicts sodium ions pairing most strongly with carboxylates,^65^ by both the metrics of assembly switching and reaction attenuation, it is the larger, more weakly solvated cations that pair most strongly with the capsule. That noted, in addition to Coulombic interactions, cation−π interactions and other such ion–pole interactions may also be involved in the weak association of the cations with the outside of the cavity.

Figure 4 and Table 2 reveal that the attenuation of the EPF of either capsule increases the Δ*G*^⧧^ for cyclization of **3**. In other words, irrespective of whether a capsule enforces a mechanism that involves a TS with some degree or carbocation or carbanion character relative to the idealized S_N_2 process, attenuation of the EPF slows the reaction. Why is this? Our working hypothesis is that in each capsule, the energetic cost of charge development in the activated complex and TS is attenuated by guest realignment to maximize favorable interactions between the guest and capsule. As a result, the corresponding cyclization within the confines of a capsule with little or no EPF is more energetically demanding. In other words, in free solution, solvent can transfer charge and/or hydrogen bond with the attacking N atom or the departing Br^–^ group, but in a capsule devoid of any EPF and any groups that function as a formal proton shuttle or halide transporter, the S_N_2 process is energetically demanding. This hypothesis dovetails well with the general observation that S_N_2 processes involving charged species are slower in solvents of lower polarity.

For further information, we selected two of the aforementioned salts and carried out Eyring analyses to determine how ion association to the outside of a capsule affected the thermodynamics of TS formation. Specifically, we selected NaClO_4_ to probe the effects of ClO_4_^–^, on the cyclization of **3** within **1**_2_, and CsCl to examine how Cs^+^ condensation to **2**_2_ affected the reaction. Table 3 summarizes our findings. Briefly, attenuation of the EPF of both capsules leads to a rise in the enthalpy of activation. This rise is much larger in **1**_2_ (ΔΔ*H*^⧧^ = 23.8 kJ mol^–1^ versus 5.9 kJ mol^–1^ in **2**_2_) indicating that increased counterion affinity to **1**_2_ leads to a greater EPF attenuation in this capsule. It was not possible to add more NaClO_4_ without salting out of the complex. This, combined with the Δ*H*^⧧^ values for the reaction in both capsules in the presence of their respective salts being in the region of 80–90 kJ mol^–1^, suggests a value in this region for reaction in the theoretical neutral host. Interestingly, the −*T*Δ*S*^⧧^ values in the two EPF-attenuated capsules are also very similar, leading to similar overall Δ*G*^⧧^ values. However, the bound guest ^1^H NMR signal broadening and shifting in the presence of NaClO_4_ means that it is not possible to determine if the two complexes have similar guest motifs when the charges on the host are largely removed.

**Table 3:** Eyring Data for the Cyclization of Guest **3** within Capsules **1**_2_ and **2**_2_ in the Presence of 10 mM Added Salt

	**3** in **1**_2_ + NaClO_4_	**3** in **2**_2_ + CsCl
*k* (s^–1^, 338 K)	3.11 × 10^–5^	3.83 × 10^–5^
*k* (s^–1^, 298.15 K)	5.15 × 10^–7^	7.40 × 10^–7^
half-life (s, 338 K)^a^	2.20 × 10^4^	1.80 × 10^4^
half-life (s, 298.15 K)^a^	1.34 × 10^6^	9.36 × 10^5^
Δ*G*^⧧^ (kJ mol^–1^)	108.9	107.6
Δ*H*^⧧^ (kJ mol^–1^)	83.6	80.4
–*T*Δ*S*^⧧^ (kJ mol^–1^)	25.3	27.3

a Errors in individual rate constant determinations <10%. Error in Eyring analysis (Δ*G*^⧧^, −Δ*H*^⧧^, *T*Δ*S*^⧧^) 5%.

## Conclusions

Our results described here continue our investigation into the ability of capsules driven by the hydrophobic effect to function as yoctoliter reaction vessels. Our data reveals that both hosts can lead to essentially quantitative formation of cyclic amine **4**, and that relative to a theoretical charge neutral host, the reactions are promoted by both the positive EPF and the negative EPF of **1**_2_ and **2**_2_. We interpret this finding in terms of the EPF of each host being capable of stabilizing charge buildup in the activated complex and TS, but that each does so by enforcing different mechanisms from the idealized S_N_2 process. Moreover, we conclude that the S_N_2 mechanism in the theoretical neutral host devoid of an EPF is energetically demanding because of a lack of solvation in the inner-space of the capsule.

Our data also reveals that there is more control of the EPF in capsule **1**_2_ because it is more poorly hydrated and water-solubilizing, and charge groups pair more strongly with counterions. However, there is a limit here. Relatively high affinity, charge-diffuse anions can compete with **3** for the inner-space of the capsule and, as a result, attenuate the yield of product **4**. Conversely, maximal EPFs can be generated in solutions of low ionic strength using strongly solvated counterions that pair minimally with the capsule. We are continuing to study these assemblies as yoctoliter reaction flasks and will report our findings in due course.

## Supporting Information Available

Details of the synthesis of guest **3**, the guest encapsulation protocols, and a summary of the kinetic experiments and data analysis (PDF)
