# Supplementary material for: Electrostatic Potential Field Effects on Amine Macrocyclizations within Yoctoliter Spaces: Supramolecular Electron Withdrawing/Donating Groups
Source: J Phys Chem B. 2021 Aug 6;125(32):9333–40. doi: 10.1021/acs.jpcb.1c05238 (PMC8383300; doi:10.1021/acs.jpcb.1c05238)
Supplement: Supplementary file 1 — jp1c05238_si_001.pdf [file jp1c05238_si_001.pdf]

## Supporting Information

# Electrostatic potential field effects on amine macrocyclizations in yocto-liter spaces: Supramolecular electron withdrawing/donating groups

Wei Yao, Kaiyu Wang, Yahya A. Ismaiel, Ruiqing Wang, Xiaoyang Cai, Mary Teeler, and Bruce C. Gibb*

Wei Yao https://orcid.org/0000-0002-4229-0486

Xiaoyang Cai https://orcid.org/0000-0003-4806-1868

Bruce C. Gibb https://orcid.org/0000-0002-4478-4084

Department of Chemistry, Tulane University, New Orleans, LA 70118, USA

*Corresponding Author bgibb@tulane.edu

## Table of Contents


1) Materials and Instrumentation S3

2) Synthesis of guest **3** S4

3) Encapsulation of **3** within hosts $\mathbf{1}_2$ and $\mathbf{2}_2$ S7

4) Eyring analysis for the cyclization of **3** within $\mathbf{1}_2$ and $\mathbf{2}_2$ S12

   4.1) Reaction of **3** within $\mathbf{1}_2$ at different temperatures and data fitting S13

   4.2) Reaction of **3** within $\mathbf{2}_2$ at different temperatures and data fitting S20

5) Cyclization of **3** within $\mathbf{1}_2$ in the presence of various salts S27

   5.1) Anion effect: Summary of data S28

   5.2) Anion effect: Individual data S31

6) Cyclization of **3** within $\mathbf{2}_2$ in the presence of various salts S42

   6.1 Cation effect: Summary of data S43

   6.2 Cation effect: Individual data S45

7) Eyring analysis for the cyclization of **3** presence of 10 mM $NaClO_4$ or CsCl S52

8) References S65

## 1) Materials and Instrumentation

Deep-cavity cavitand **1** (Positand-1) and **2** (octa-acid) were synthesized following the previously reported procedures.[1-3] All NMR spectra were recorded on a Bruker 500 MHz spectrometer. All spectra were processed with MestReNova (Mestrelab Research). All reagents were purchased from Aldrich Chemical Company and, with the exception of *N*-bromosuccinamide (recrystallized from boiling water), used without further purification. Guest **3**, 14-bromotetradecan-1-amine was synthesized and prepared in the form of its ammonium bromide salt. All reactions were performed under a nitrogen atmosphere.

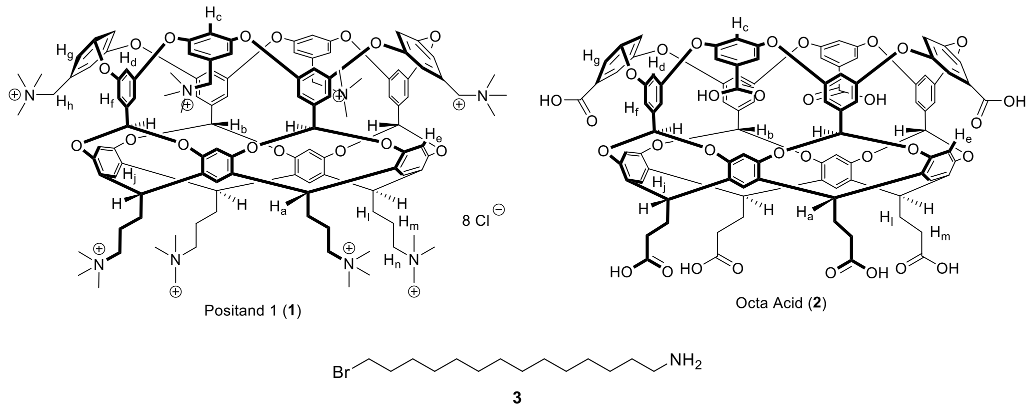


**Figure S1:** Chemical structures of host **1, 2** and guest **3**.

## 2) Synthesis of guest 3

The synthetic route of guest **3** is shown in **Scheme S1**. The synthesis started from the corresponding commercially available dodecanedioic acid.

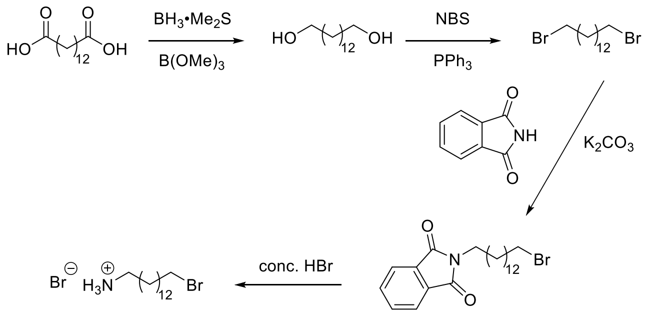


**Scheme S1**: Synthetic route for guest **3**.

### *Synthesis of 1,14-tetradecanediol*

The reducing agent $BH_3$-$Me_2S$ (3.6 mL, 36.87 mmol) and $B(OMe)_3$ (8 mL, 72 mmol) were dissolved in 40 mL anhydrous tetrahydrofuran (THF) at 0°C. Subsequently, a solution of tetradecanedioic acid (4.0 g, 15.50 mmol) in 80 mL THF was added over a 30 min. period. After this addition, the reaction mixture was heated to reflux for 24 hours. At the end of the reaction the flask and its contents were cooled to rt and quenched by the slow addition of 50 mL methanol. The crude solution was then stirred for 30 minutes. The combined solvent was then removed under reduced pressure to give an off-white powder as crude diol product (3.34 g, yield 94%). The $^1$H NMR spectrum matched that previously reported,[4] and the crude product used without further purification.

### *Synthesis of 1,14-dibromotetradecane*

Solutions of triphenylphosphine (3.14 g, 12 mmol) and freshly recrystallized *N*-bromosuccinimide (NBS, 2.14 g, 12 mmol) were made by dissolving each in 50 mL anhydrous THF at 0°C. Subsequently, the triphenylphosphine solution was added slowly into the vigorously stirred NBS solution. After this addition, a solution of 1,14-tetradecanediol (0.8 g, 3.47 mmol) in 25 mL THF was slowly added to this mixture. The reaction mixture was subsequently heated at 50°C for 4 h. Upon cooling to rt, the solvent was removed under reduced pressure to give the crude product as an off-white powder. This was then recrystallized in ethanol to obtain 1.1 g of the pure product as a white powder (0.9 g, 74% yield). The $^1$H NMR spectrum of the product matched that previously reported.[5] The product used without further purification.

### *Synthesis of N-(14-bromotetradecyl) phthalimide*

1,14-dibromotetradecane (1 g, 2.8 mmol) was dissolved in 50 mL of acetone, before phthalimide (520 mg, 2.8 mmol) was added to the stirring solution. Once full dissolution had occurred, potassium carbonate powder (1.38 g, 10 mmol) was also added, and the reaction

mixture heated to 60°C overnight. After cooling to rt, the solvent was removed under reduced pressure and the product purified by normal-phase silica-gel column chromatography (mobile phase: 100% hexane to 80% hexane/20% acetone) and ($R_f$ = 0.6). The fractions containing the product were then combined, and the solvent removed under reduced pressure. Drying under high vacuum overnight yielded the product as a white solid (720 mg, yield 31%). The $^{1}$H NMR spectrum of the product matched that previously reported.[6]

### *Synthesis of 14-bromotetradecane-1-amine 3*

*N*-(14-bromotetradecyl) phthalimide (700 mg, 166 mmol) was added to 20 mL of glacial acetic acid to form a suspension, before 10 mL of concentration hydrobromic acid was subsequently added. The mixture was then heated at 100°C for 48 h. During the first 2 h, the solution became a clear, light orange solution. After 48 h the reaction was cooled to rt and the solvent removed under reduced pressure until dryness to give an orange ppt. Subsequently, 30 mL of acetone was added to the crude product and the mixture sonicated for 1 min. The resulting suspension was then filtered to give the pure bromide salt as a white powder (418 mg, 65%). The $^{1}$H-, $^{13}$C-NMR and HRMS spectra are shown in **Figures S2-S4**. $^{1}$H NMR (500 MHz, $d_6$-DMSO, ppm) δ 7.62 (s, 1H), 3.53 (t, *J* = 6.7 Hz, 2H), 2.91 – 2.60 (m, 2H), 1.79 (dq, *J* = 8.6, 6.8 Hz, 2H), 1.52 (t, *J* = 7.4 Hz, 2H), 1.37 (q, *J* = 6.9 Hz, 2H), 1.26 (m, 18H). $^{13}$C-NMR (126 MHz, $d_6$-DMSO, ppm) δ 35.72, 32.69, 29.47, 29.38, 28.97, 28.56, 27.96, 27.44, 26.23. HRMS, Calculated for $C_{14}H_{30}BrN$: m/z, $[M+H]^+$, 292.1634; Found: 292.1628.

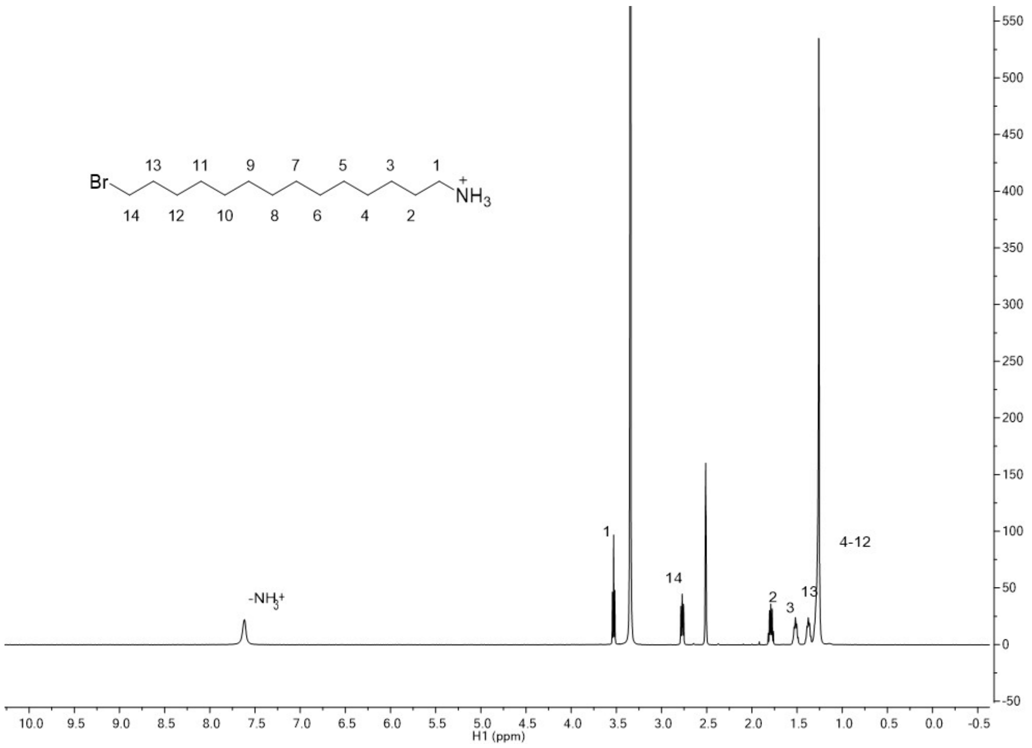


**Figure S2:** $^{1}$H NMR spectrum of 14-bromo-1-tetradecanethiol **3** in $d_6$-DMSO (500 MHz).

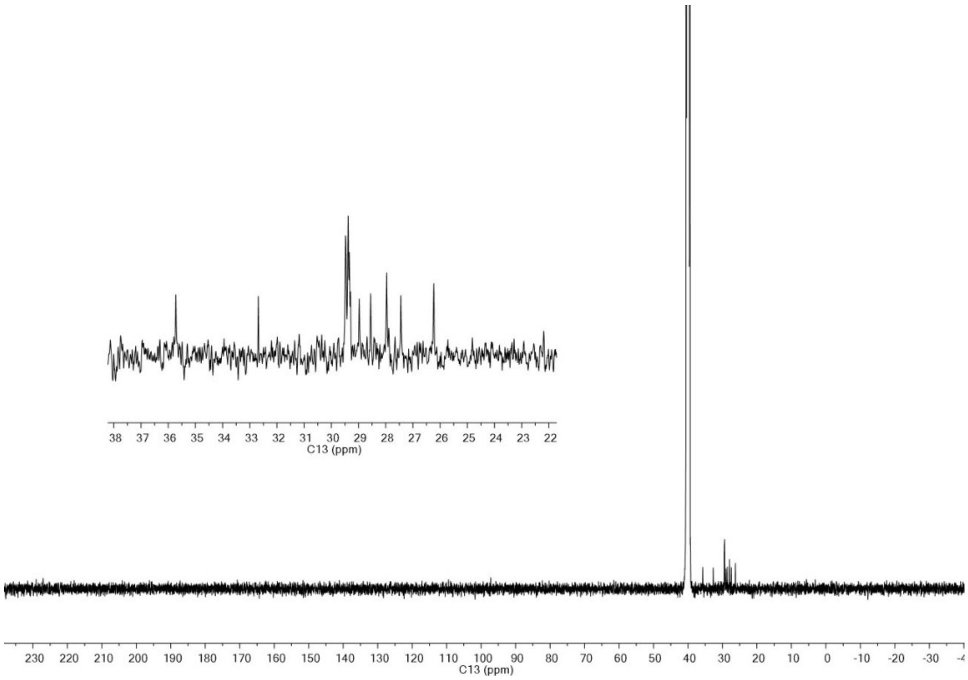


**Figure S3:** $^{13}C$ NMR spectrum of 14-bromo-1-tetradecanethiol **3** in $d_6$-DMSO (126 MHz). Inset shows key signals in more detail.

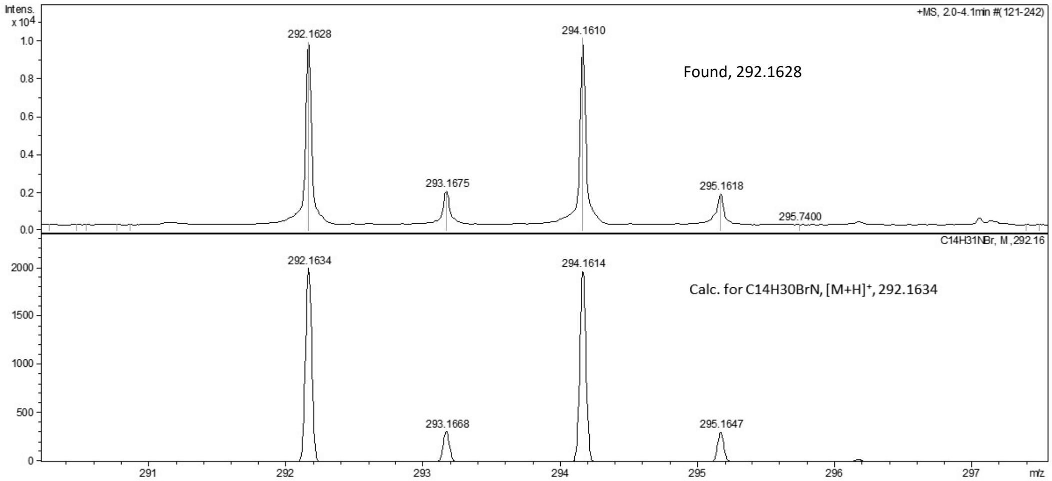


**Figure S4:** HRMS (ESI) of 14-bromo-1-tetradecanethiol **3**.

**3) Encapsulation of 3 within hosts $\mathbf{1_2}$ and $\mathbf{2_2}$**

1 mM $D_2O$ solutions of hosts **1** and **2** were prepared in the presence of 10 equivalents of NaOD. Subsequently, the bromide salt of guest **3** (14-bromotetradecan-1-ammonium bromide, **3**·Br) was added and the complex formed by vortexing for 10 minutes at 25 ˚C. **Figure S5-S12** show the $^1$H NMR, DOSY NMR, and COSY NMR spectra, as well as the calculated Δδ values for each set of protons of the guest, for both the complex with host **1** and **2**. This data confirmed that both complexes possessed a 2:1 stoichiometry.

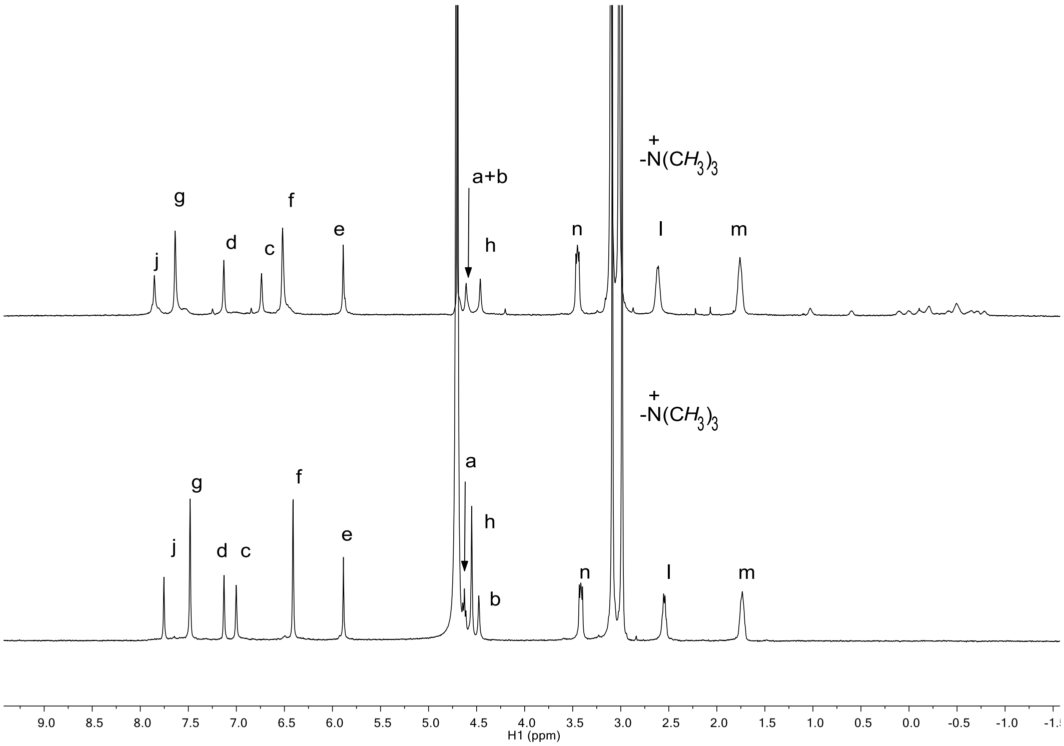


**Figure S5:** Stack $^{1}$H NMR of free host **1**(bottom) and the host-guest complex **3@1₂**(top).

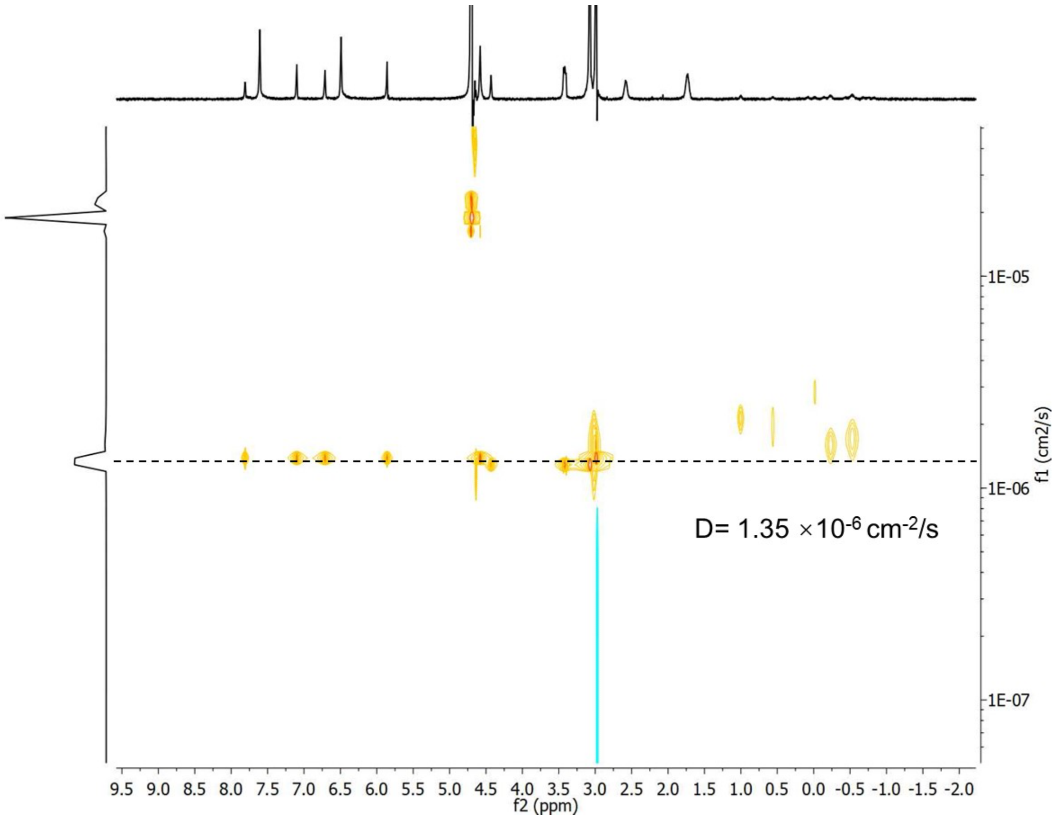


**Figure S6:** DOSY NMR spectrum of complex **3@1₂**.

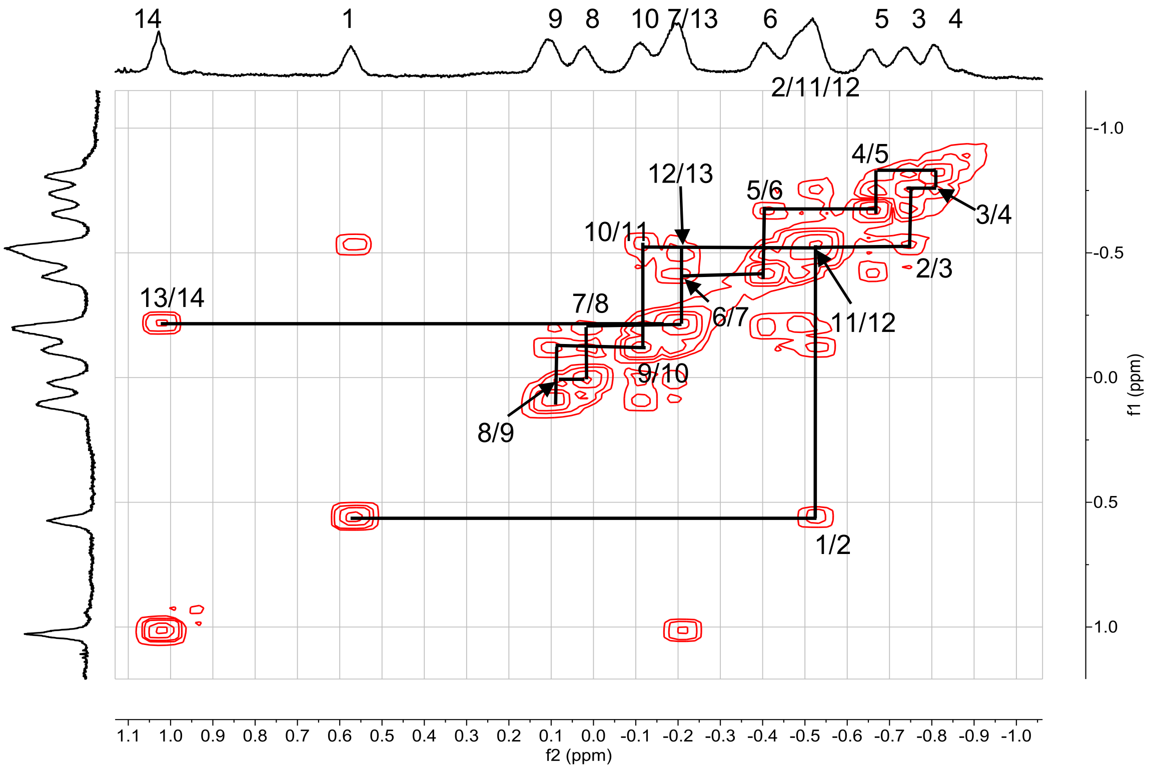


**Figure S7:** $^1$H-$^1$H COSY NMR spectrum of the bound guest region and peak assignment of complex **3@1**$_2$.

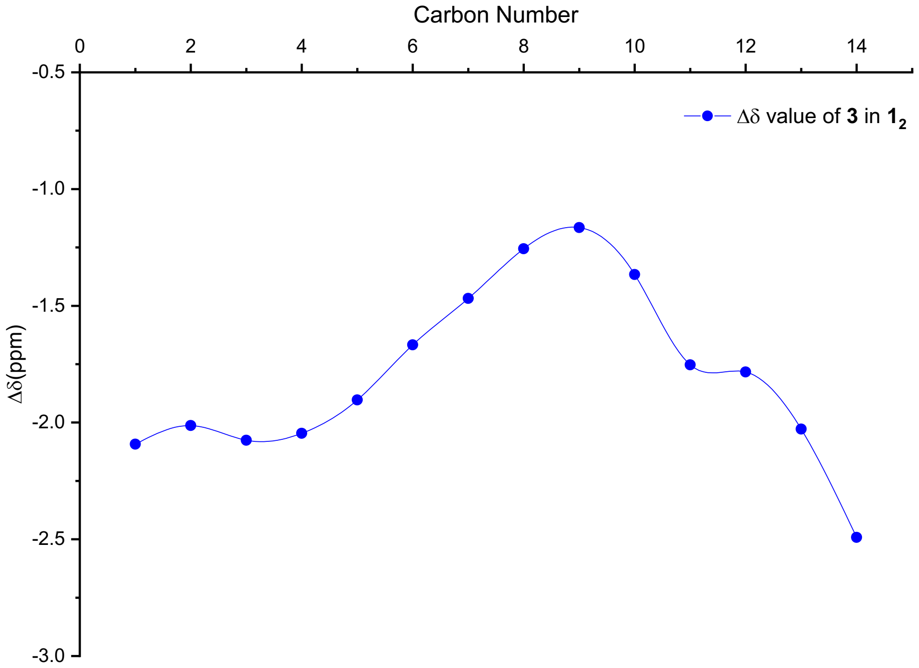


**Figure S8** Plot of $^1$H NMR Δδ values of complex **3@1**$_2$.

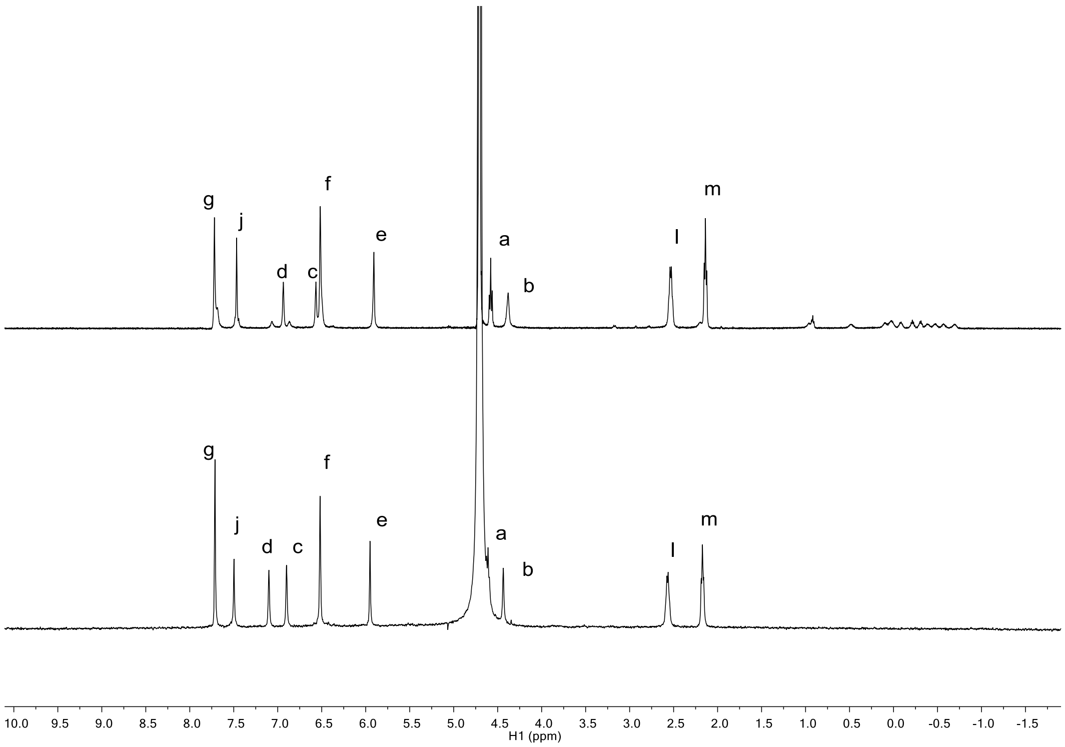


**Figure S9:** Stack $^1$H NMR of free host **2**(bottom) and the host-guest complex **3@2$_2$**(top).

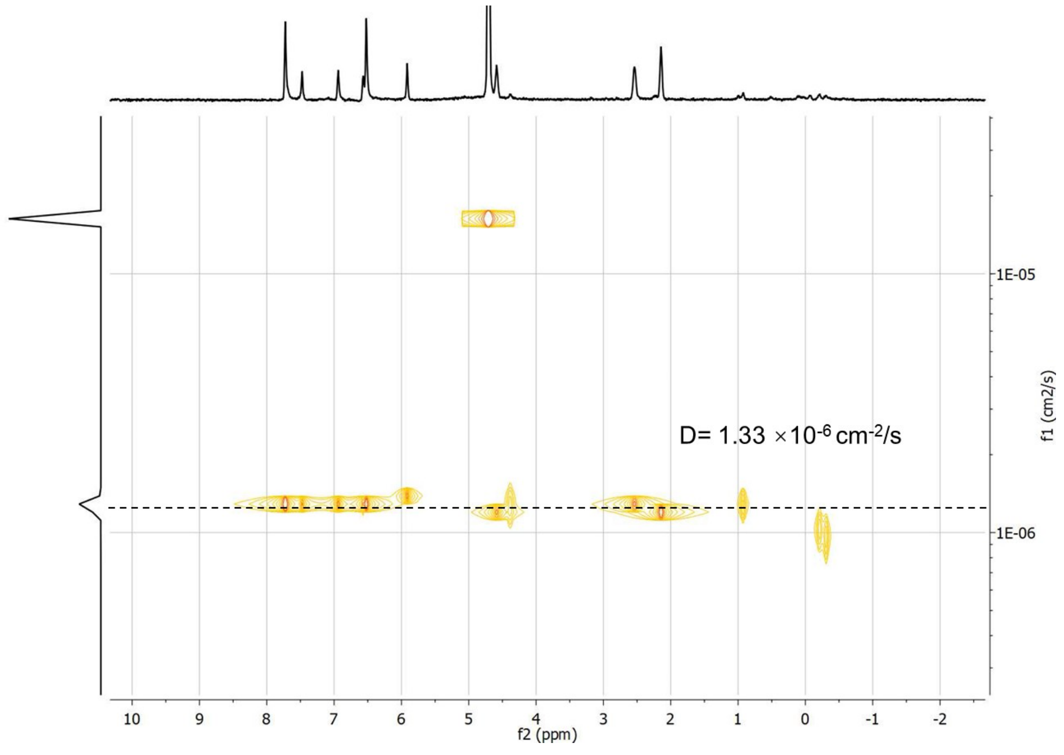


**Figure S10** DOSY NMR spectrum of complex **3@2$_2$**.

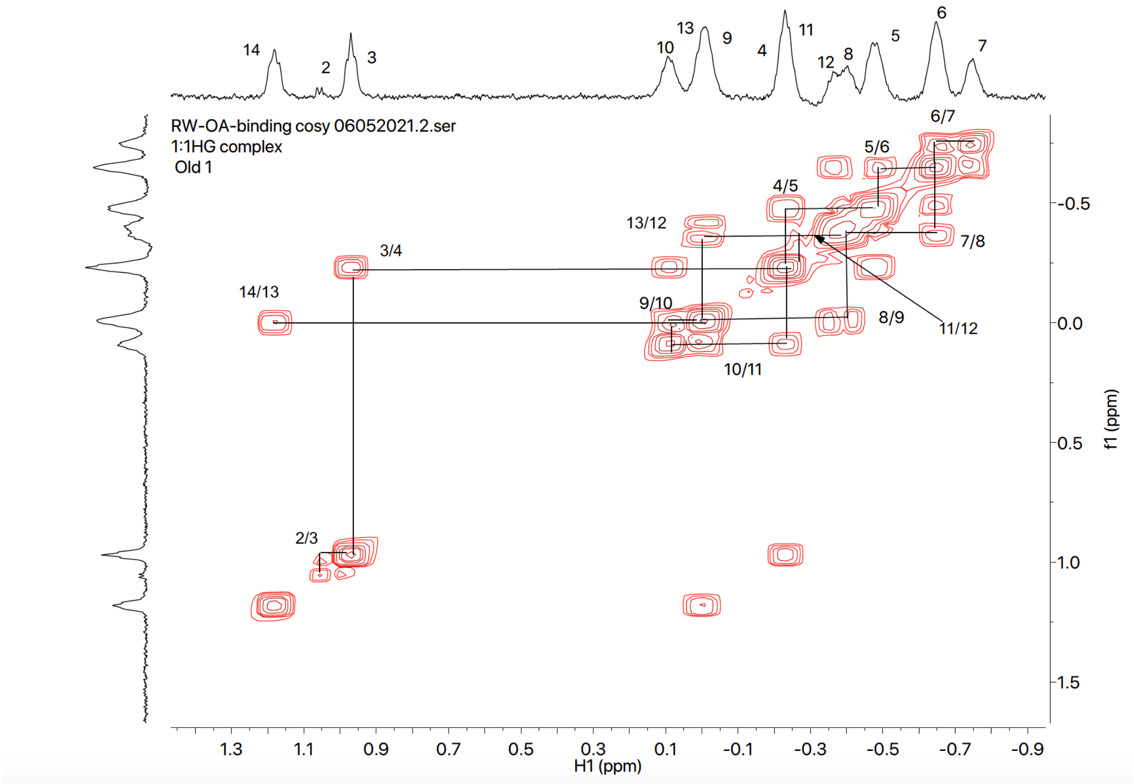


**Figure S11:** $^{1}H$-$^{1}H$ COSY NMR spectrum of the bound guest region and peak assignment of complex **3@2₂**.

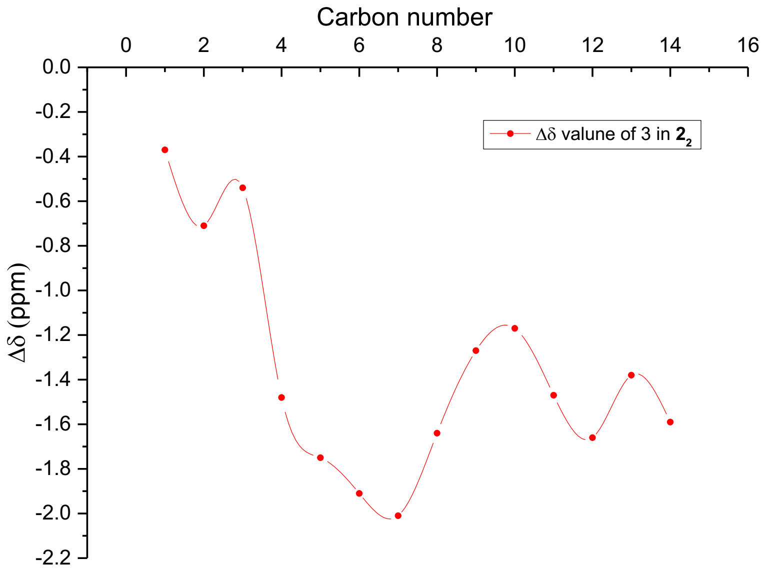


.

**Figure S12:** Plot of $^{1}H$ NMR Δδ values of complex **3@2$_2$**.

## 4) Eyring analysis for the cyclization of 3 within $\mathbf{1_2}$ and $\mathbf{2_2}$

In order to compare the kinetics of macrocyclization inside hosts **1** and **2**, i.e., we determined the logarithm of the reaction rate constant (*lnk*) as a function of temperature. The Eyring equation (Eq. 1) relates *ln(k/T)* to the reciprocal of the temperature (1/T):

$$ln\frac{k}{T} = \frac{-\Delta H^{\ddagger}}{R}.\frac{1}{T} + ln\frac{k_{\mathrm{B}}}{h} + \frac{\Delta S^{\ddagger}}{R} \qquad \textbf{(Eq. S1)}$$

Where $k$ is the reaction rate constant at temperature $T$ (Kelvin), $R$ is the gas constant (8.314 $J{\cdot}K^{-1}{\cdot}mol^{-1}$), $\Delta H^{\ddagger}$ is the activation enthalpy (kJ. $mol^{-1}$) and $\Delta S^{\ddagger}$ is the activation entropy (J. $mol^{-1}$. $K^{-1}$), $K_B$ is the Boltzmann constant ($1.380649 \times 10^{-23}$ $J.K^{-1}$) and h is the Planck constant ($6.63 \times 10^{-34}$ J·s). The experiments were performed between 339 K and 351 K for host **1**, and between 325 K to 338 K for host **2**.

The individual rate constants at each temperature were themselves determined using a first order kinetic model (Eq. S2):

$$y = A_1 e^{\left(\frac{-x}{t_1}\right)} + y_0 \qquad \textbf{(Eq. S2)}$$

Where y = the percentage conversion of the reactants, $A_1$ = amplitude fitting constant, $t_1$ = lifetime, $x$ = time, $y_0$ = $y$-offset at $t$ = 0), and the first-order rate constant $k$ is the inverse of the determined lifetime $t_1$. Thus, monitoring the appearance of the new $^1$H NMR signal from the $H_d$ proton (Figure S1) in the spectrum of both hosts gave the extent of reaction as a function of time. Individual rate constants were determined at least in duplicate, with an obtained error of <10%. **Figures S13 – S24** show: 1) the stacked $^1$H NMR spectroscopy of each reaction as a function of time at different temperatures; 2) the change in integration of the $H_d$ signal and the fit of this data using **Eq. S2** (all experiments were duplicated or triplicated, with the average or standard deviation shown as error bars), and 3) the Eyring plots for the cyclization processes within $\mathbf{1_2}$ and $\mathbf{2_2}$. Table **1** shows the obtained Eyring data for cyclization. The error in the gradient was calculated (Excel, LINEST function) to be 5%.

**Table 1.** Eyring data for the cyclization of guest **3** within the capsules $\mathbf{1_2}$ and $\mathbf{2_2}$.

| | **3 in $\mathbf{1_2}$** | **3 in $\mathbf{2_2}$** |
|---|---|---|
| $k$ ($s^{-1,}$ 338 K) | $3.47 \times 10^{-5}$ | $6.25 \times 10^{-5}$ |
| $k$ ($s^{-1,}$ 298.15 K) | $1.77 \times 10^{-6}$ | $1.58 \times 10^{-6}$ |
| Half-life (s, 338 K) [a] | $2.00 \times 10^{4}$ | $1.10 \times 10^{4}$ |
| Half-life (s, 298.15 K) [a] | $3.90 \times 10^{5}$ | $4.38 \times 10^{5}$ |
| $\Delta G^{\ddagger}$ (kJ $mol^{-1}$) | 105.8 | 106.2 |
| $\Delta H^{\ddagger}$ (kJ $mol^{-1}$) | 59.8 | 74.5 |
| $-T\Delta S^{\ddagger}$ (kJ $mol^{-1}$) | 45.9 | 31.7 |

## 4.1) Reaction of **3** within $\mathbf{1}_2$ at different temperatures and data fitting.

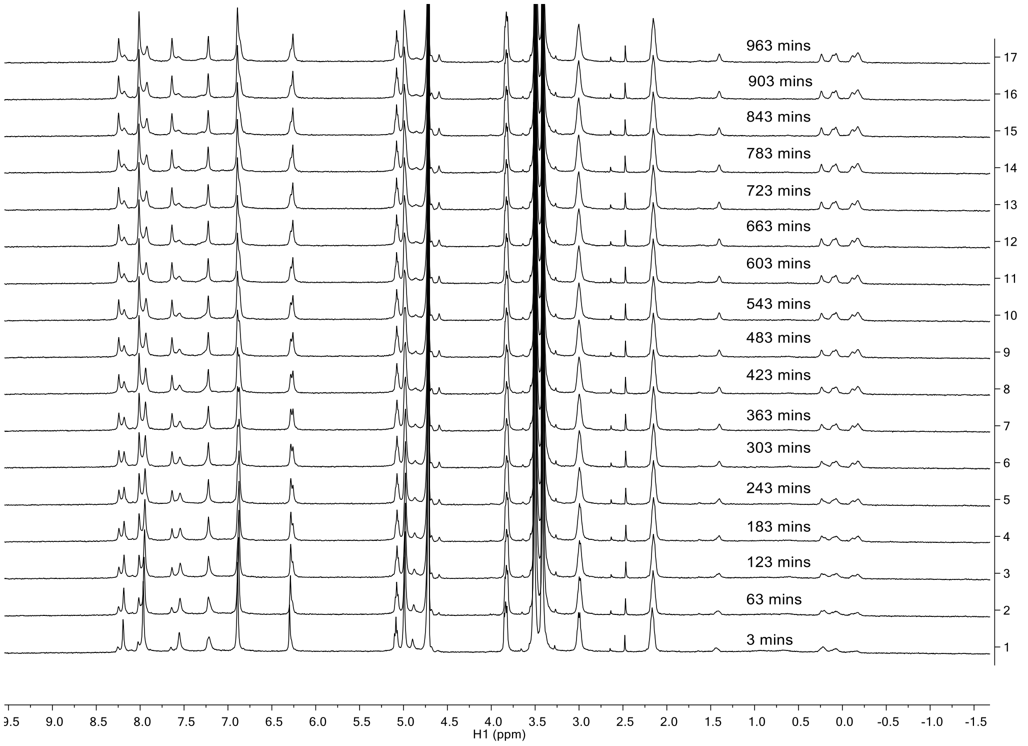


**Figure S13:** Stack of $^{1}$H NMR spectra showing reaction of encapsulated guest **3** inside of $\mathbf{1}_2$ as a function of time. ($D_2O$, 339 K, [Host **1**] = 1 mM, [Guest **3**] = 0.5 mM, [NaOD] =10 mM).

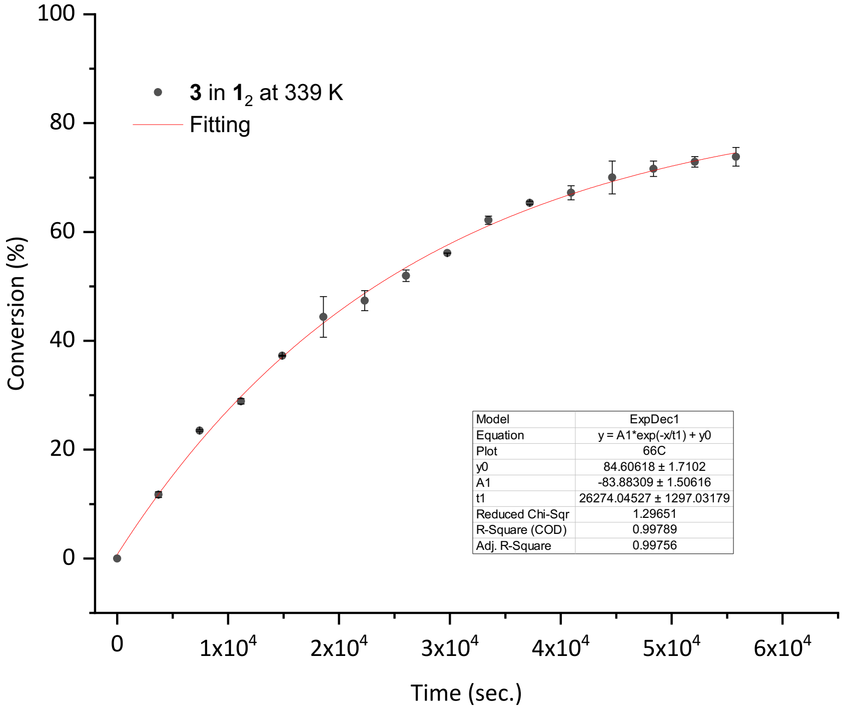


**Figure S14:** Conversion as a function of time plot for the reaction of guest **3** inside of host $\mathbf{1}_2$ at 339 K.

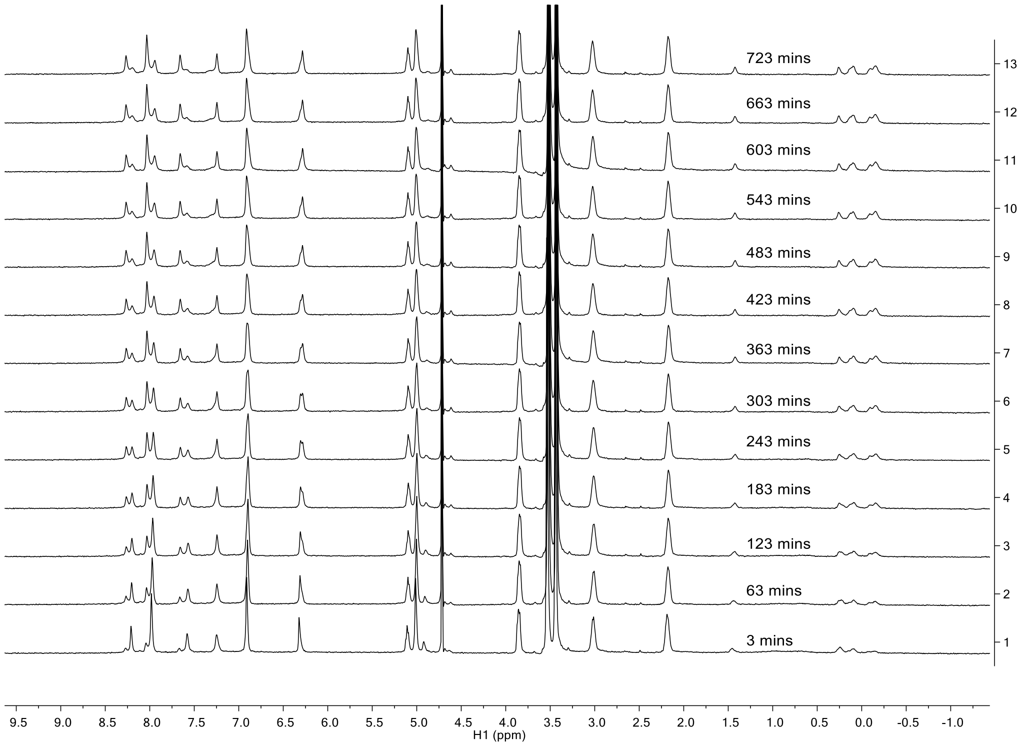


**Figure S15:** Stack of $^1$H NMR spectra showing reaction of encapsulated guest **3** inside of $\mathbf{1}_2$ as a function of time. ($D_2O$, 342 K, [Host **1**] = 1 mM, [Guest **3**] = 0.5 mM, [NaOD] =10 mM).

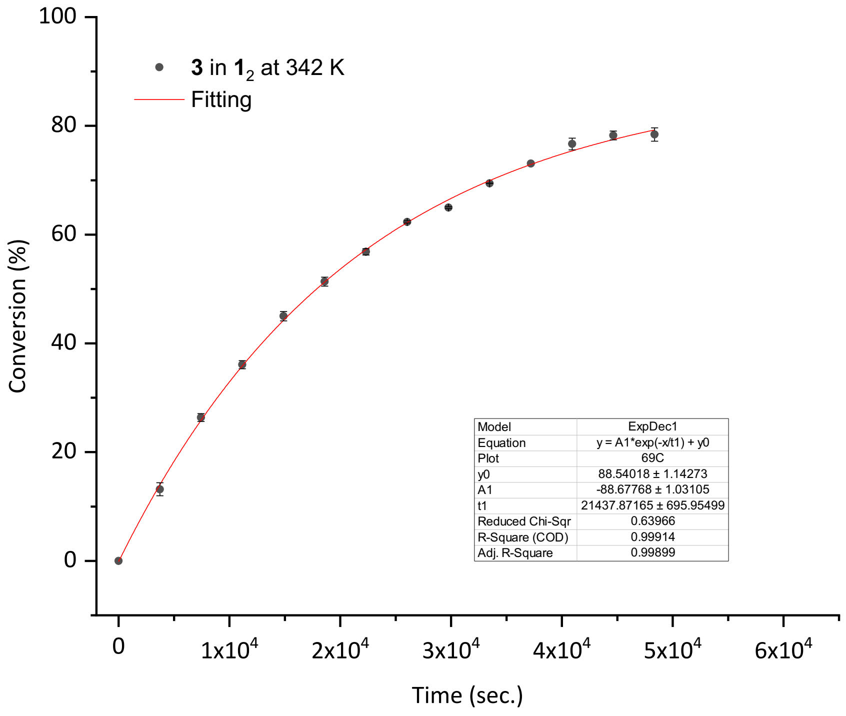


**Figure S16:** Conversion as a function of time plot for the reaction of guest **3** inside of host $\mathbf{1}_2$ at 342 K.

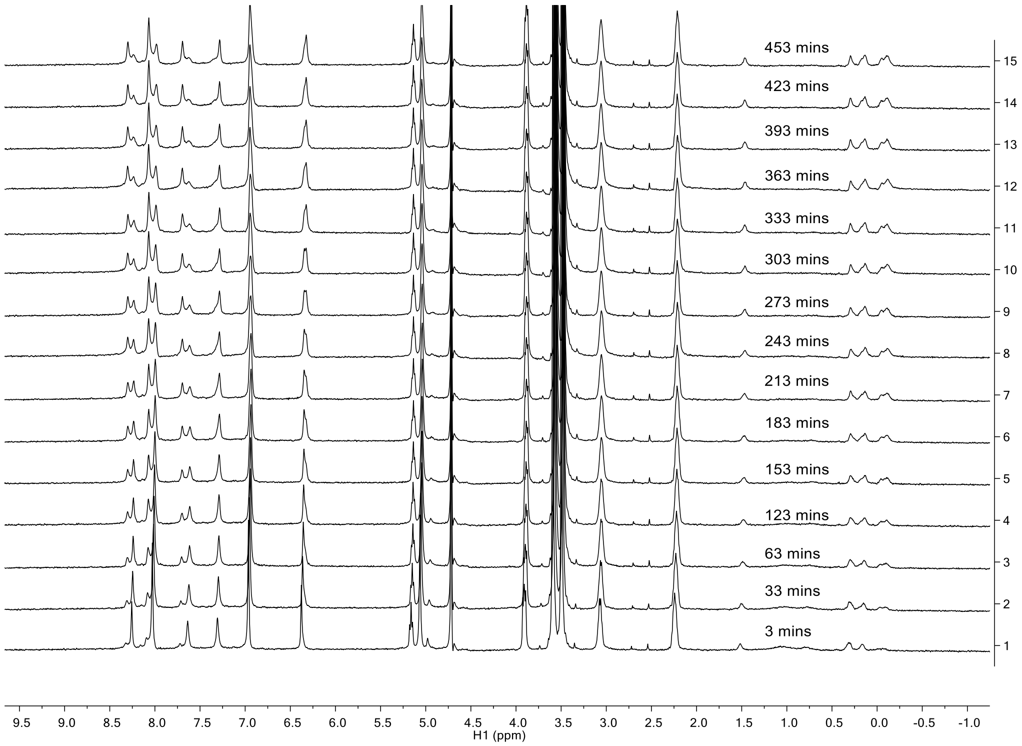


**Figure S17:** Stack of $^{1}$H NMR spectra showing reaction of encapsulated guest **3** inside of $\mathbf{1}_2$ as a function of time. ($D_2O$, 345 K, [Host **1**] = 1 mM, [Guest **3**] = 0.5 mM, [NaOD] =10 mM).

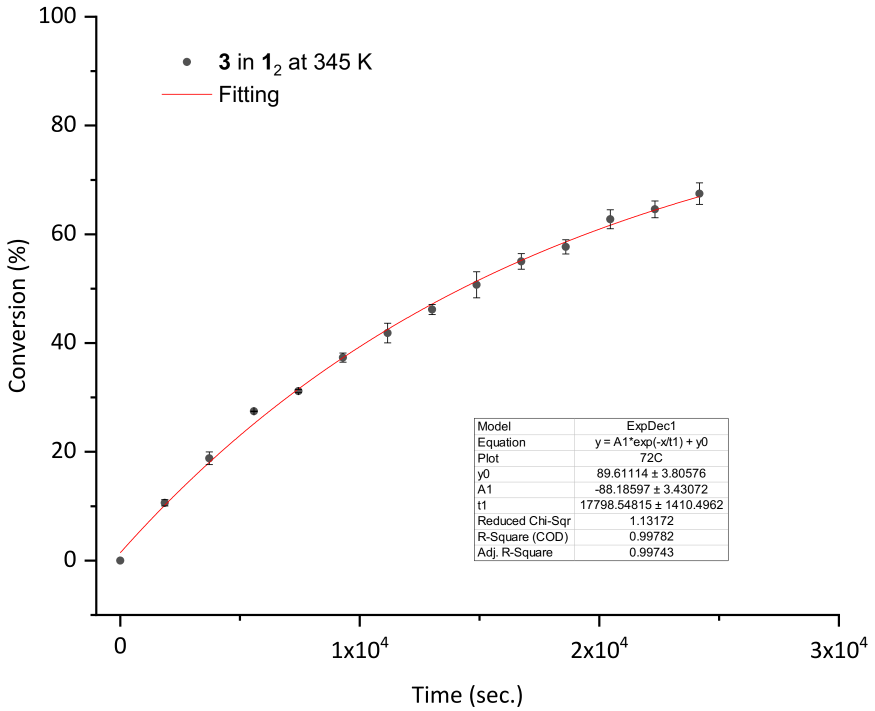


**Figure S18:** Conversion as a function of time plot for the reaction of guest **3** inside of host $\mathbf{1}_2$ at 345 K.

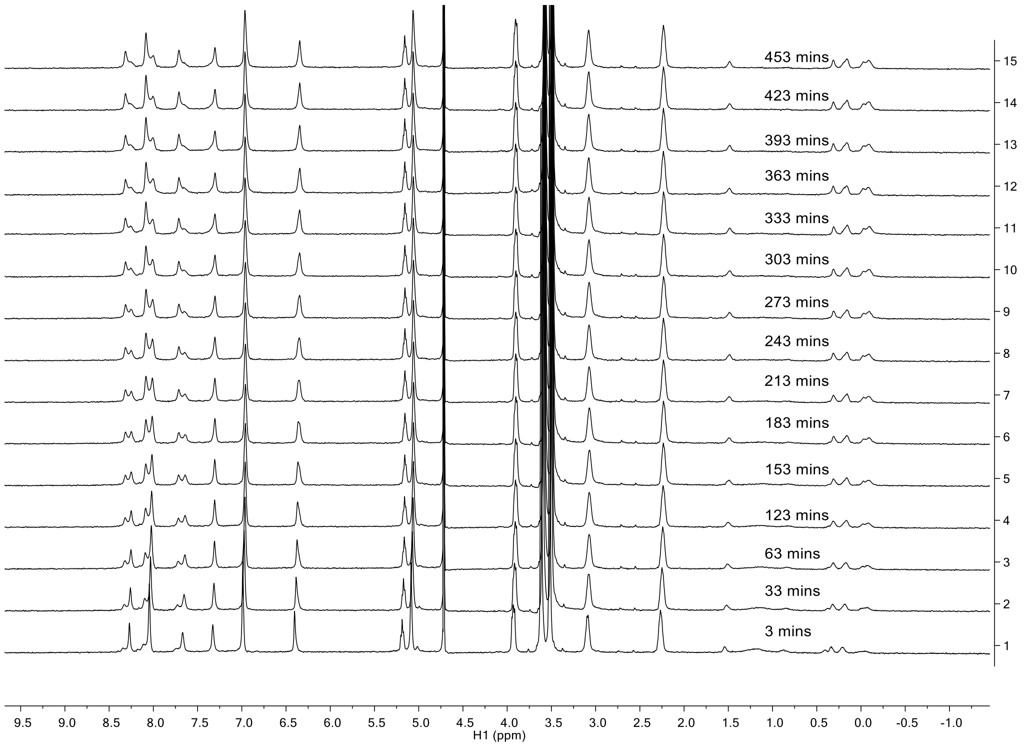


**Figure S19:** Stack of $^1$H NMR spectra showing reaction of encapsulated guest **3** inside of **1**$_2$ as a function of time. ($D_2O$, 348 K, [Host **1**] = 1 mM, [Guest **3**] = 0.5 mM, [NaOD] =10 mM).

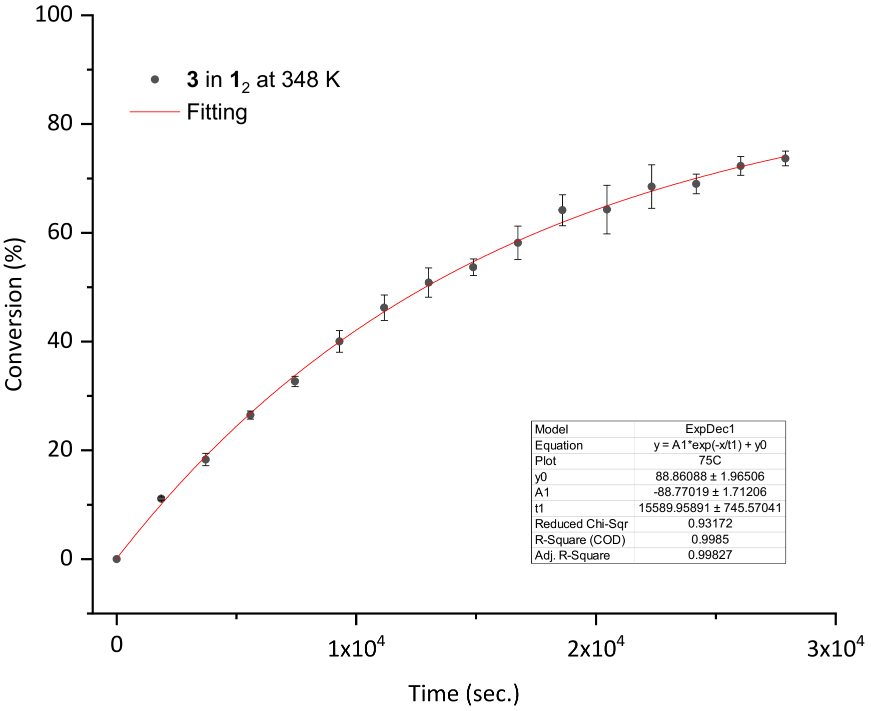


**Figure S20:** Conversion as a function of time plot for the reaction of guest **3** inside of host **1**$_2$ at 348 K.

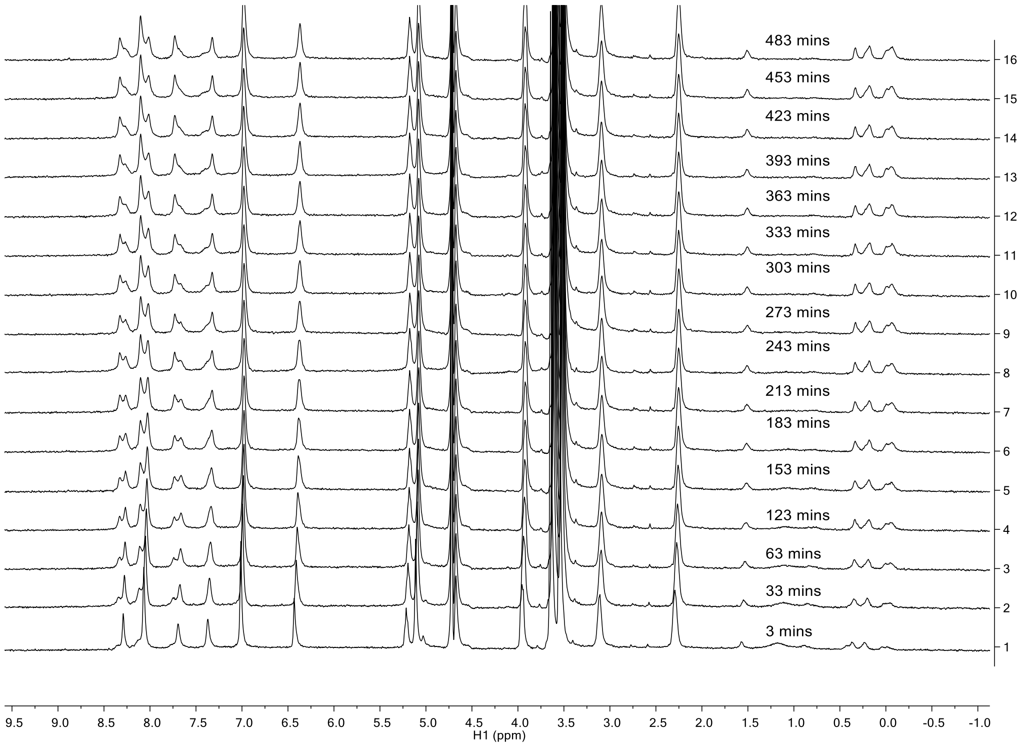


**Figure S21:** Stack of $^{1}$H NMR spectra showing reaction of encapsulated guest **3** inside of $\mathbf{1}_2$ as a function of time. ($D_2O$, 351 K, [Host **1**] = 1 mM, [Guest **3**] = 0.5 mM, [NaOD] =10 mM).

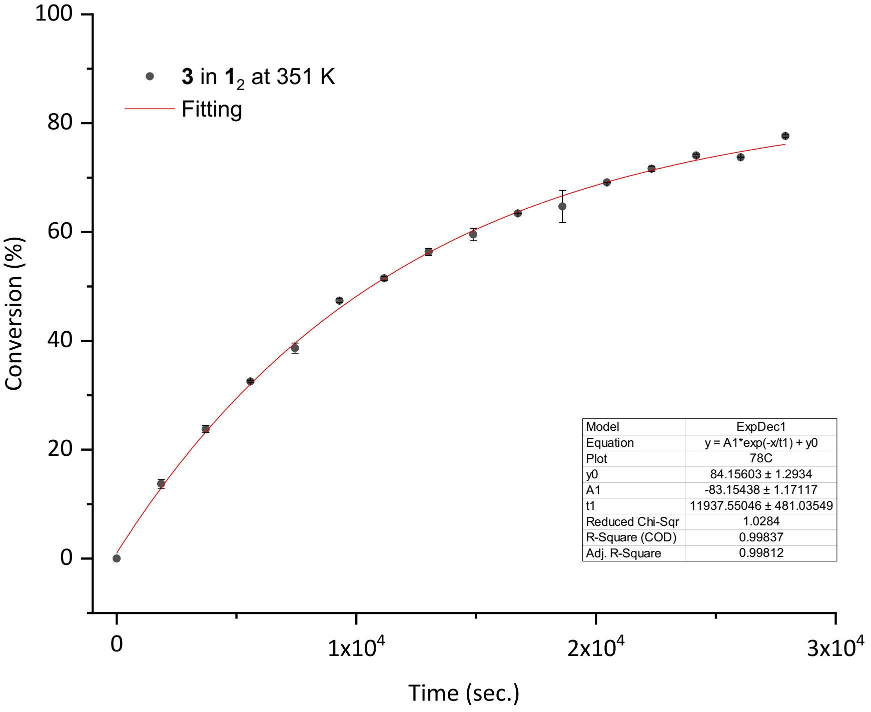


**Figure S22:** Conversion as a function of time plot for the reaction of guest **3** inside of host $\mathbf{1}_2$ at 351 K.

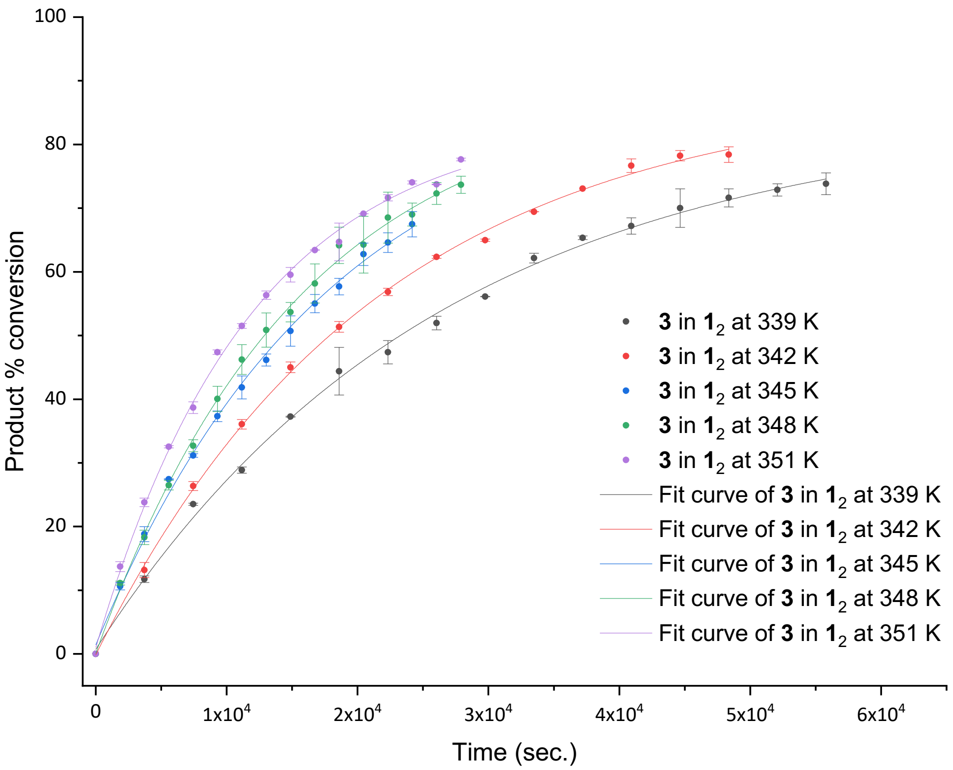


| Model | ExpDec1 | | | | |
|---|---|---|---|---|---|
| Equation | y = A1*exp(-x/t1) + y0 | | | | |
| Plot | 66C | 69C | 72C | 75C | 78C |
| y0 | 84.61 ± 1.71 | 88.54 ± 1.14 | 89.61 ± 3.81 | 88.86 ± 1.97 | 84.16 ± 1.29 |
| A1 | -83.88 ± 1.51 | -88.68 ± 1.03 | -88.19 ± 3.43 | -88.77 ± 1.71 | -83.15 ± 1.17 |
| **t1** | **2.63E+04 ± 1.30E+** | **2.14E+04 ± 6.96E** | **1.78E+04 ± 1.41E+** | **1.56E+04 ± 7.46E** | **1.19E+04 ± 4.81E** |
| Reduced Ch | 1.30 | 0.64 | 1.13 | 0.93 | 1.03 |
| R-Square (C | 1.00 | 1.00 | 1.00 | 1.00 | 1.00 |
| Adj. R-Squar | 1.00 | 1.00 | 1.00 | 1.00 | 1.00 |

**Figure S23:** Overall conversions as a function of time for the reaction of guest **3** inside of **1**$_2$ at five different temperatures (*top*), and summary of kinetic data fitting to Eq.2 (*bottom*).

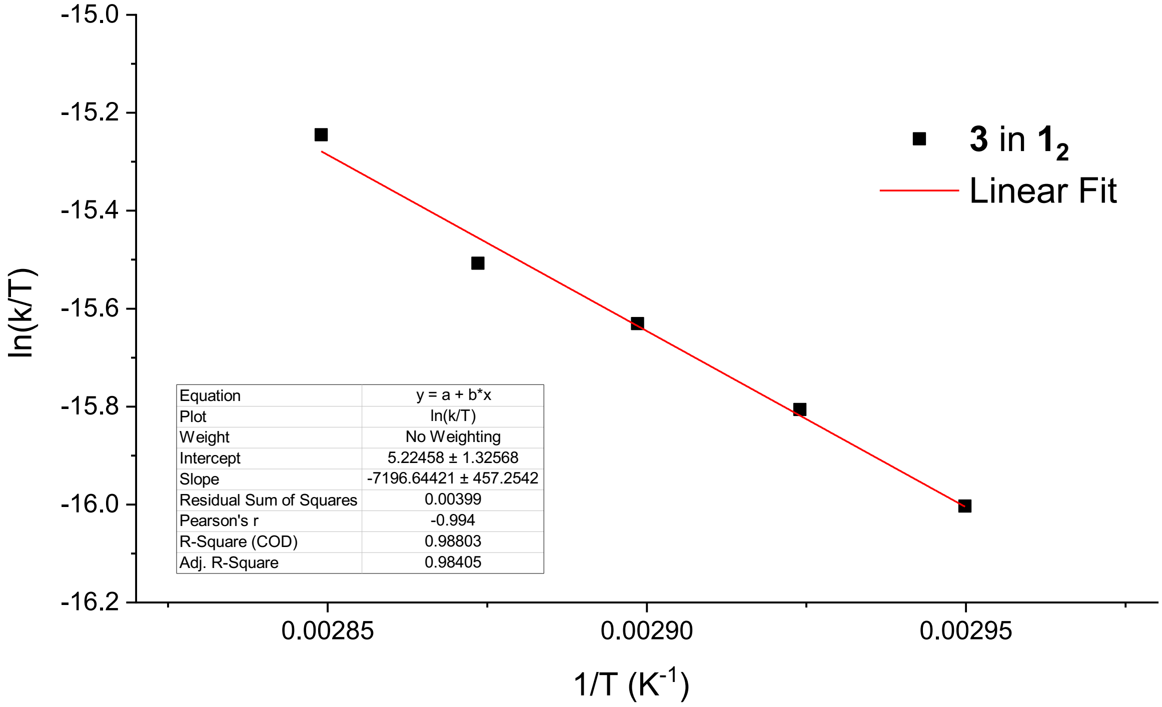


**Figure S24:** Eyring plot (Eq. 1) for the reaction of guest **3** in $\mathbf{1}_2$ at five different temperatures.

4.2) Reaction of **3** within **2**$_2$ at different temperature and data fitting.

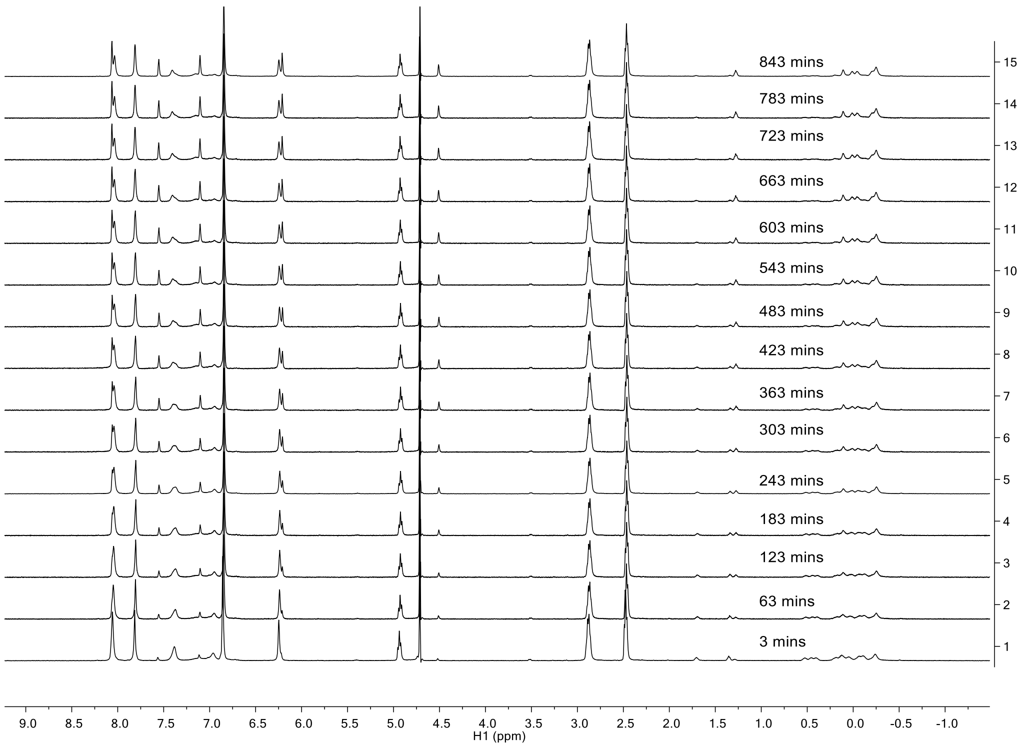


**Figure S25:** Stack of $^{1}$H NMR spectra showing reaction of encapsulated guest **3** inside of **2**$_2$ as a function of time. ($D_2O$, 325 K, [Host **2**] = 1 mM, [Guest **3**] = 0.5 mM, [NaOD] =10 mM).

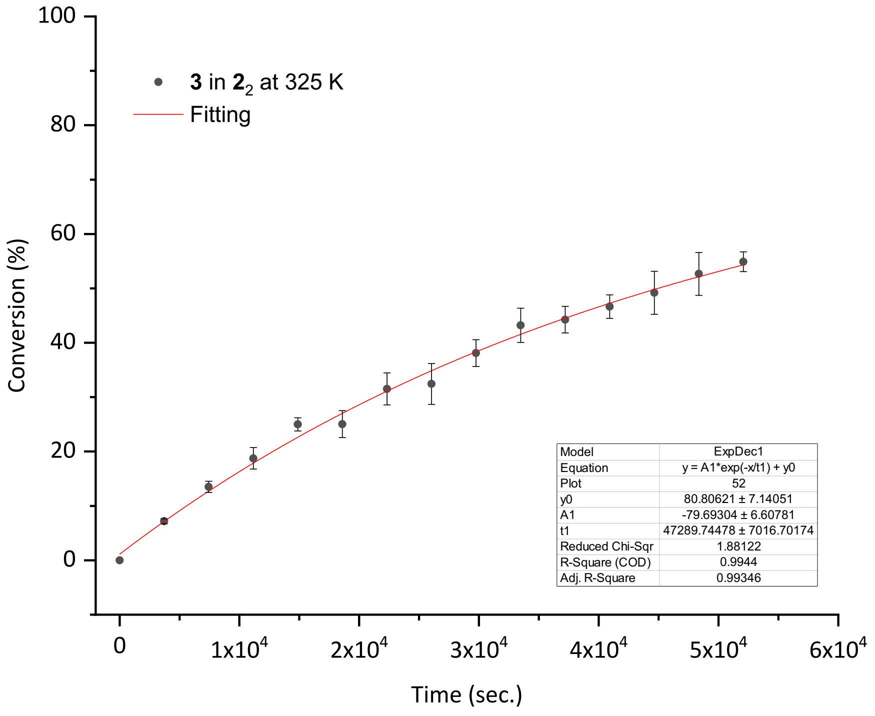


**Figure S26:** Conversion as a function of time plot for the reaction of guest **3** inside of host **2**$_2$ at 325 K.

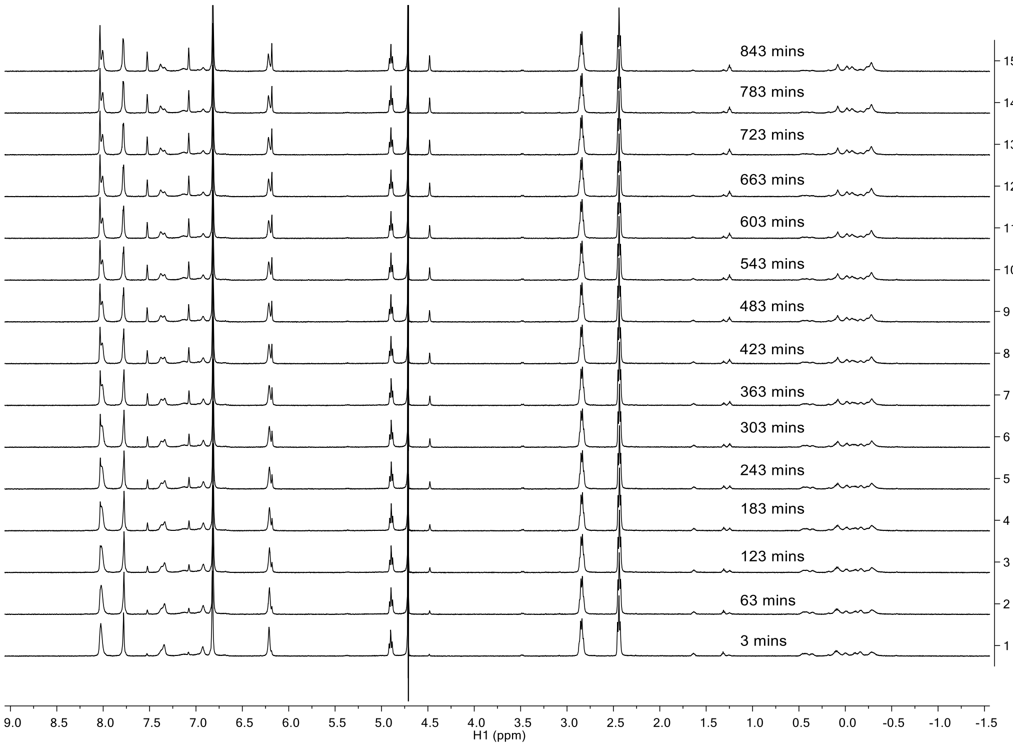


**Figure S27:** Stack of $^{1}H$ NMR spectra showing reaction of encapsulated guest **3** inside of $\mathbf{2}_2$ as a function of time. ($D_2O$, 328 K, [Host **2**] = 1 mM, [Guest **3**] = 0.5 mM, [NaOD] =10 mM).

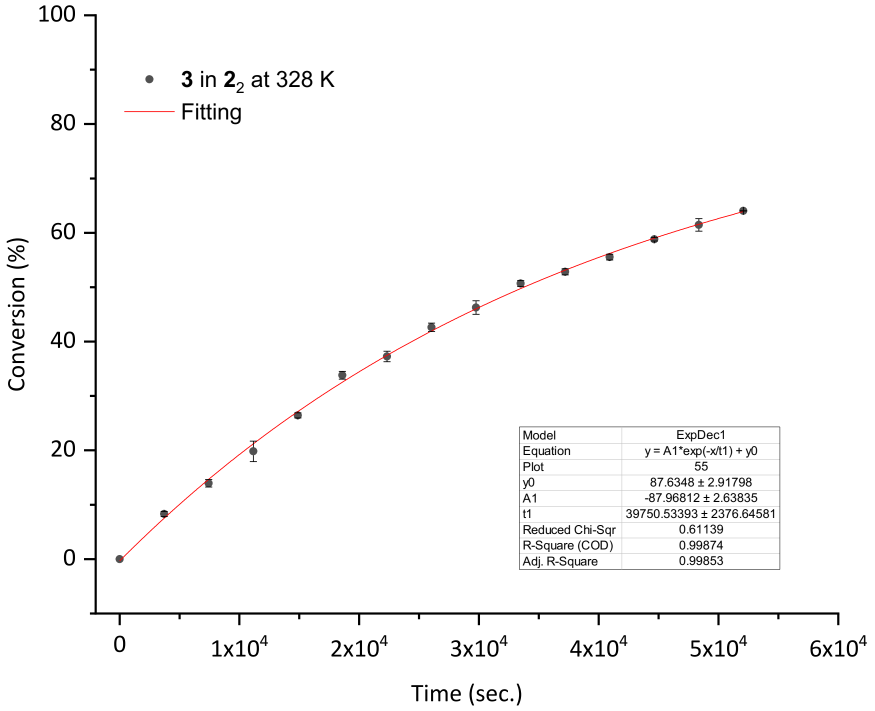


**Figure S28:** Conversion as a function of time plot for the reaction of guest **3** inside of host $\mathbf{2}_2$ at 328 K.

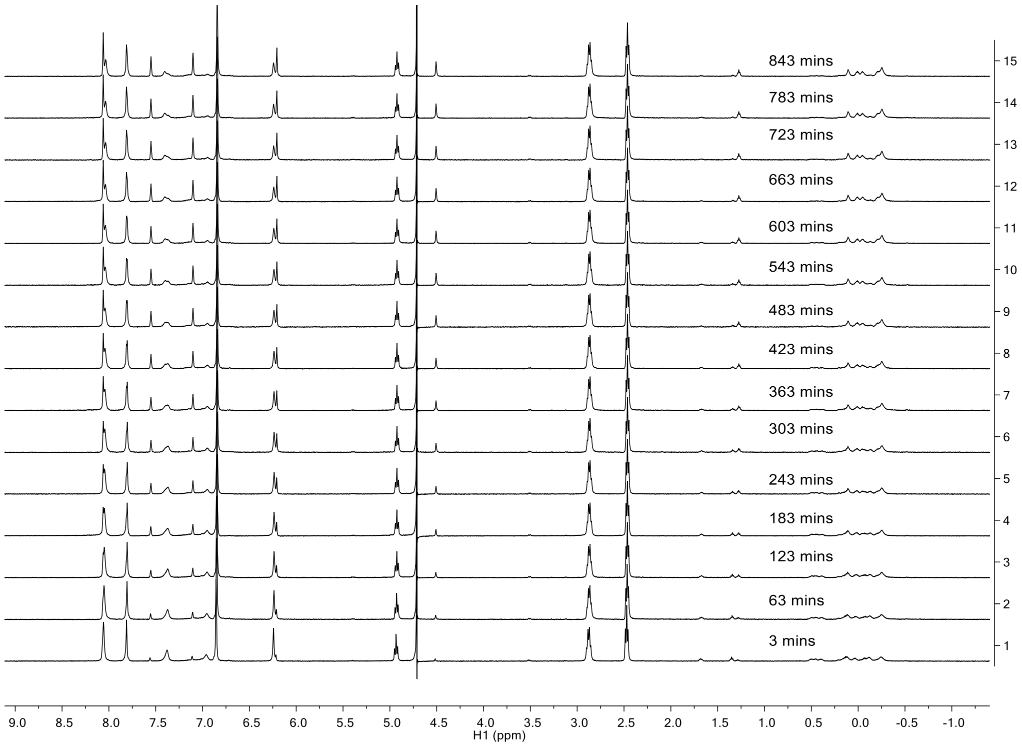


**Figure S29:** Stack of $^{1}$H NMR spectra showing reaction of encapsulated guest **3** inside of **2**$_2$ as a function of time. ($D_2O$, 331 K, [Host **2**] = 1 mM, [Guest **3**] = 0.5 mM, [NaOD] =10 mM).

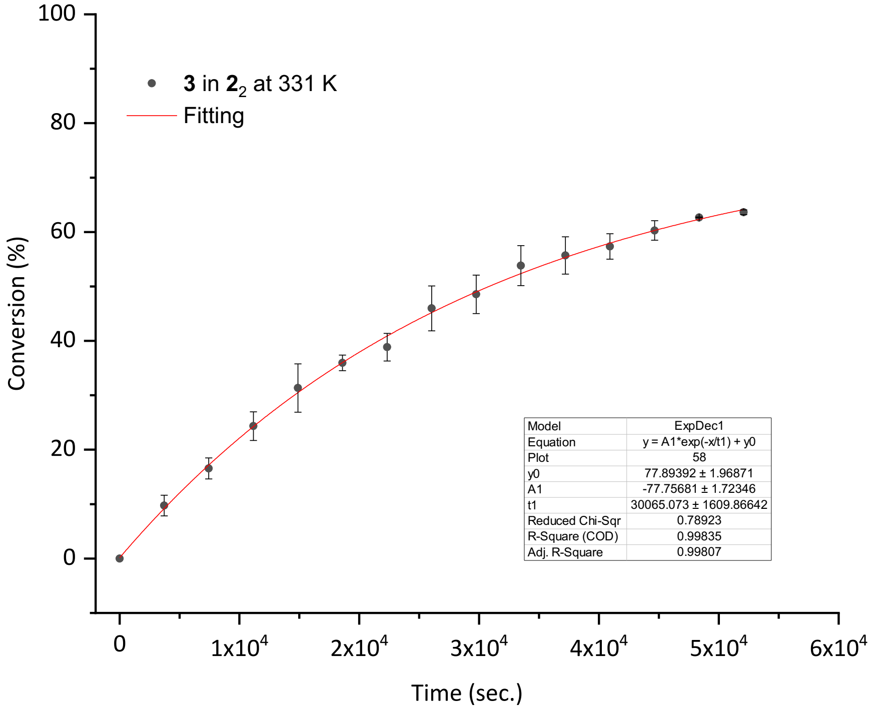


**Figure S30:** Conversion as a function of time plot for the reaction of guest **3** inside of host **2**$_2$ at 331 K.

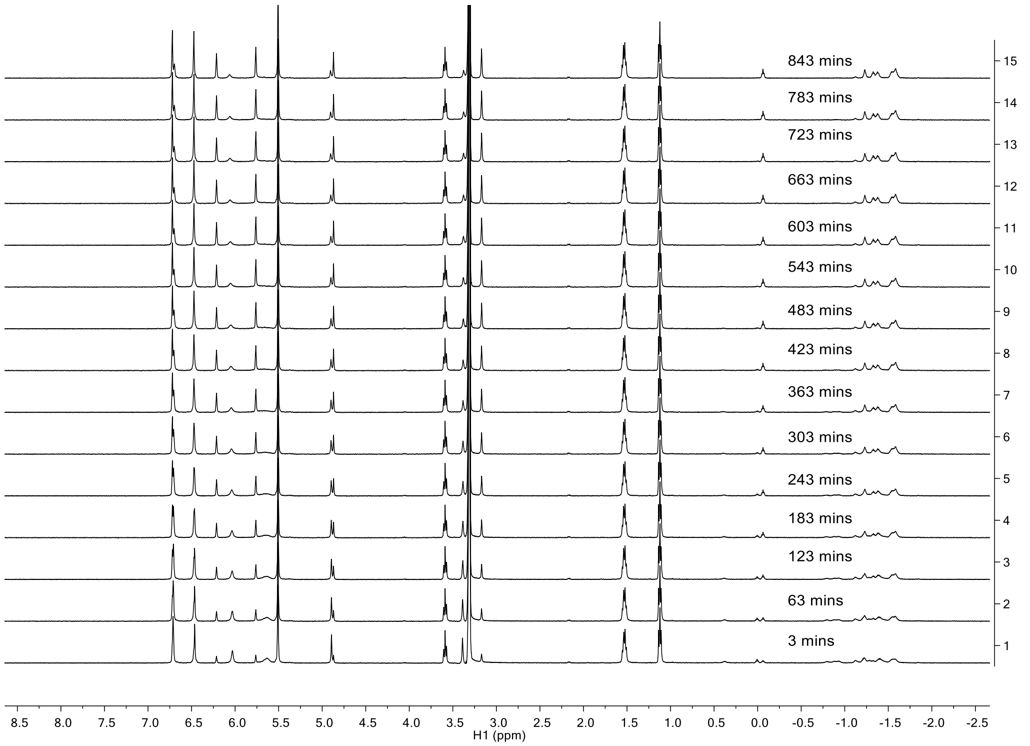


**Figure S31:** Stack of $^{1}$H NMR spectra showing reaction of encapsulated guest **3** inside of **2**$_2$ as a function of time. ($D_2O$, 335 K, [Host **2**] = 1 mM, [Guest **3**] = 0.5 mM, [NaOD] =10 mM).

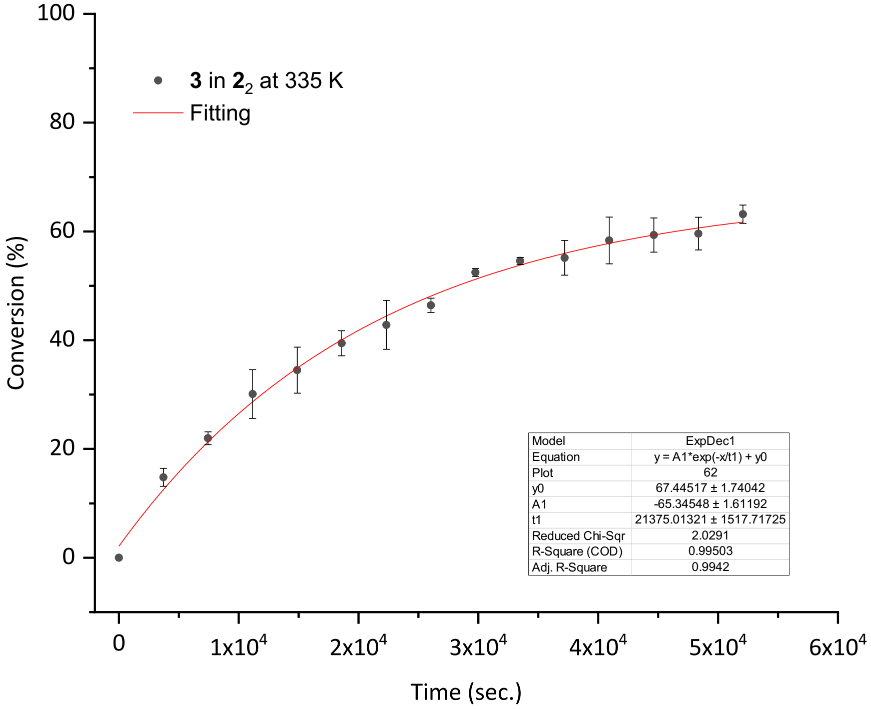


**Figure S32:** Conversion as a function of time plot for the reaction of guest **3** inside of host **2**$_2$ at 335 K.

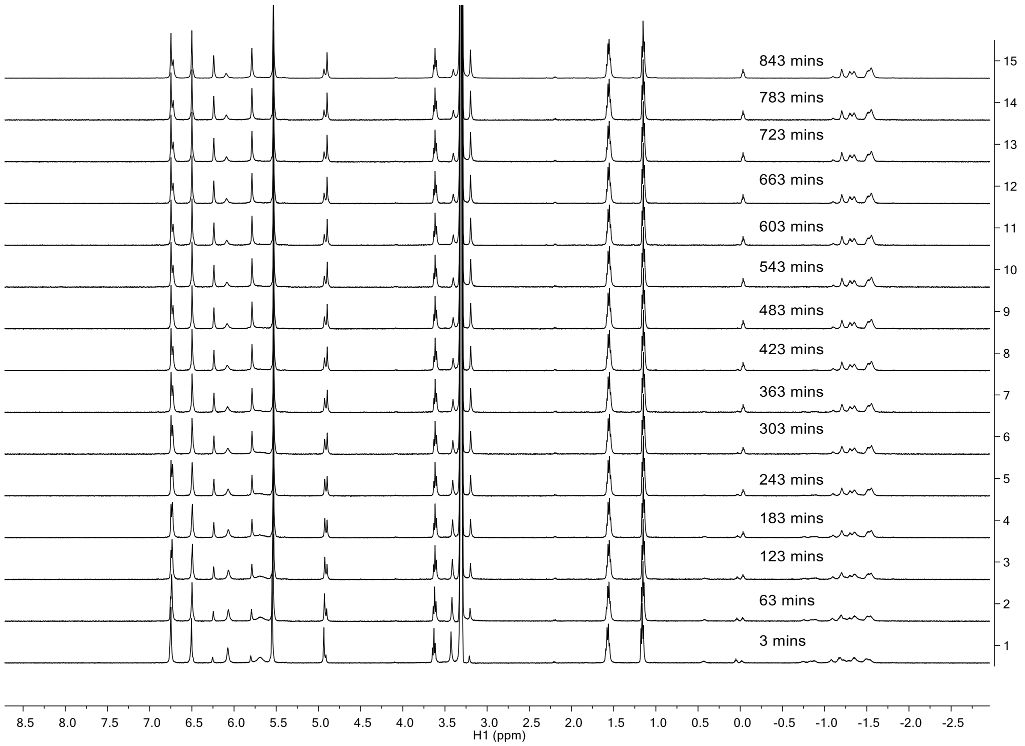


**Figure S33:** Stack of $^{1}$H NMR spectra showing reaction of encapsulated guest **3** inside of **2**$_2$ as a function of time. ($D_2O$, 338 K, [Host **2**] = 1 mM, [Guest **3**] = 0.5 mM, [NaOD] =10 mM).

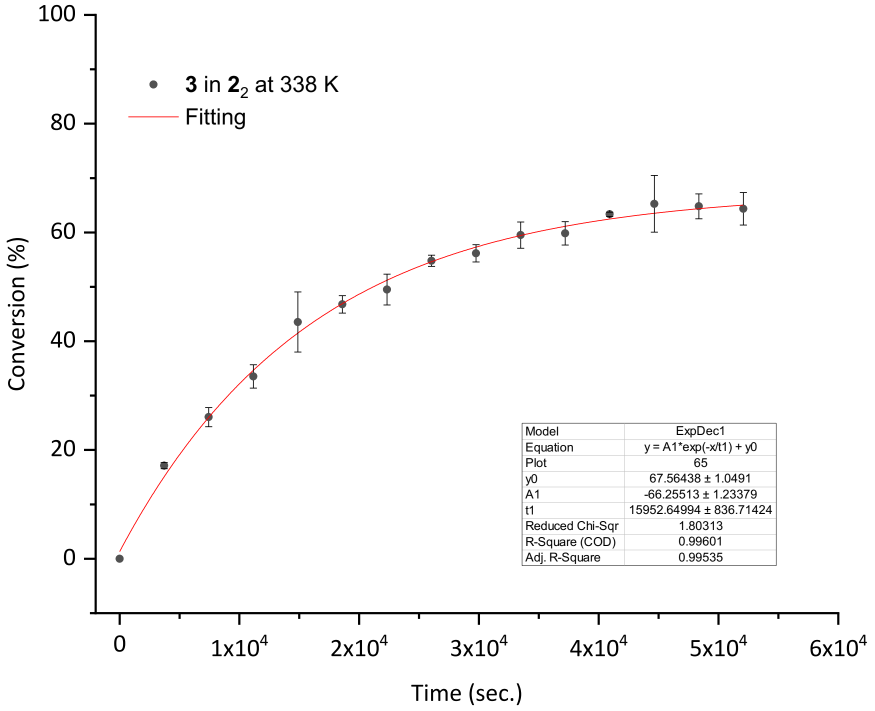


**Figure S34:** Conversion as a function of time plot for the reaction of guest **3** inside of host **2**$_2$ at 338K.

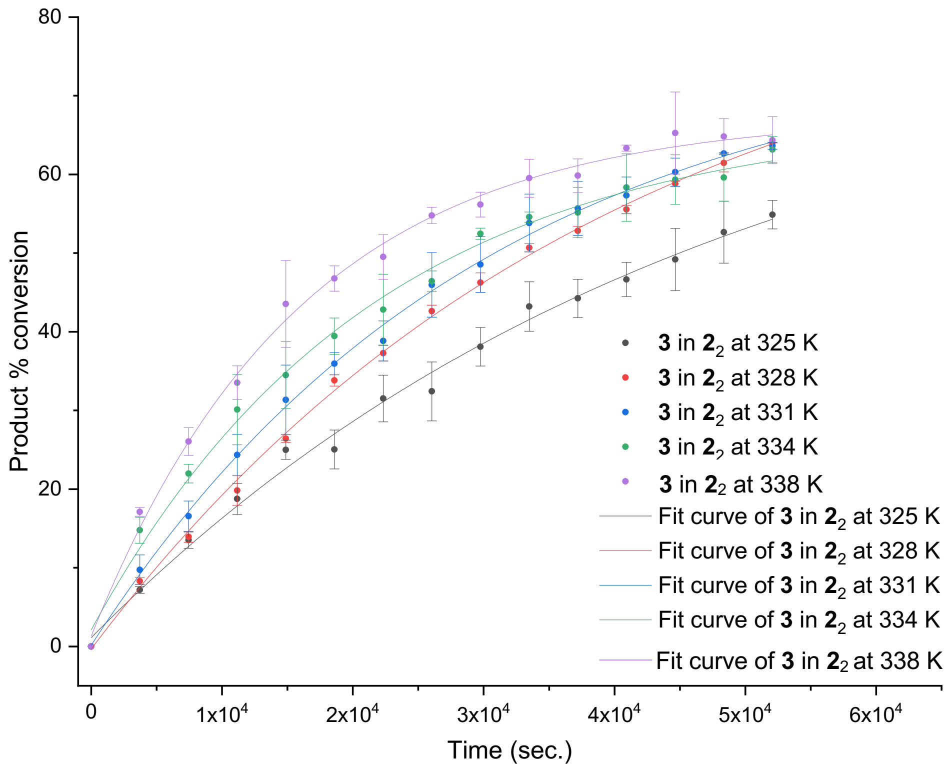


| Model | ExpDec1 | | | | |
|---|---|---|---|---|---|
| Equation | y = A1*exp(-x/t1) + y0 | | | | |
| Plot | 52 | 55 | 58 | 62 | 65 |
| y0 | 80.80621 ± 7.14051 | 87.6348 ± 2.91798 | 77.89392 ± 1.968 | 67.44517 ± 1.74042 | 67.56438 ± 1.0491 |
| A1 | -79.69304 ± 6.6078 | -87.96812 ± 2.6383 | -77.75681 ± 1.723 | -65.34548 ± 1.6119 | -66.25513 ± 1.233 |
| **t1** | **4.73E+04 ± 7.02E+** | **3.98E+04 ± 2.38E+** | **3.01E+04 ± 1.61E** | **2.14E+04 ± 1.52E+** | **1.60E+04 ± 8.37E** |
| Reduced Ch | 1.88122 | 0.61139 | 0.78923 | 2.0291 | 1.80313 |
| R-Square (C | 0.9944 | 0.99874 | 0.99835 | 0.99503 | 0.99601 |
| Adj. R-Squar | 0.99346 | 0.99853 | 0.99807 | 0.9942 | 0.99535 |

**Figure S35:** Overall conversions as a function of time for the reaction of guest **3** inside of **2$_2$** at five different temperatures (*top*), and summary of kinetic data fitting to Eq. 2 (*bottom*).

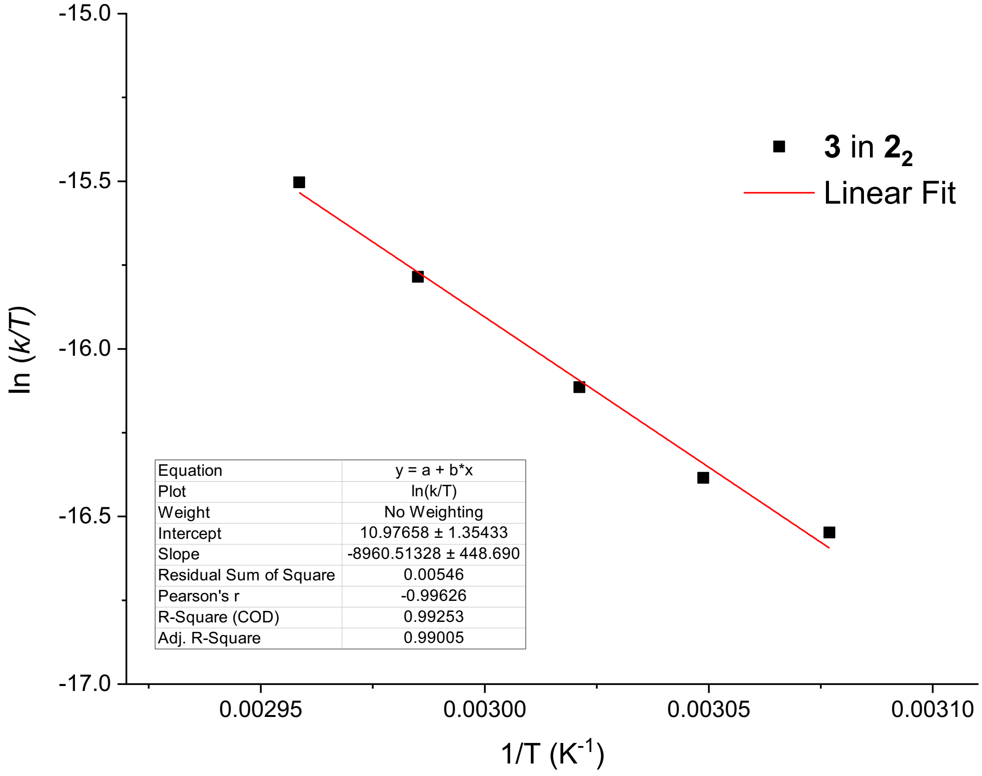


**Figure S36:** Eyring plot (Eq. 1) for the reaction of guest **3** in $\mathbf{2}_2$ at five different temperatures.

## 5) Cyclization of 3 within $1_2$ in the presence of various salts

Previous studies have shown that anions can bind to both the pendent groups or feet of host **1,** and the non-polar pocket.[7] Additionally, studies showed that the electrostatic potential field (EPF) of either host had a significant effect on reactions carried out in the inner-space of the capsule, and that this EPF was modulated by counter-ion binding to the outside of the host. We therefore investigated the cyclization rate of **3** in the presence of anions known to bind to **1**. To study this, the effect of nine different anions was investigated: $F^-$, $Cl^-$, $Br^-$, $I^-$, $ClO_4^-$, $CF_3SO_3^-$, $SCN^-$, $NO_3^-$, and $Cl_2CHCO_2^-$ ($DCA^-$).

**Figures S37** – **S59** show the corresponding data for reactions carried out with the same protocol but in the presence of 10 mM of the anions (as their sodium salts). The experiments were performed with 0.5 mL samples of 1.0 mM host **1** in $D_2O$ at 339 K. In each case, a small volume of 400 mM salt solution was added to make a 10 mM salt solution of the complex.

## 5.1) Anion effect: Summary of data

Figures S37 and S38 show the $^1$H NMR spectra of the complex with $\mathbf{1}_2$ in the presence of 10 mM salt.

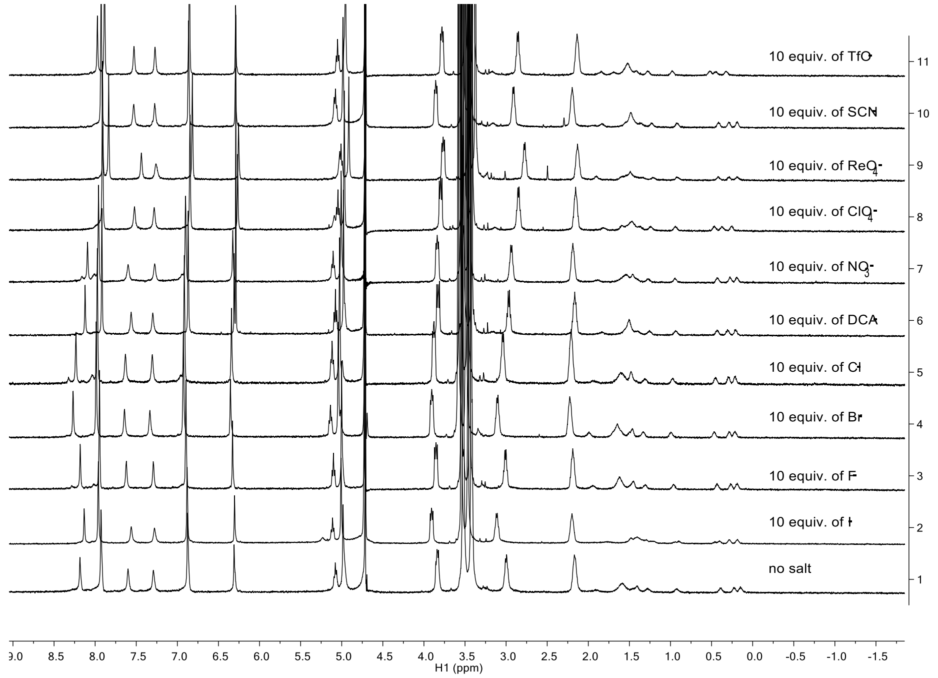


**Figure S37:** Stack of $^1$H NMR spectra of **3** in $\mathbf{1}_2$ in the presence of various of anion at 339 K.

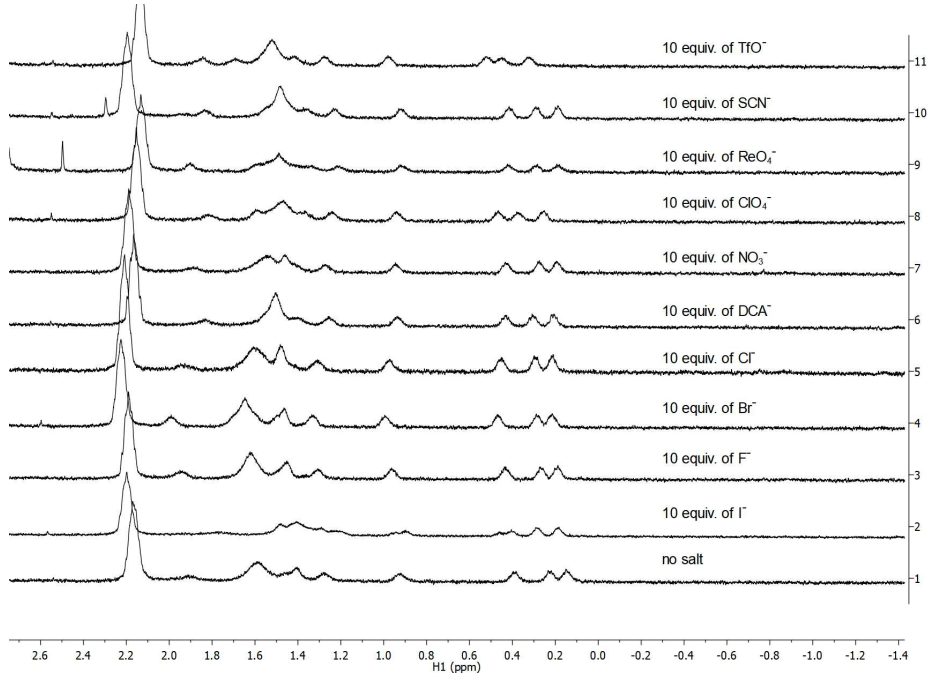


**Figure S38:** Stack of $^1$H NMR spectra of **3** in $\mathbf{1}_2$ in guest region in the presence of various of 10 equivalents of the indicated sodium salt at 339 K.

The extent of reaction of each experiment was determined by tracking and integrating the $^{1}$H NMR signal from the $H_d$ proton (Mnova) and fitting this data as a function of time to give the rate constant data (Origin Pro 2018. **Figure S39** shows the cumulative data for all the salts. Each experiment was carried out in duplicate (error bars shown), with the average calculated used to give the rate constant (Table 2). Errors were <10%.

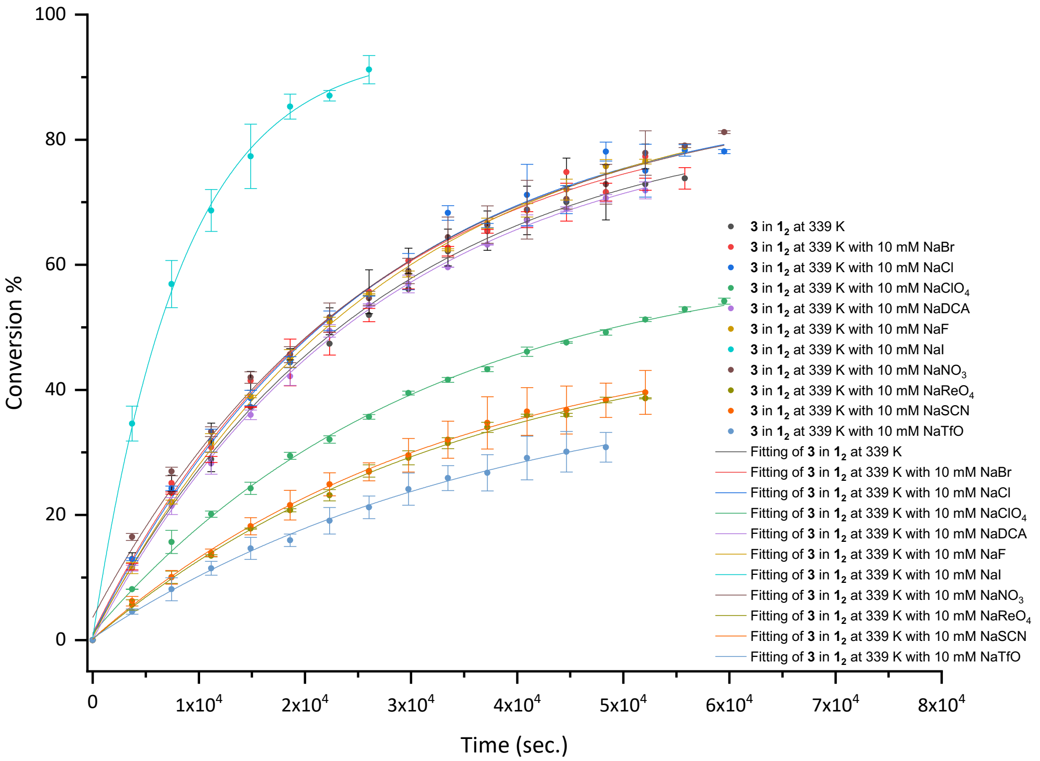


| Model | ExpDec1 | | | | | | | | | | |
|---|---|---|---|---|---|---|---|---|---|---|---|
| Equati | y = A1*exp(-x/t1) + y0 | | | | | | | | | | |
| Plot | P1 | Br | Cl | ClO4 | DCA | F | I | NO3 | ReO4 | SCN | TfO |
| y0 | 84.61 ± 1. | 85.55 ± 2. | 87.77 ± 2. | 62.39 ± 1. | 83.67 ± 1. | 89.37 ± 1 | 94.40 ± 1. | 88.13 ± 2. | 50.79 ± 1. | 50.54 ± 1. | 43.29 ± 1. |
| A1 | -83.88 ± 1. | -84.49 ± 2. | -86.97 ± 2. | -61.35 ± 1. | -83.57 ± 1 | -88.86 ± | -94.06 ± | -84.52 ± 2 | -50.68 ± 1. | -50.34 ± 1. | -42.99 ± 1. |
| **t1** | **26274.05** | **24578.43** | **25630.52** | **30768.66** | **26065.98** | **27075.47** | **8344.58 ±** | **26601.09** | **34990.47** | **33571.86** | **38064.53** |
| Reduc | 1.30 | 2.71 | 3.44 | 0.45 | 0.48 | 0.69 | 1.36 | 3.65 | 0.31 | 0.25 | 0.24 |
| R-Squ | 1.00 | 1.00 | 0.99 | 1.00 | 1.00 | 1.00 | 1.00 | 0.99 | 1.00 | 1.00 | 1.00 |
| Adj. R- | 1.00 | 1.00 | 0.99 | 1.00 | 1.00 | 1.00 | 1.00 | 0.99 | 1.00 | 1.00 | 1.00 |

**Figure S39:** Overall conversions as a function of time for the reaction of guest **3** in $\mathbf{1}_2$ in the presence of 10 equiv. different anions at 339 K (*top*), and summary of kinetic parameters from fitting to Eq. 2 (*bottom*).

**Table 2:** The reaction rate (*k*) of **3** in **$1_2$** in the presence of different anions at 339 K.

| Na Salt | Rate Constant, *k* in $10^{-5}$ $s^{-1}$ |
|---|---|
| $I^-$ | 12.00 |
| $Br^-$ | 4.07 |
| $Cl^-$ | 3.91 |
| $DCA^-$ | 3.83 |
| no salt | 3.80 |
| $NO_3^-$ | 3.76 |
| $F^-$ | 3.69 |
| $ClO_4^-$ | 3.25 |
| $SCN^-$ | 2.98 |
| $ReO_4^-$ | 2.86 |
| $TfO^-$ | 2.62 |

## 5.2) Anion effect: Individual data

The reaction rate in each experiment was determined by tracking and integrating the "d" peak in the $^1$H NMR spectroscopy using Mnova, then fitted the integrations as a function of time to give the kinetic data with Origin Pro 2018. Lines fitted by the first order kinetic model are demonstrated in red and the standard deviations are shown as error bars. Each experiment was carried out in duplicates, with the average calculated to give the corresponding rate constants.

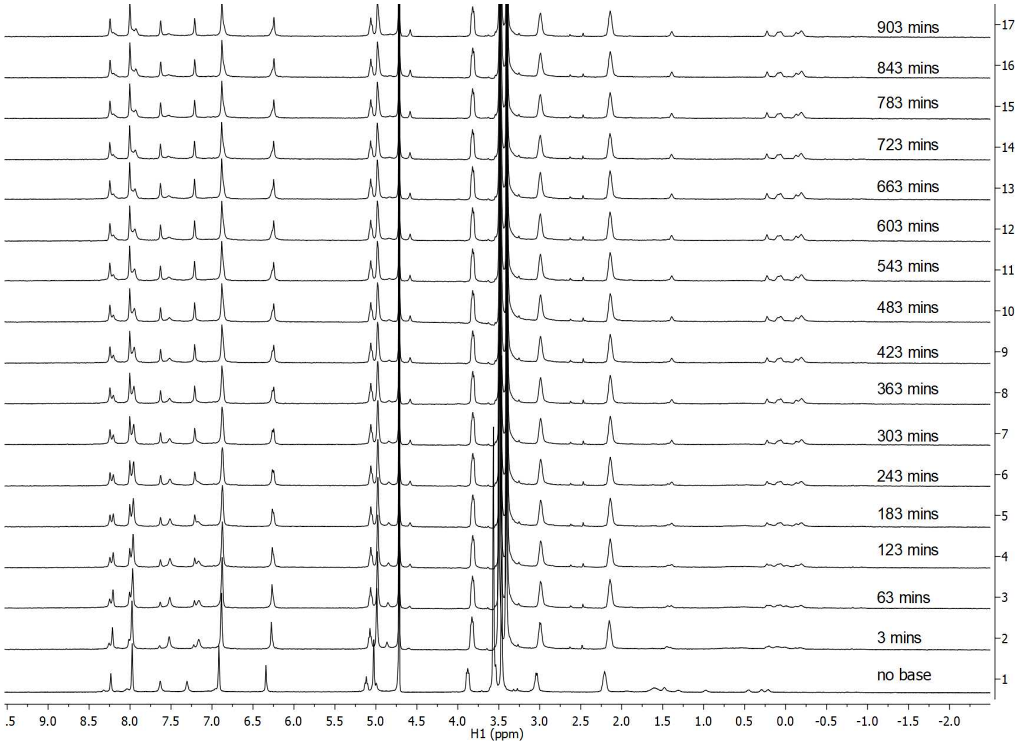


**Figure S40:** Stack of $^1$H NMR spectra showing reaction of encapsulated guest **3** inside of $\mathbf{1}_2$ with 10 equiv. NaCl as a function of time. ($D_2O$, 339 K, [Host **1**] = 1 mM, [Guest **3**] = 0.5 mM, [NaOD] =10 mM).

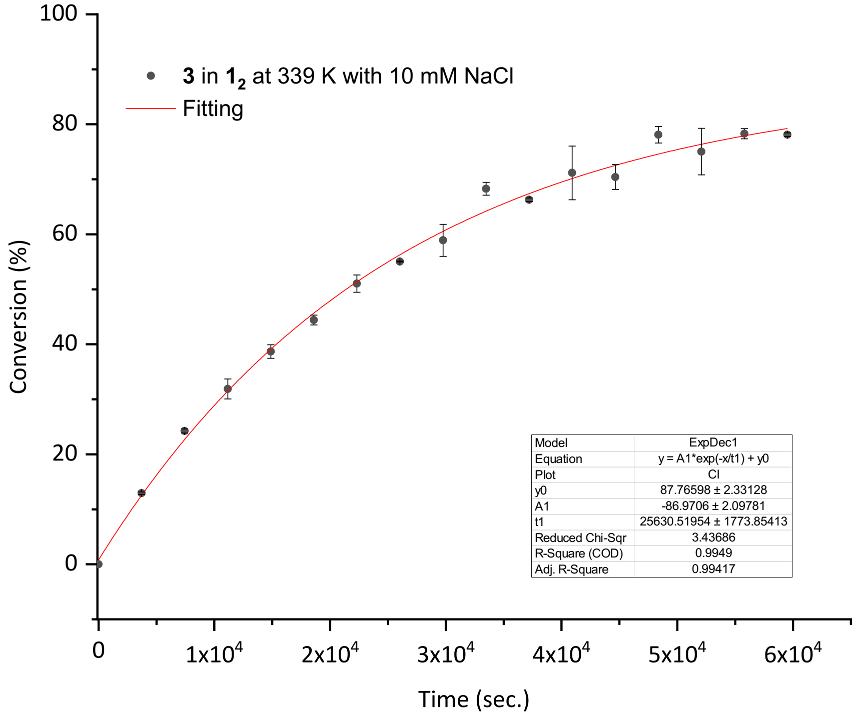


**Figure S41:** Conversion as a function of time plot for the reaction of guest **3** in $\mathbf{1}_2$ in the presence of 10 equiv. NaCl at 339 K.

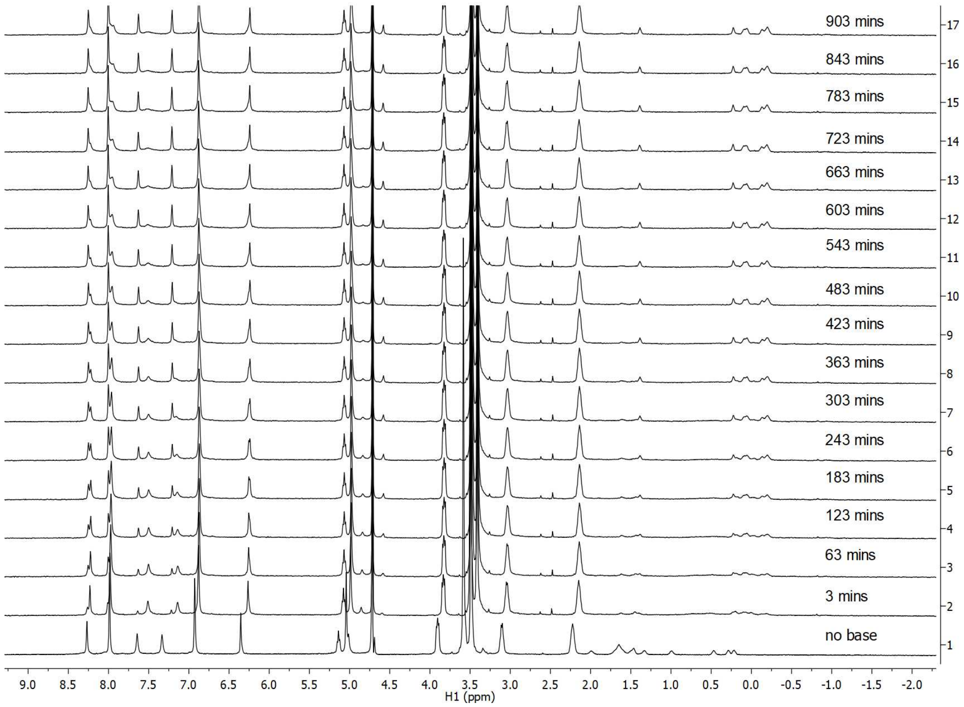


**Figure S42:** Stack of $^{1}$H NMR spectra showing reaction of encapsulated guest **3** inside of **1**$_2$ with 10 equiv. NaBr as a function of time. ($D_2O$, 339 K, [Host **1**] = 1 mM, [Guest **3**] = 0.5 mM, [NaOD] =10 mM).

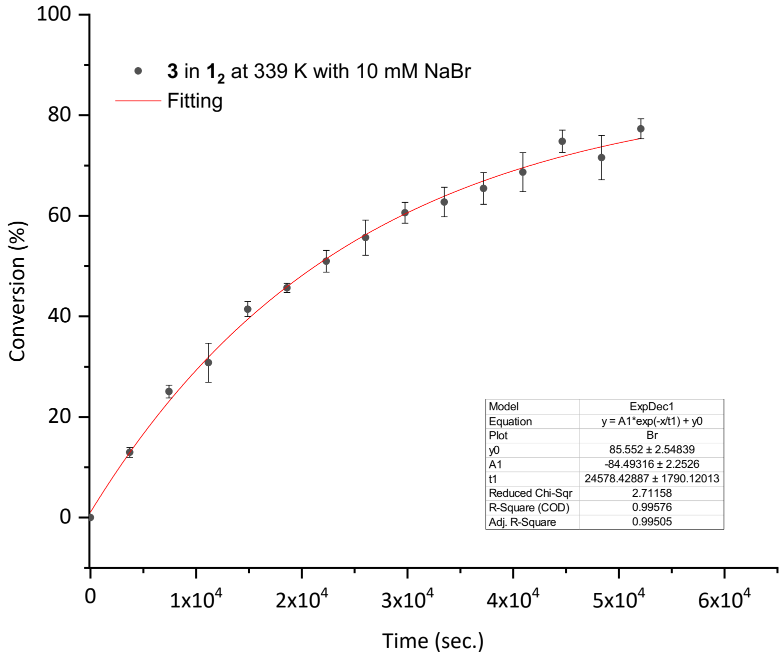


**Figure S43:** Conversion as a function of time plot for the reaction of guest **3** in **1**$_2$ in the presence of 10 equiv. NaBr at 339 K.

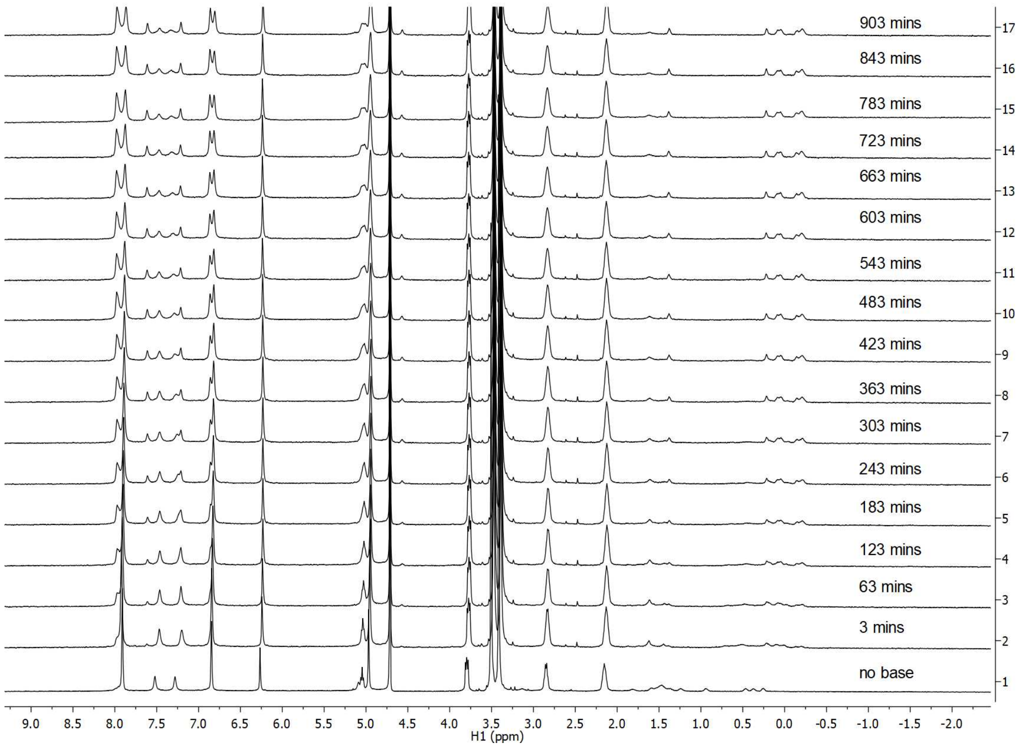


**Figure S44:** Stack of $^{1}$H NMR spectra showing reaction of encapsulated guest **3** inside of $\mathbf{1}_2$ with 10 equiv. $NaClO_4$ as a function of time. ($D_2O$, 339 K, [Host **1**] = 1 mM, [Guest **3**] = 0.5 mM, [NaOD] =10 mM).

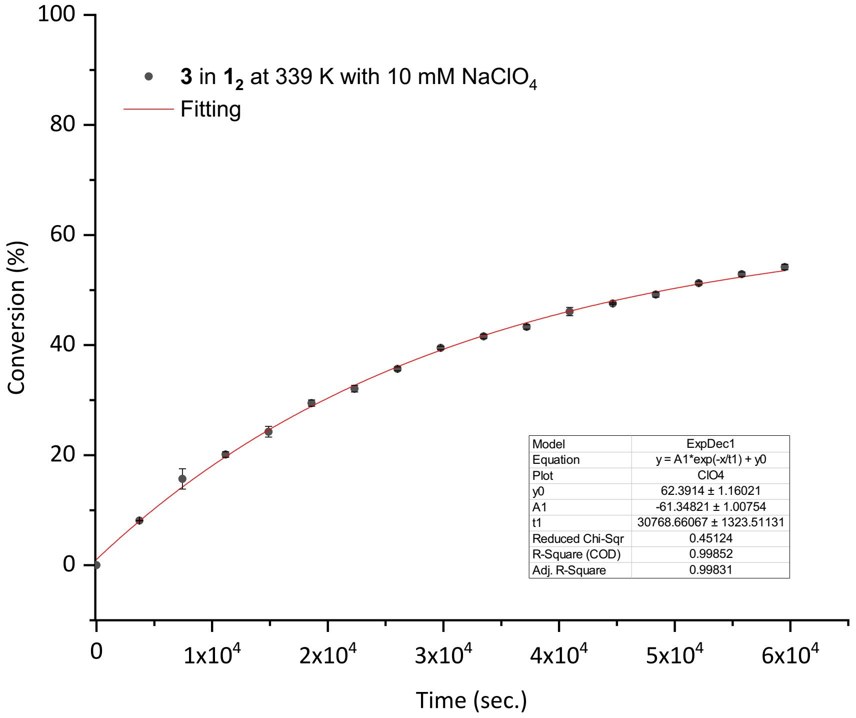


**Figure S45:** Conversion as a function of time plot for the reaction of guest **3** in $\mathbf{1}_2$ in the presence of 10 equiv. $NaClO_4$ at 339 K.

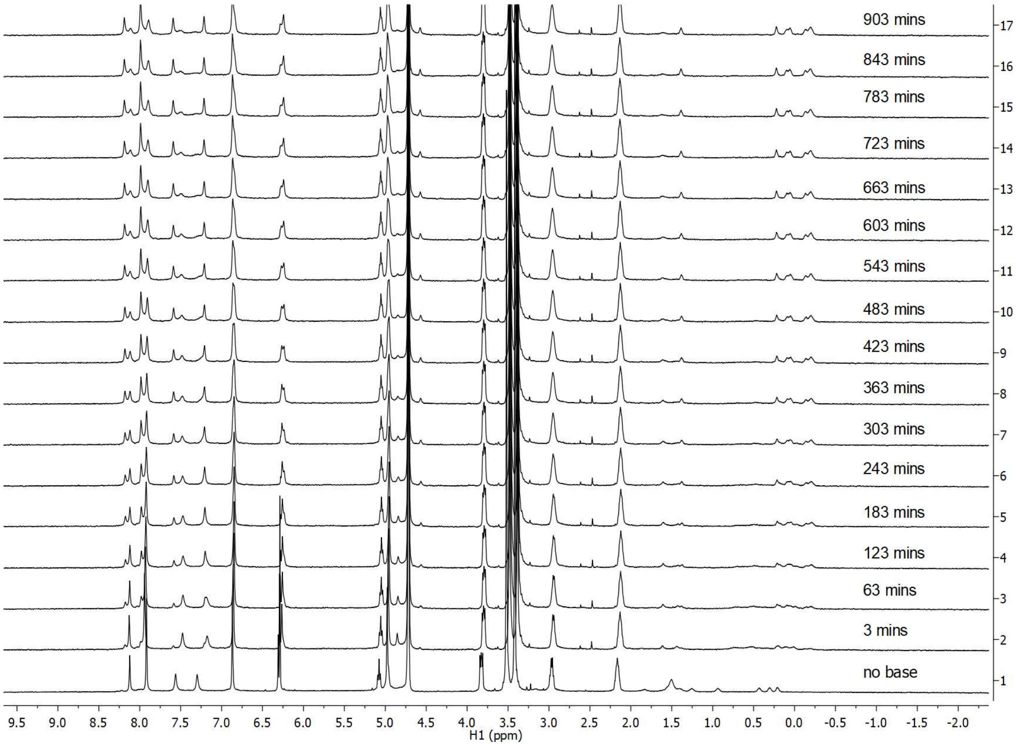


**Figure S46:** Stack of $^{1}$H NMR spectra showing reaction of encapsulated guest **3** inside of $\mathbf{1}_2$ with 10 equiv. NaDCA as a function of time. ($D_2O$, 339 K, [Host **1**] = 1 mM, [Guest **3**] = 0.5 mM, [NaOD] =10 mM).

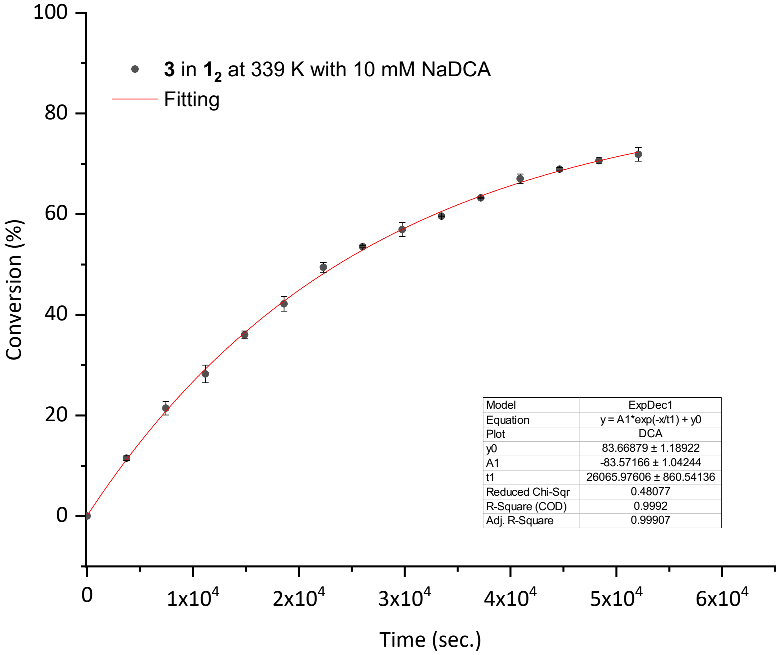


**Figure S47:** Conversion as a function of time plot for the reaction of guest **3** in $\mathbf{1}_2$ in the presence of 10 equiv. NaDCA at 339 K.

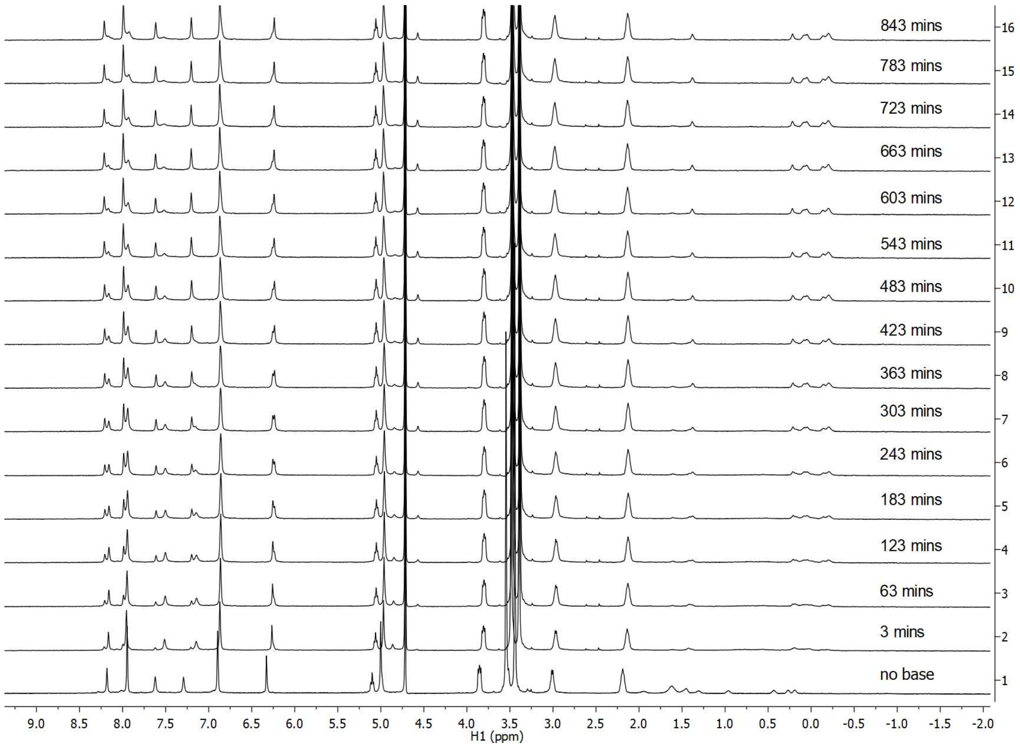


**Figure S48:** Stack of $^{1}$H NMR spectra showing reaction of encapsulated guest **3** inside of **1**$_2$ with 10 equiv. NaF as a function of time. ($D_2O$, 339 K, [Host **1**] = 1 mM, [Guest **3**] = 0.5 mM, [NaOD] =10 mM).

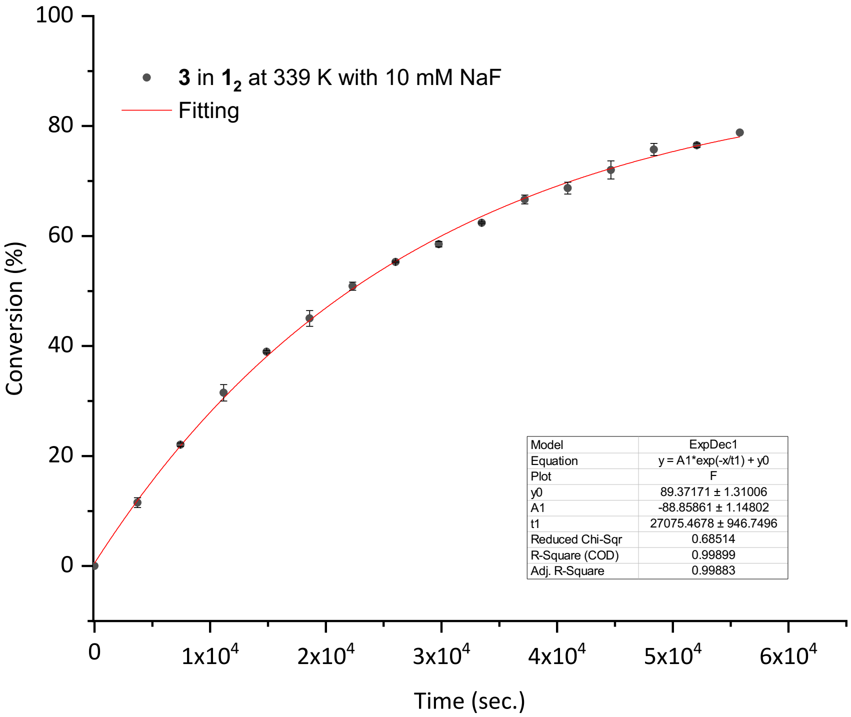


**Figure S49:** Conversion as a function of time plot for the reaction of guest **3** in **1**$_2$ in the presence of 10 equiv. NaF at 339 K.

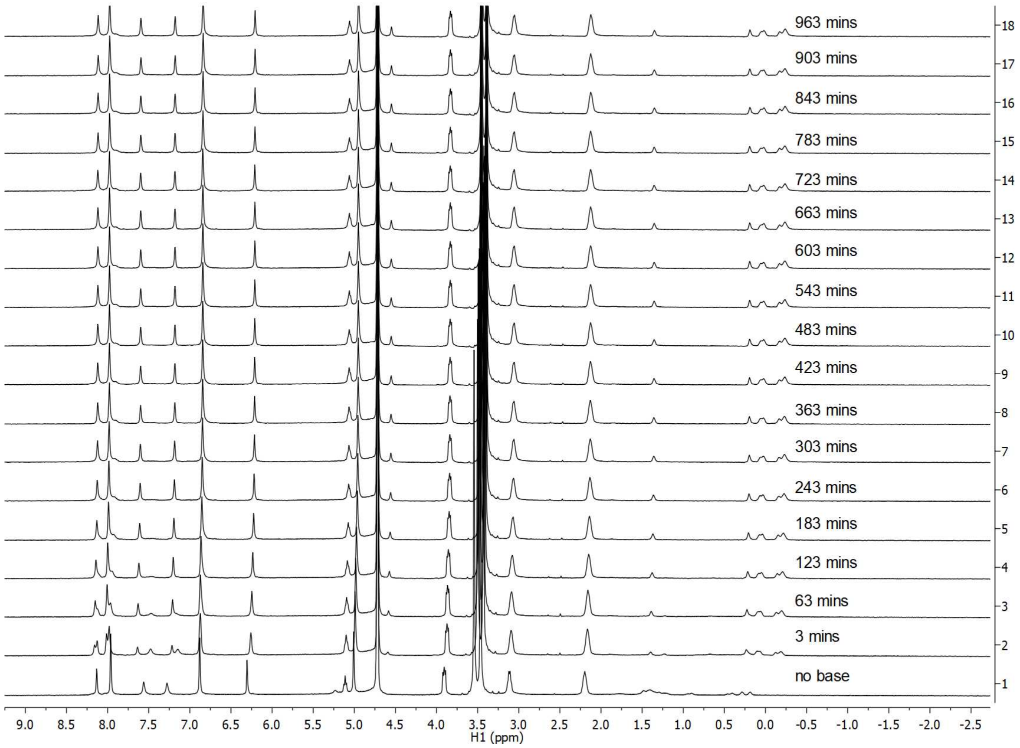


**Figure S50:** Stack of $^{1}$H NMR spectra showing reaction of encapsulated guest **3** inside of $\mathbf{1}_2$ with 10 equiv. NaI as a function of time. ($D_2O$, 339 K, [Host **1**] = 1 mM, [Guest **3**] = 0.5 mM, [NaOD] =10 mM).

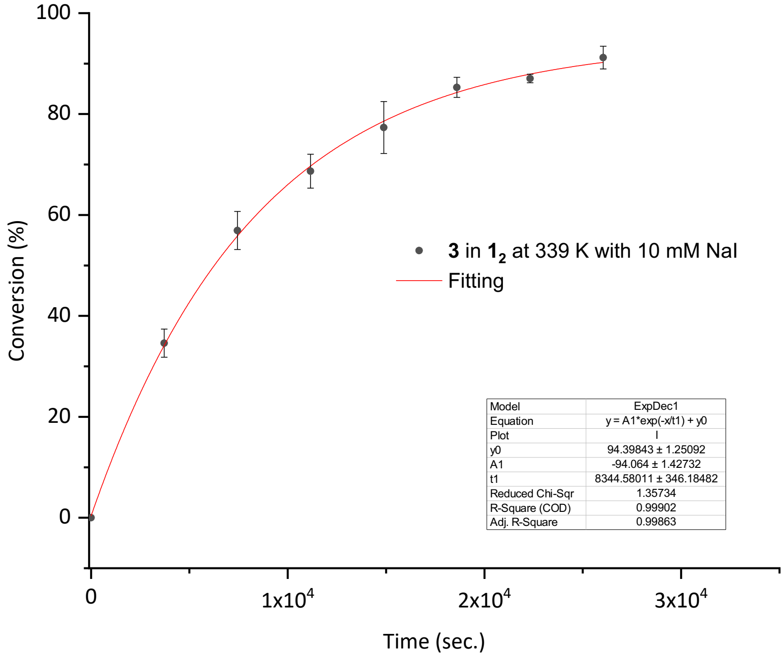


**Figure S51:** Conversion as a function of time plot for the reaction of guest **3** in $\mathbf{1}_2$ in the presence of 10 equiv. NaI at 339 K.

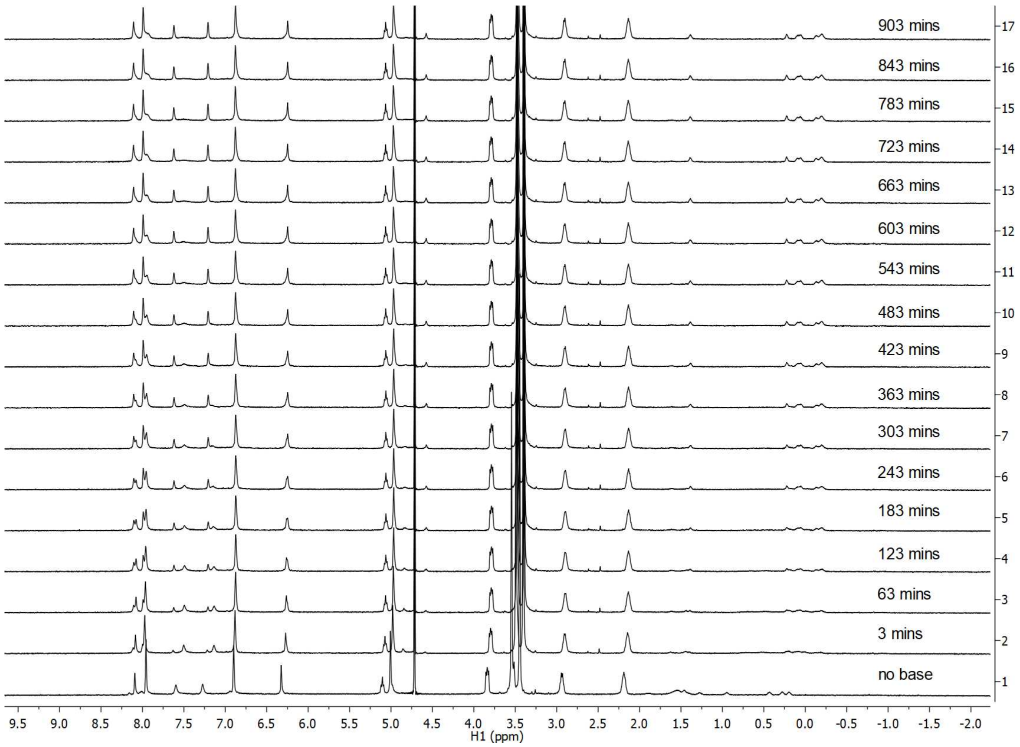


**Figure S52:** Stack of $^{1}$H NMR spectra showing reaction of encapsulated guest **3** inside of $\mathbf{1}_2$ with 10 equiv. $NaNO_3$ as a function of time. ($D_2O$, 339 K, [Host **1**] = 1 mM, [Guest **3**] = 0.5 mM, [NaOD] =10 mM).

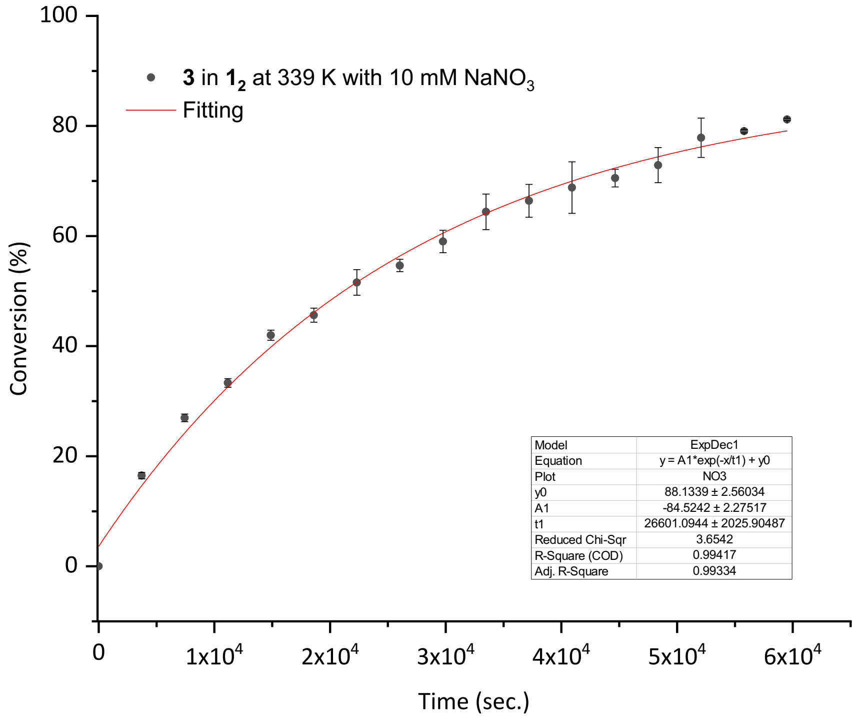


**Figure S53:** Conversion as a function of time plot for the reaction of guest **3** in $\mathbf{1}_2$ in the presence of 10 equiv. $NaNO_3$ at 339 K.

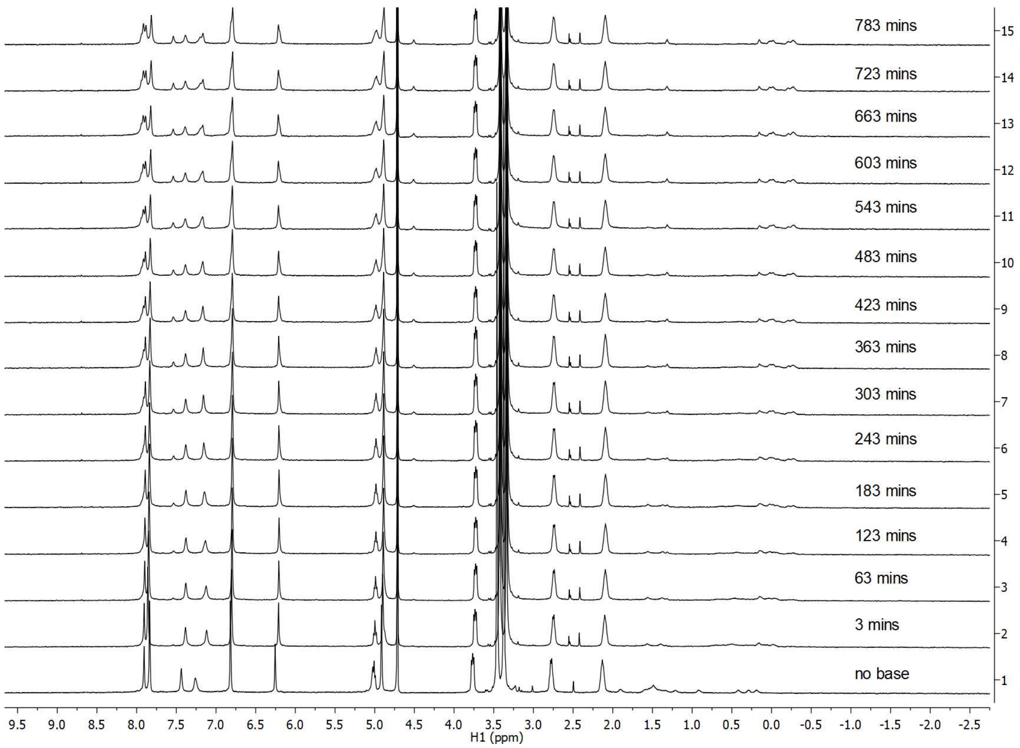


**Figure S54:** Stack of $^{1}$H NMR spectra showing reaction of encapsulated guest **3** inside of $\mathbf{1}_2$ with 10 equiv. $NaReO_4$ as a function of time. ($D_2O$, 339 K, [Host **1**] = 1 mM, [Guest **3**] = 0.5 mM, [NaOD] =10 mM).

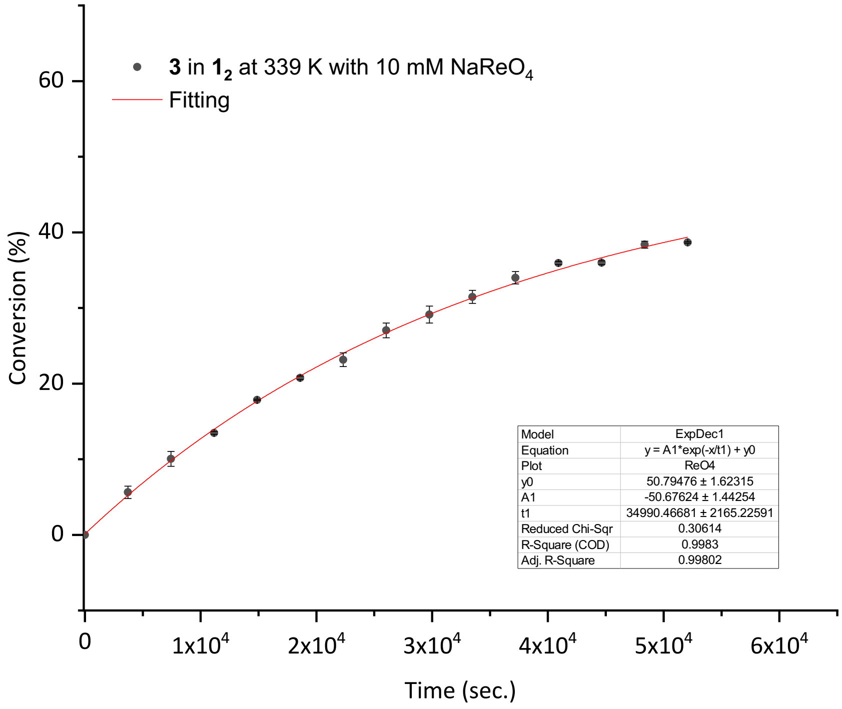


**Figure S55:** Conversion as a function of time plot for the reaction of guest **3** in $\mathbf{1}_2$ in the presence of 10 equiv. $NaReO_4$ at 339 K.

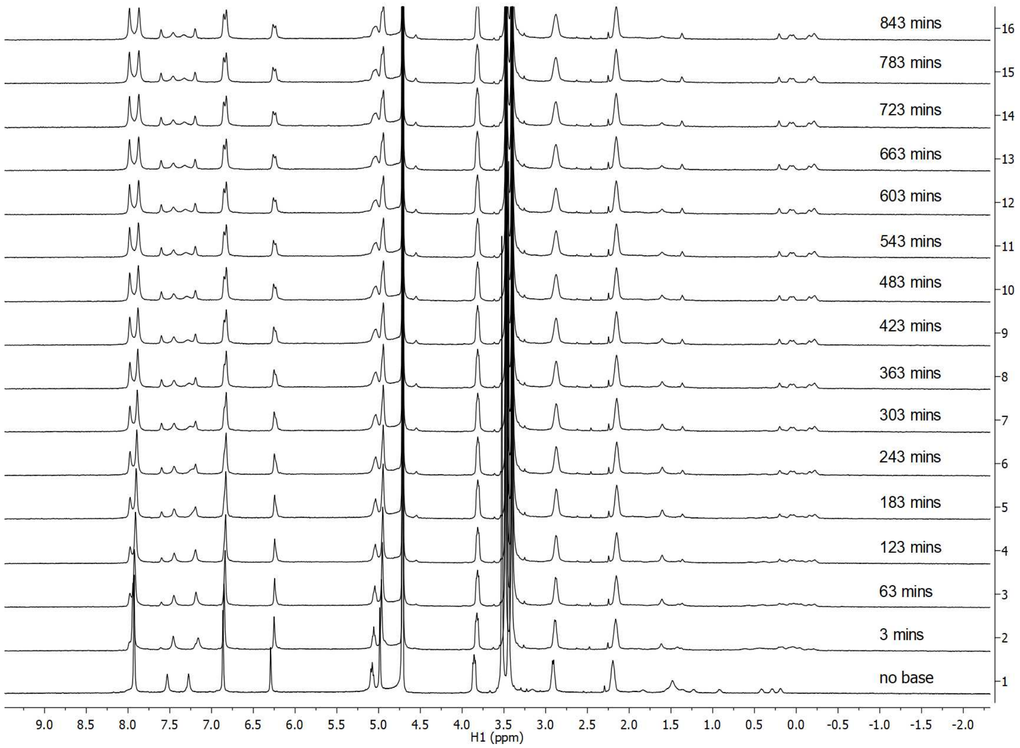


**Figure S56:** Stack of $^{1}$H NMR spectra showing reaction of encapsulated guest **3** inside of $\mathbf{1}_2$ with 10 equiv. NaSCN as a function of time. ($D_2O$, 339 K, [Host **1**] = 1 mM, [Guest **3**] = 0.5 mM, [NaOD] =10 mM).

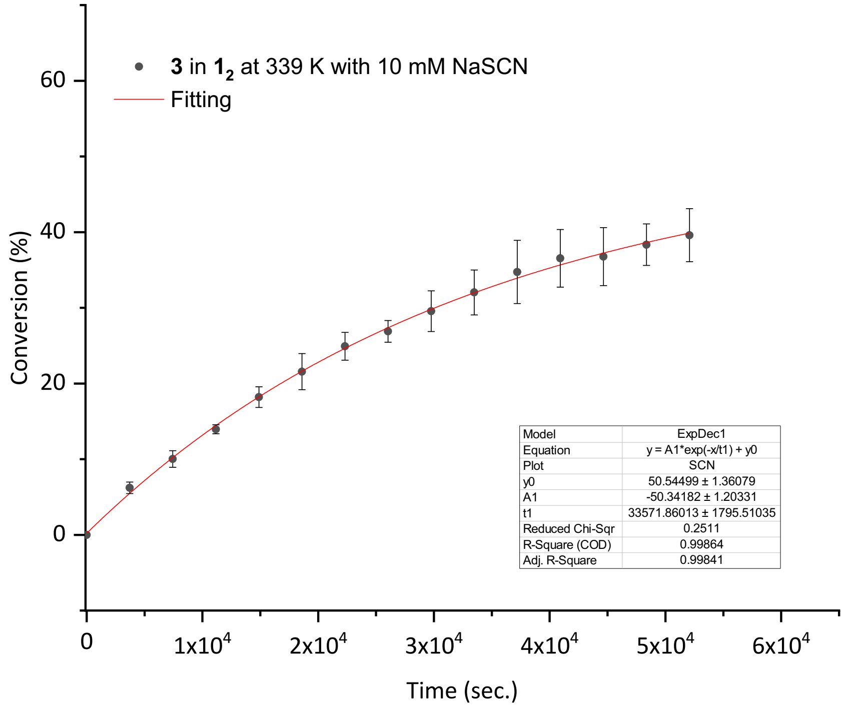


**Figure S57** Conversion as a function of time plot for the reaction of guest **3** in $\mathbf{1}_2$ in the presence of 10 equiv. NaSCN at 339 K.

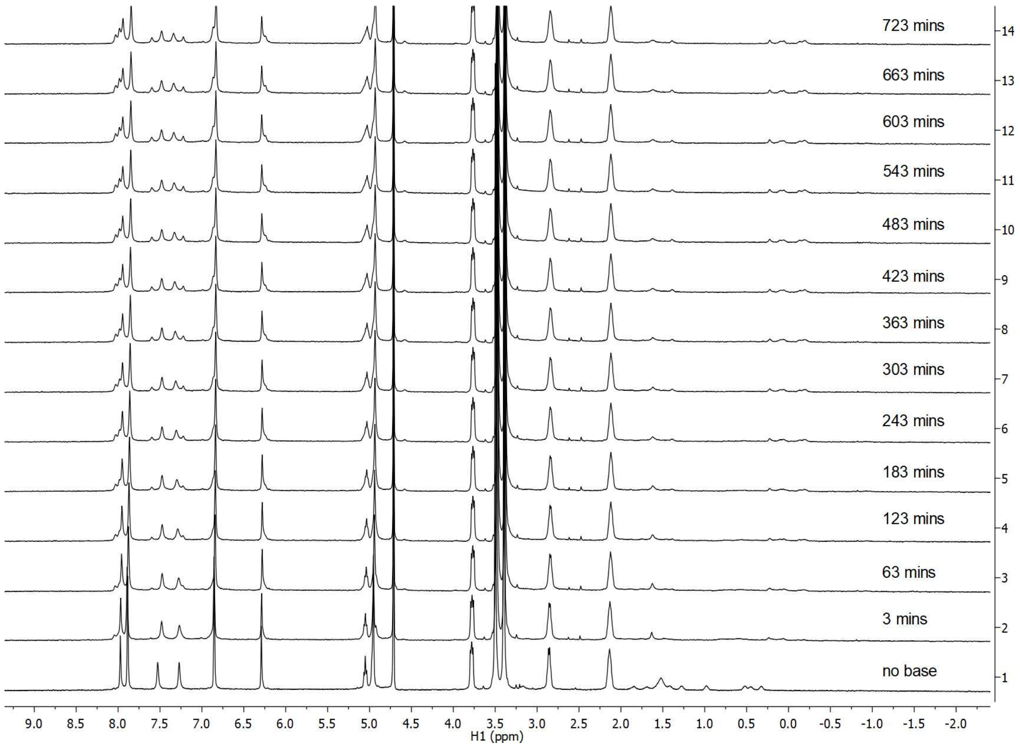


**Figure S58** Stack of $^{1}$H NMR spectra showing reaction of encapsulated guest **3** inside of $\mathbf{1}_2$ with 10 equiv. NaOTf as a function of time. ($D_2O$, 339 K, [Host **1**] = 1 mM, [Guest **3**] = 0.5 mM, [NaOD] =10 mM).

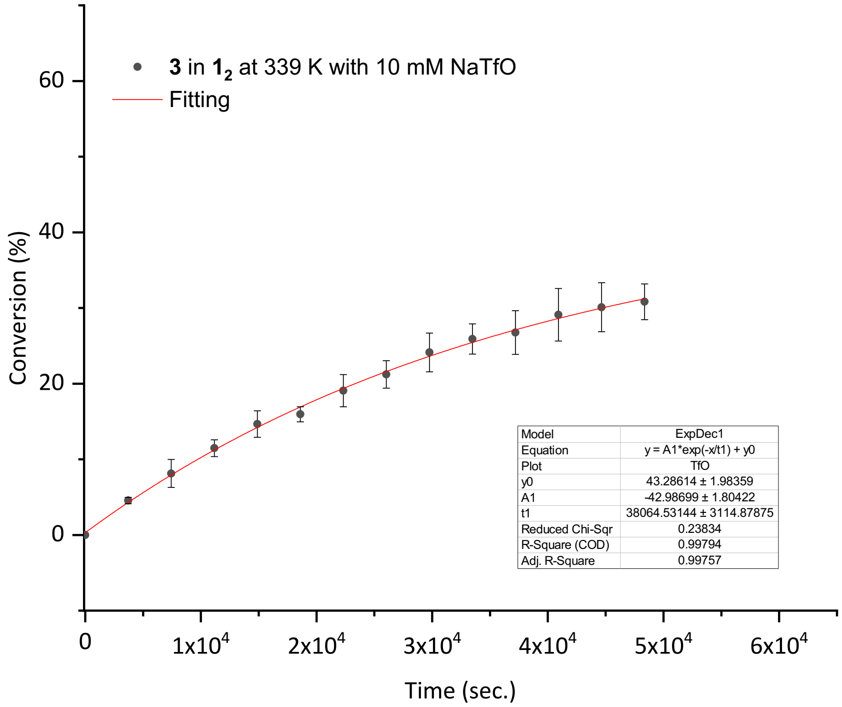


**Figure S59:** Conversion as a function of time plot for the reaction of guest **3** in $\mathbf{1}_2$ in the presence of 10 equiv. NaOTf at 339 K.

## 6) Cyclization of 3 within $2_2$ in the presence of various salts

We also studied how the presence of cations impacted the cyclization rate of **3** within the capsule formed by **2**. Six cations were selected: $Na^+$, $Li^+$, $K^+$, $Rb^+$, $Cs^+$ and tetramethylammonium ($TMA^+$). **Figures S60** – **S76** show the corresponding data for reactions carried out with the same protocol but in the presence of 10 mM of the cations (as their chloride salts). The experiments were performed with 0.5 mL samples of 1.0 mM host **2** in $D_2O$ at 336 K. In each case, a small volume of 400 mM salt solution was added to make a 10 mM salt solution of the complex.

## 6.1) Cation effect: Summary of Data

Figures S60 and S61 show the $^1$H NMR spectra of the complex with $\mathbf{2}_2$ in the presence of 10 mM salt.

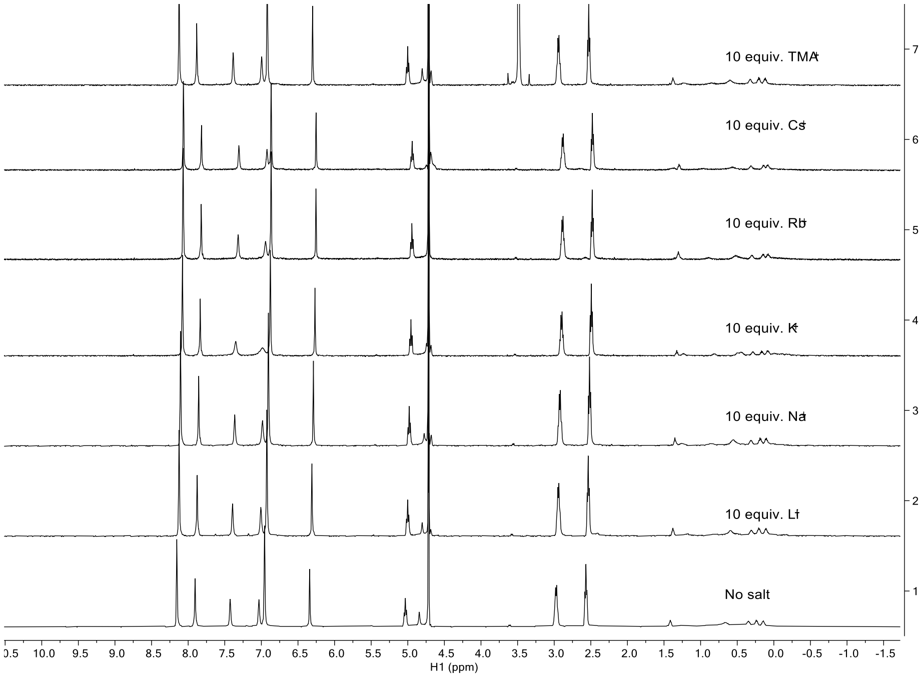


**Figure S60:** Stack of $^1$H NMR spectra of **3** in $\mathbf{2}_2$ in the presence of various of cation at 336 K

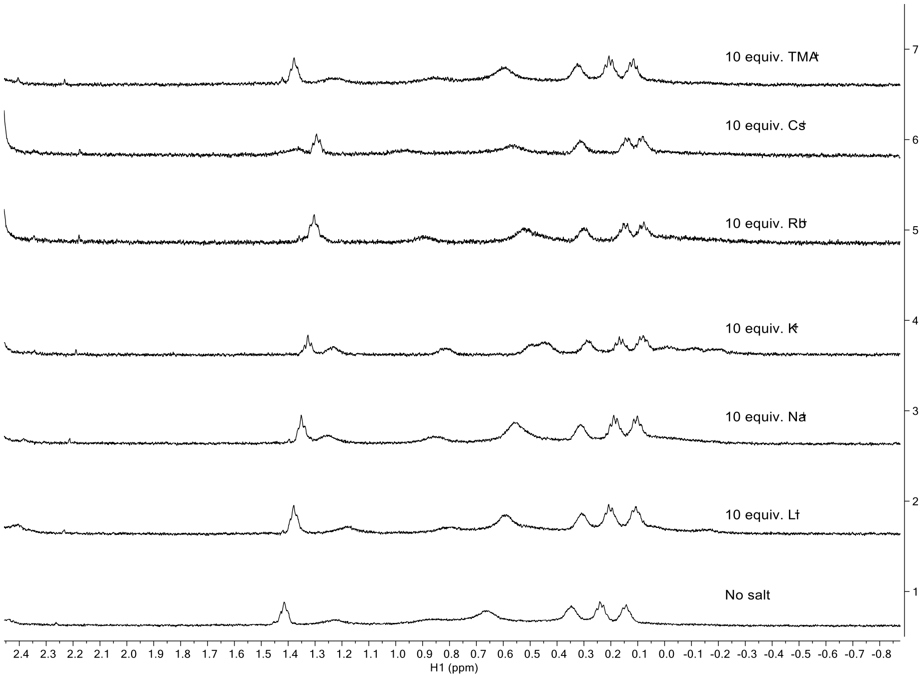


**Figure S61:** Stack of $^1$H NMR spectra of **3** in $\mathbf{2}_2$ guest region in the presence of various of cation at 336 K

The extent of reaction of each experiment was determined by tracking and integrating the $^{1}H$ NMR signal from the $H_d$ proton (Mnova), and fitting this data as a function of time to give the rate constant data (Origin Pro 2018. Figure S39 shows the cumulative data for all the salts. Each experiment was carried out in duplicate (error bars shown), with the average calculated used to give the rate constant (Table 3). Errors were <10%.

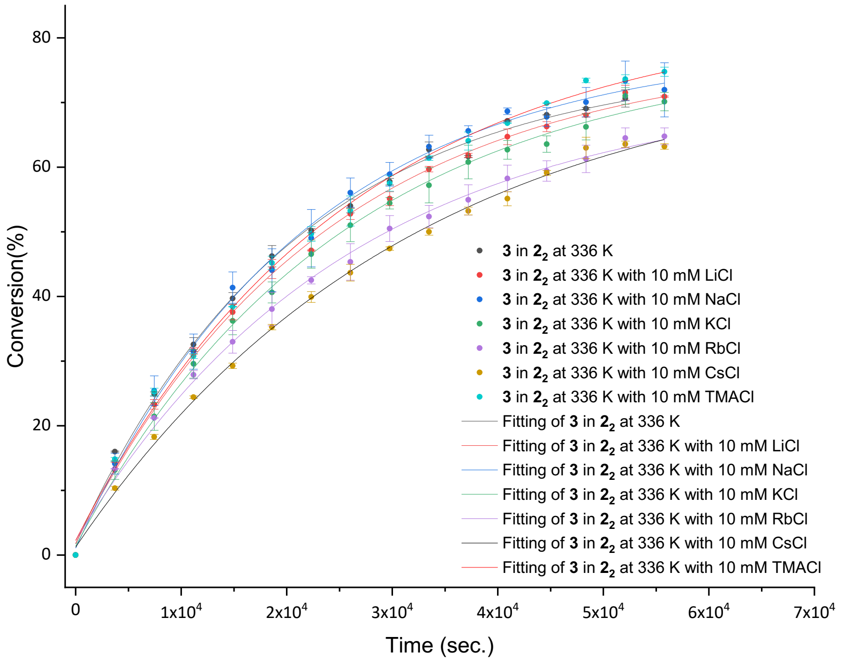


| Model | ExpDec1 | | | | | | |
|---|---|---|---|---|---|---|---|
| Equatio | y = A1*exp(-x/t1) + y0 | | | | | | |
| Plot | OA | Li | Na | K | Rb | Cs | TMA |
| y0 | 76.36757 ± | 77.98387 ± | 78.95909 ± | 78.29099 ± | 72.35902 ± | 77.37557 ± | 83.90897 ± |
| A1 | -74.63319 ± | -76.18507 | -77.8953 ± | -76.87905 ± | -70.08684 ± | -76.13594 ± | -81.6566 ± |
| **t1** | **2.08E+04 ±** | **2.35E+04 ±** | **2.17E+04 ±** | **2.52E+04 ±** | **2.58E+04 ±** | **3.17E+04 ±** | **2.55E+04 ±** |
| Reduce | 1.54159 | 1.17051 | 1.92154 | 1.1805 | 1.34079 | 1.04827 | 1.58362 |
| R-Squa | 0.99713 | 0.9978 | 0.99666 | 0.99775 | 0.9969 | 0.99768 | 0.99732 |
| Adj. R- | 0.99665 | 0.99747 | 0.99614 | 0.99741 | 0.99642 | 0.99732 | 0.9969 |

**Figure S62:** Overall conversions as a function of time for the reaction of guest **3** in $\mathbf{2}_2$ in the presence of 10 equiv. different cation at 336 K (*top*), and summary of kinetic parameters from fitting to Eq. 2 (*bottom*).

**Table 3.** The reaction rate (*k*) of **3** in **2** in the presence of different cations at 336 K.

| Salt | Rate Constant, *k* in $10^{-5}$ $s^{-1}$ |
|---|---|
| No salt | 4.81 |
| $Na^{+}$ | 4.61 |
| $Li^{+}$ | 4.26 |
| $K^{+}$ | 3.97 |
| $TMA^{+}$ | 3.92 |
| $Rb^{+}$ | 3.88 |
| $Cs^{+}$ | 3.15 |

## 6.2) Cation effect: Individual data

The reaction rate in each experiment was determined by tracking and integrating the “d” peak in the $^{1}H$ NMR spectroscopy using Mnova, then fitted the integrations as a function of time to give the kinetic data with Origin Pro 2018. Lines fitted by the first order kinetic model are demonstrated in red and the standard deviations are shown as error bars. Each experiment was carried out in duplicates, with the average calculated to give the corresponding rate constants. Errors were <10%.

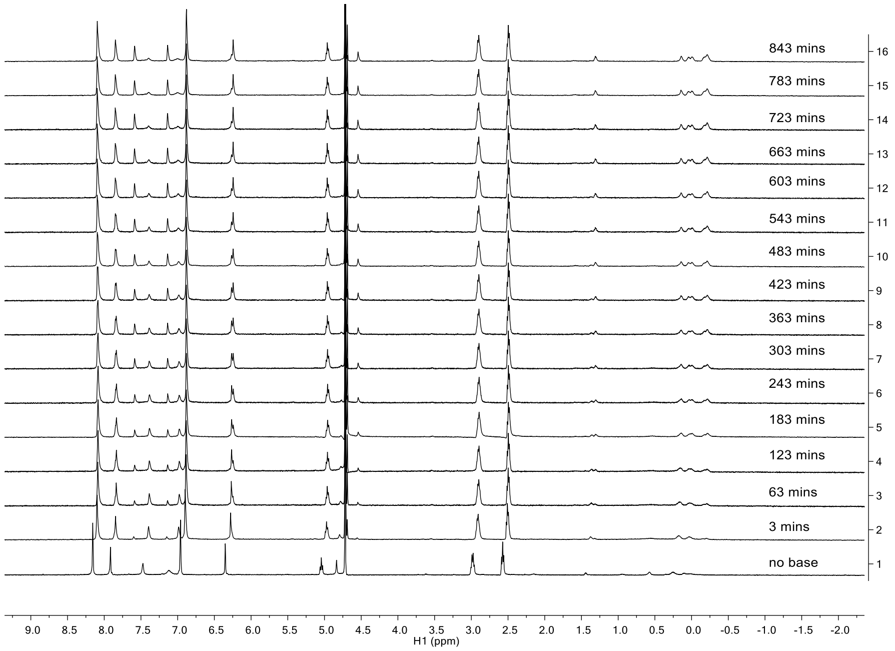


**Figure S63:** Stack of $^{1}H$ NMR spectra showing reaction of encapsulated guest **3** inside of $\mathbf{2}_2$ as a function of time. ($D_2O$, 336 K, [Host **2**] = 1 mM, [Guest **3**] = 0.5 mM, [NaOD] =10 mM).

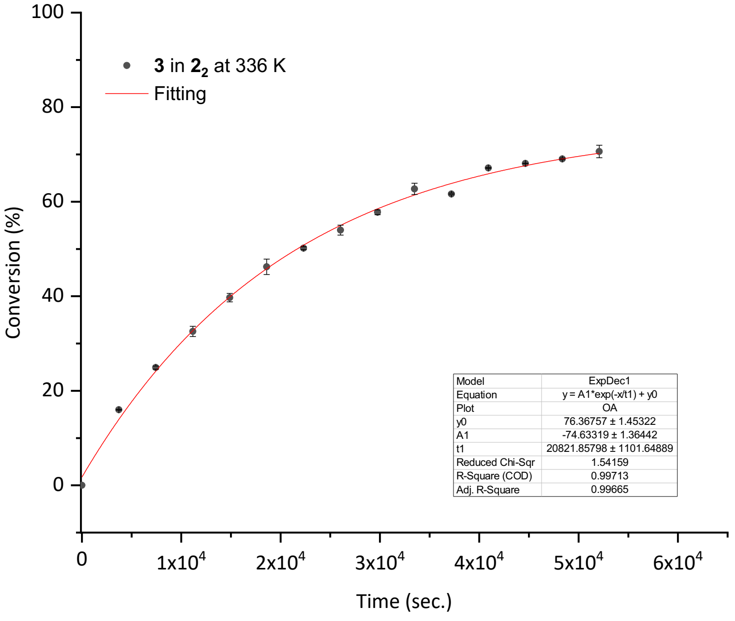


**Figure S64:** Conversion as a function of time plot for the reaction of guest **3** in $\mathbf{2}_2$ at 336 K.

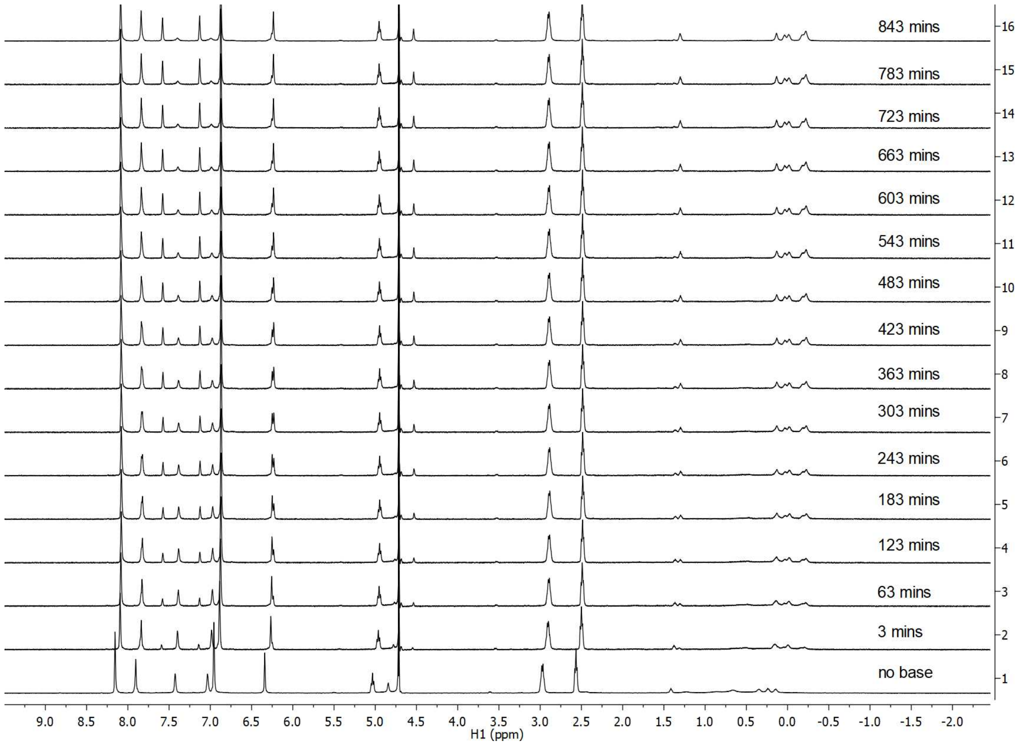


**Figure S65:** Stack of $^{1}$H NMR spectra showing reaction of encapsulated guest **3** inside of **2**$_2$ with 10 equiv. LiCl as a function of time. ($D_2O$, 336 K, [Host **2**] = 1 mM, [Guest **3**] = 0.5 mM, [NaOD] =10 mM).

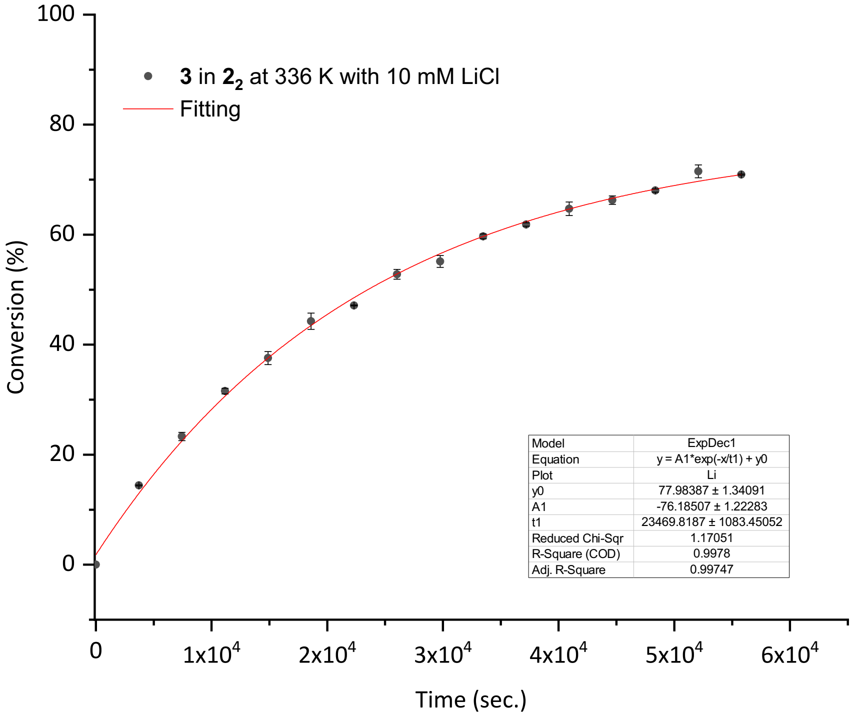


**Figure S66:** Conversion as a function of time plot for the reaction of guest **3** in **2**$_2$ in the presence of 10 equiv. LiCl at 336 K.

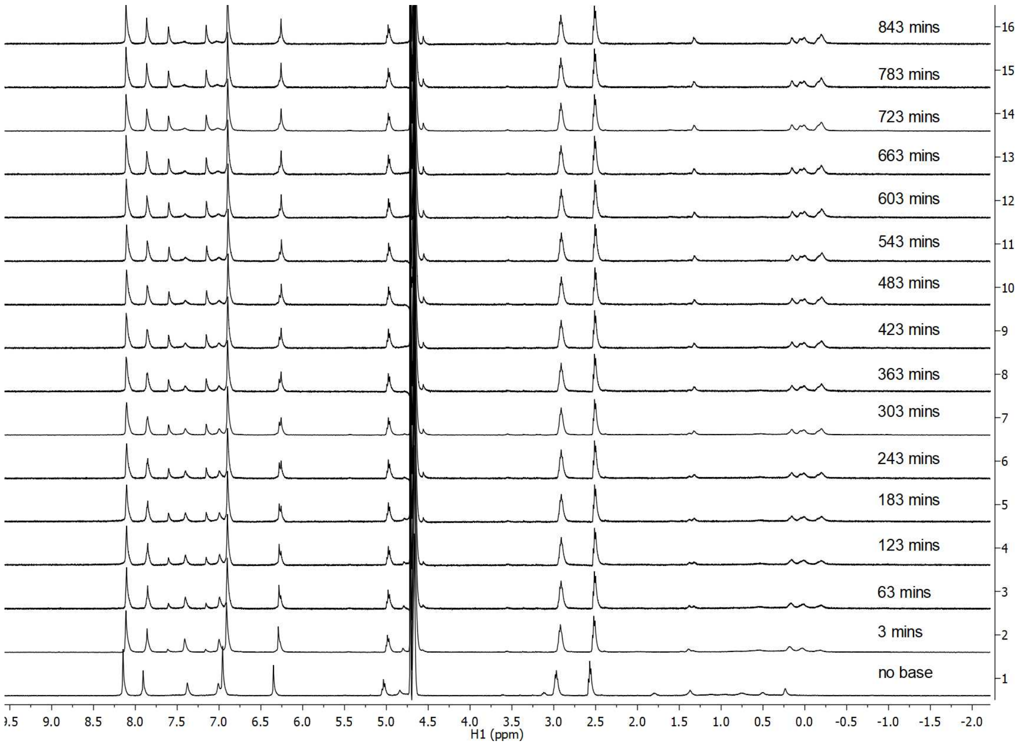


**Figure S67:** Stack of $^{1}$H NMR spectra showing reaction of encapsulated guest **3** inside of $\mathbf{2}_2$ with 10 equiv. NaCl as a function of time. ($D_2O$, 336 K, [Host **2**] = 1 mM, [Guest **3**] = 0.5 mM, [NaOD] =10 mM).

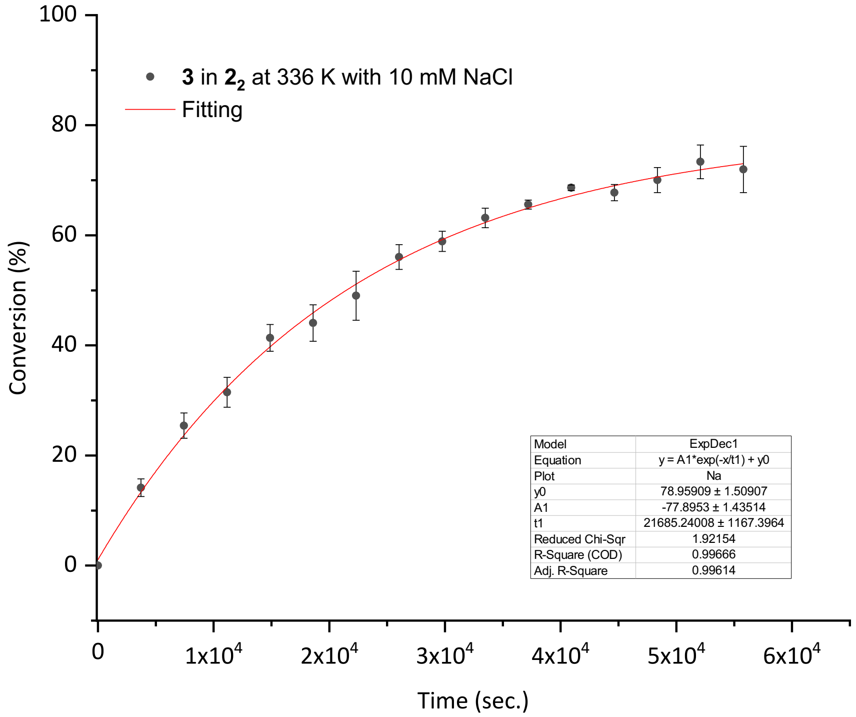


**Figure S68:** Conversion as a function of time plot for the reaction of guest **3** in $\mathbf{2}_2$ in the presence of 10 equiv. NaCl at 336 K.

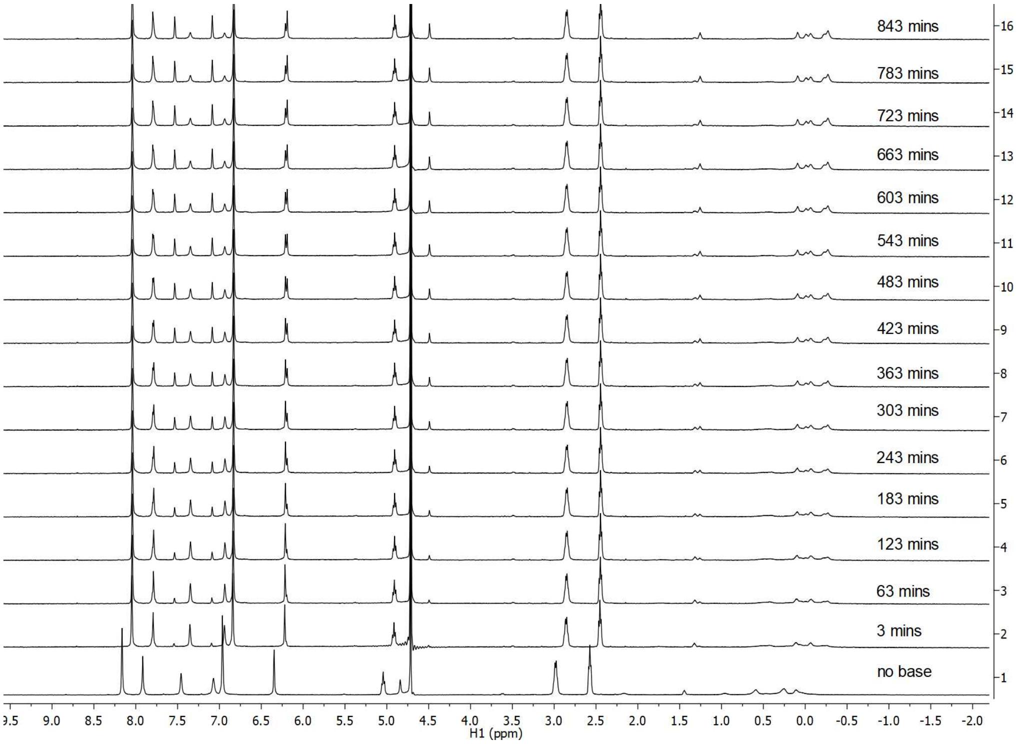


**Figure S69:** Stack of $^{1}$H NMR spectra showing reaction of encapsulated guest **3** inside of $\mathbf{2}_2$ with 10 equiv. KCl as a function of time. ($D_2O$, 336 K, [Host **2**] = 1 mM, [Guest **3**] = 0.5 mM, [NaOD] =10 mM).

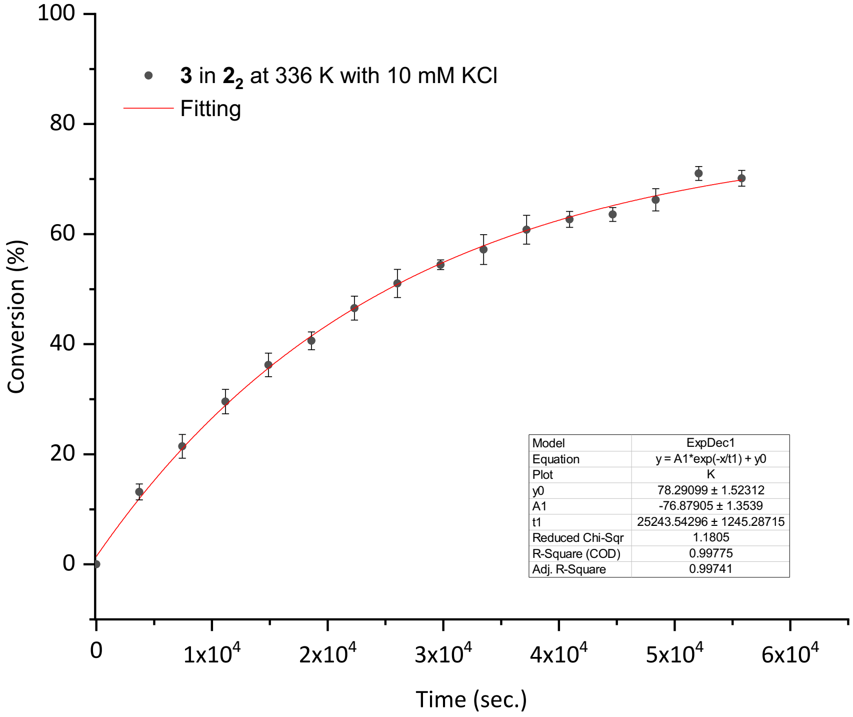


**Figure S70:** Conversion as a function of time plot for the reaction of guest **3** in $\mathbf{2}_2$ in the presence of 10 equiv. KCl at 336 K.

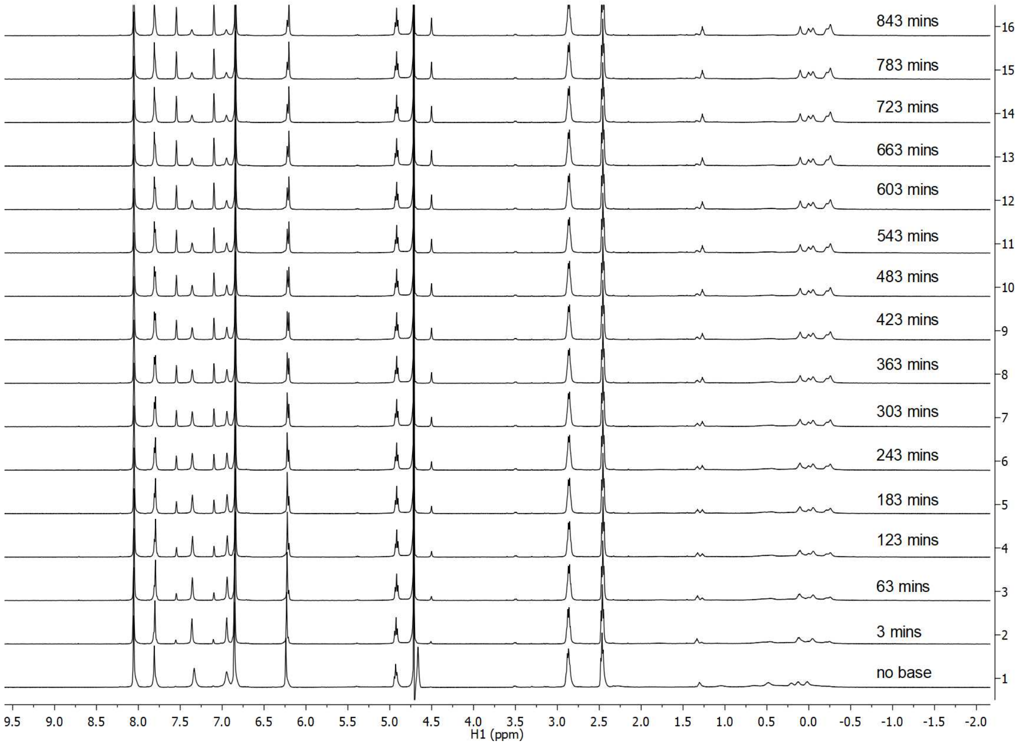


**Figure S71:** Stack of $^{1}$H NMR spectra showing reaction of encapsulated guest **3** inside of $\mathbf{2}_2$ with 10 equiv. CsCl as a function of time. ($D_2O$, 336 K, [Host **2**] = 1 mM, [Guest **3**] = 0.5 mM, [NaOD] =10 mM).

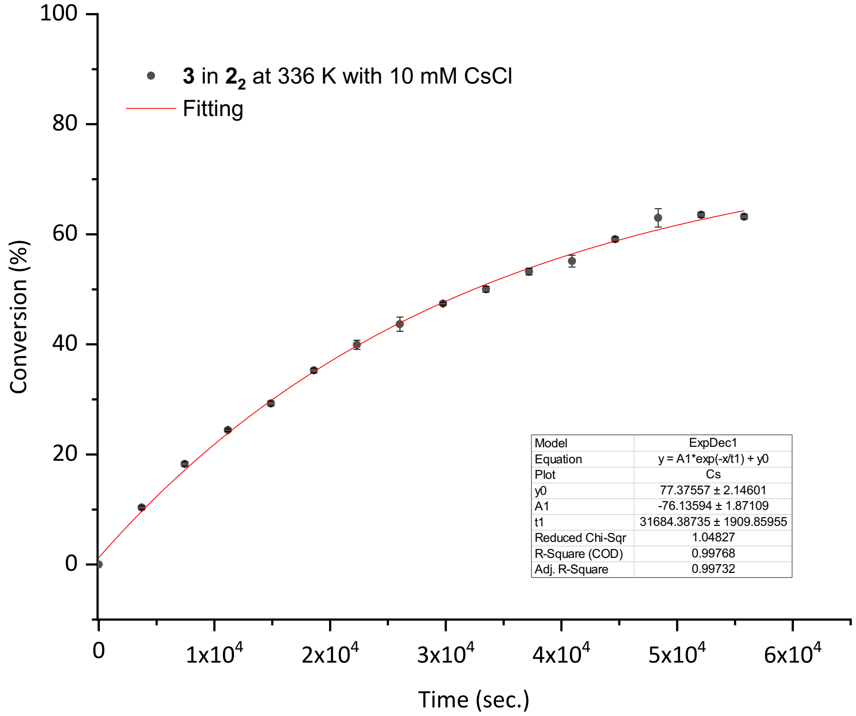


**Figure S72:** Conversion as a function of time plot for the reaction of guest **3** in $\mathbf{2}_2$ in the presence of 10 equiv. CsCl at 336 K.

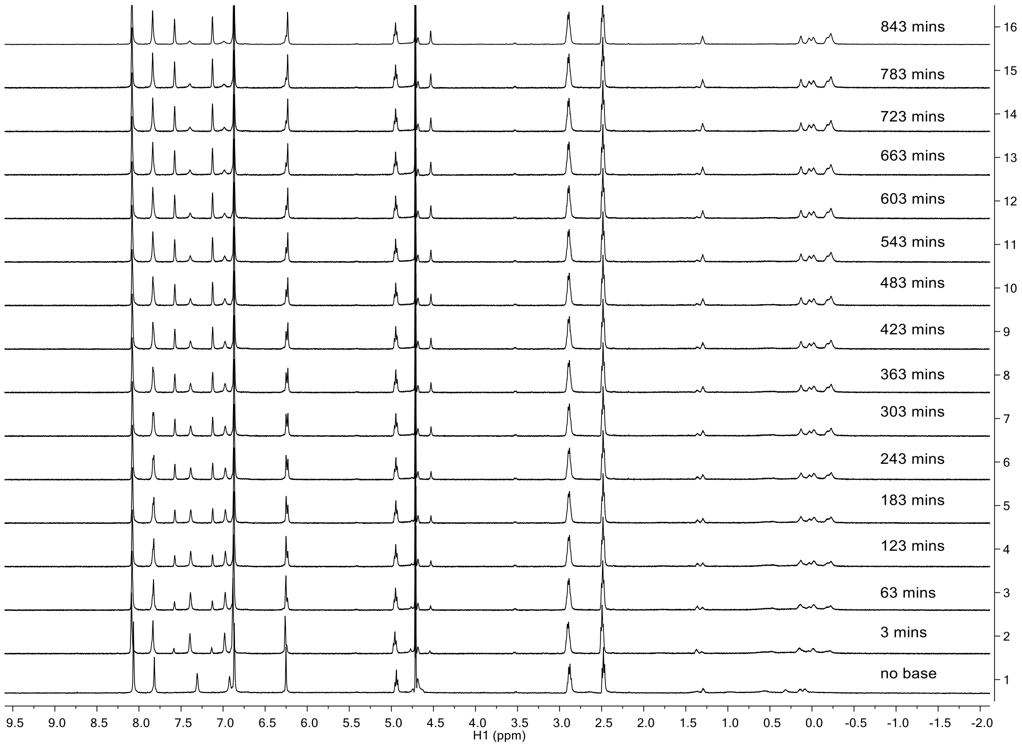


**Figure S73:** Stack of $^{1}$H NMR spectra showing reaction of encapsulated guest **3** inside of $\mathbf{2}_2$ with 10 equiv. RbCl as a function of time. ($D_2O$, 336 K, [Host **2**] = 1 mM, [Guest **3**] = 0.5 mM, [NaOD] =10 mM).

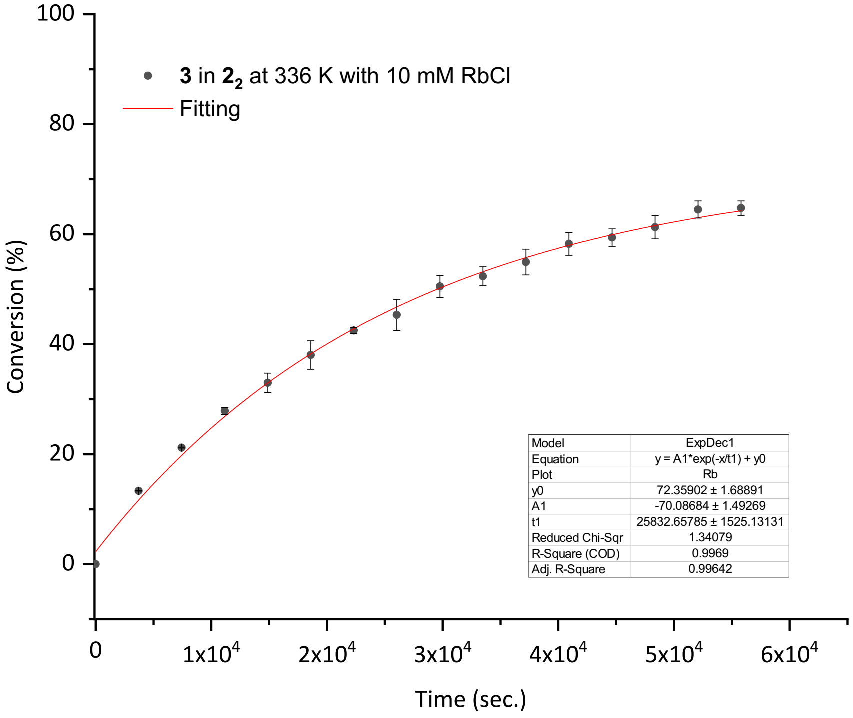


**Figure S74:** Conversion as a function of time plot for the reaction of guest **3** in $\mathbf{2}_2$ in the presence of 10 equiv. RbCl at 336 K.

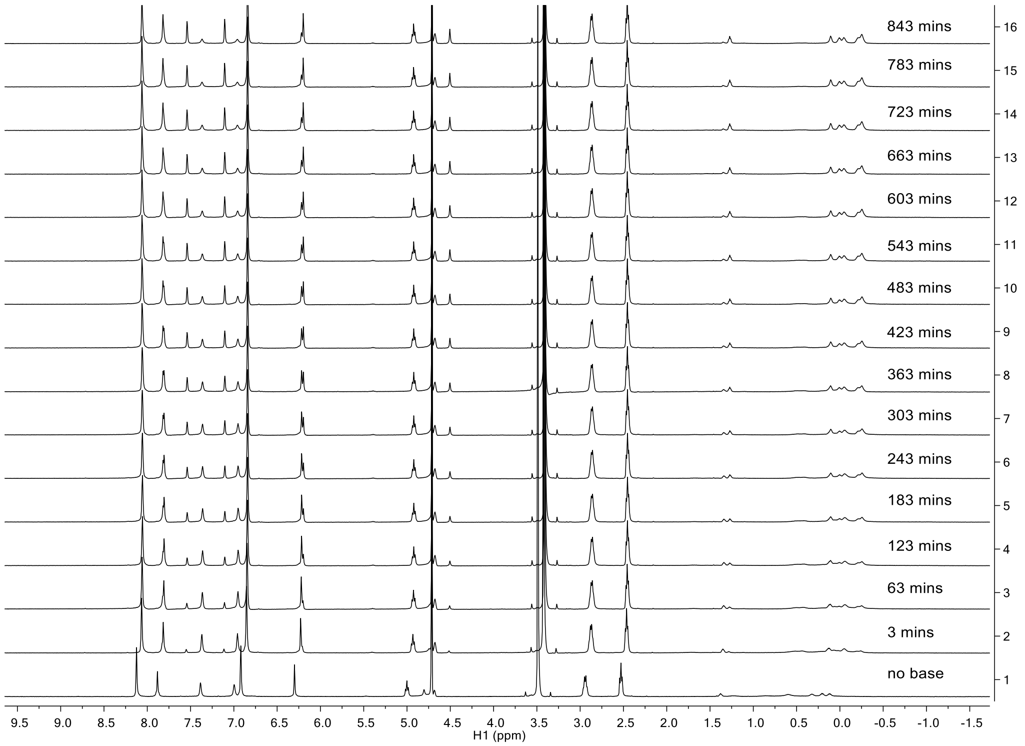


**Figure S75:** Stack of $^{1}$H NMR spectra showing reaction of encapsulated guest **3** inside of **2**$_2$ with 10 equiv. TMACl as a function of time. ($D_2O$, 336 K, [Host **2**] = 1 mM, [Guest **3**] = 0.5 mM, [NaOD] =10 mM).

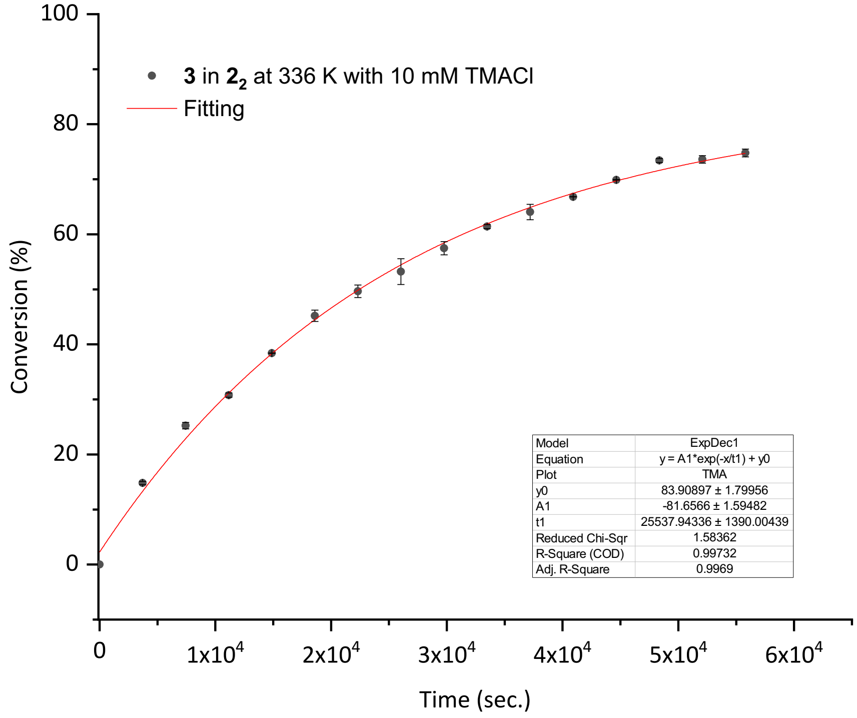


**Figure S76** Conversion as a function of time plot for the reaction of guest **3** in **2**$_2$ in the presence of 10 equiv. TMACl at 336 K.

7) Eyring analysis for the cyclization of **3** presence of 10 mM $NaClO_4$ or CsCl

The reaction rate in each experiment was determined by tracking and integrating the “d” peak in the $^1$H NMR spectroscopy using Mnova, then fitted the integrations as a function of time to give the kinetic data with Origin Pro 2018. Lines fitted by the first order kinetic model are demonstrated in red and the standard deviations are shown as error bars. Each experiment was carried out in duplicates, with the average calculated to give the corresponding rate constants. Errors in individual rate constant were <10%. Gradient error in the Eyring analysis was 5% (Excel LINEST function). **Figures S77 – S88** show the data for the cyclization of **3** in $\mathbf{1}_2$ in the presence of $NaClO_4$, whilst **Figures S89 – S100** show the data for the cyclization of **3** in $\mathbf{2}_2$ in the presence of CsCl.

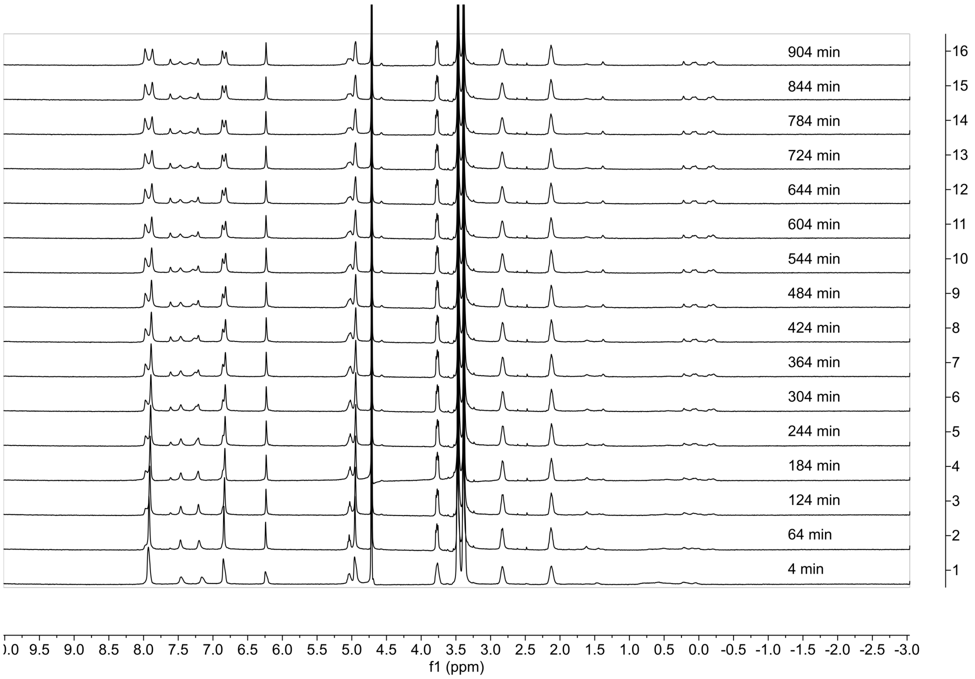


**Figure S77**: Stack of $^1$H NMR spectra showing reaction of encapsulated guest **3** inside of $\mathbf{1_2}$ as a function of time. ($D_2O$, 339 K, [Host **1**] = 1 mM, [Guest **3**] = 0.5 mM, [NaOD] =10 mM, [$NaClO_4$] = 10 mM).

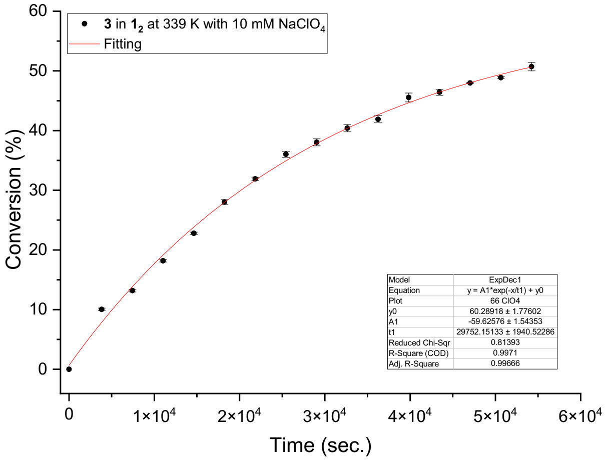


**Figure S78:** Reaction kinetics for guest **3** inside of host $\mathbf{1_2}$ in presence of 10 mM $NaClO_4$ at 339 K.

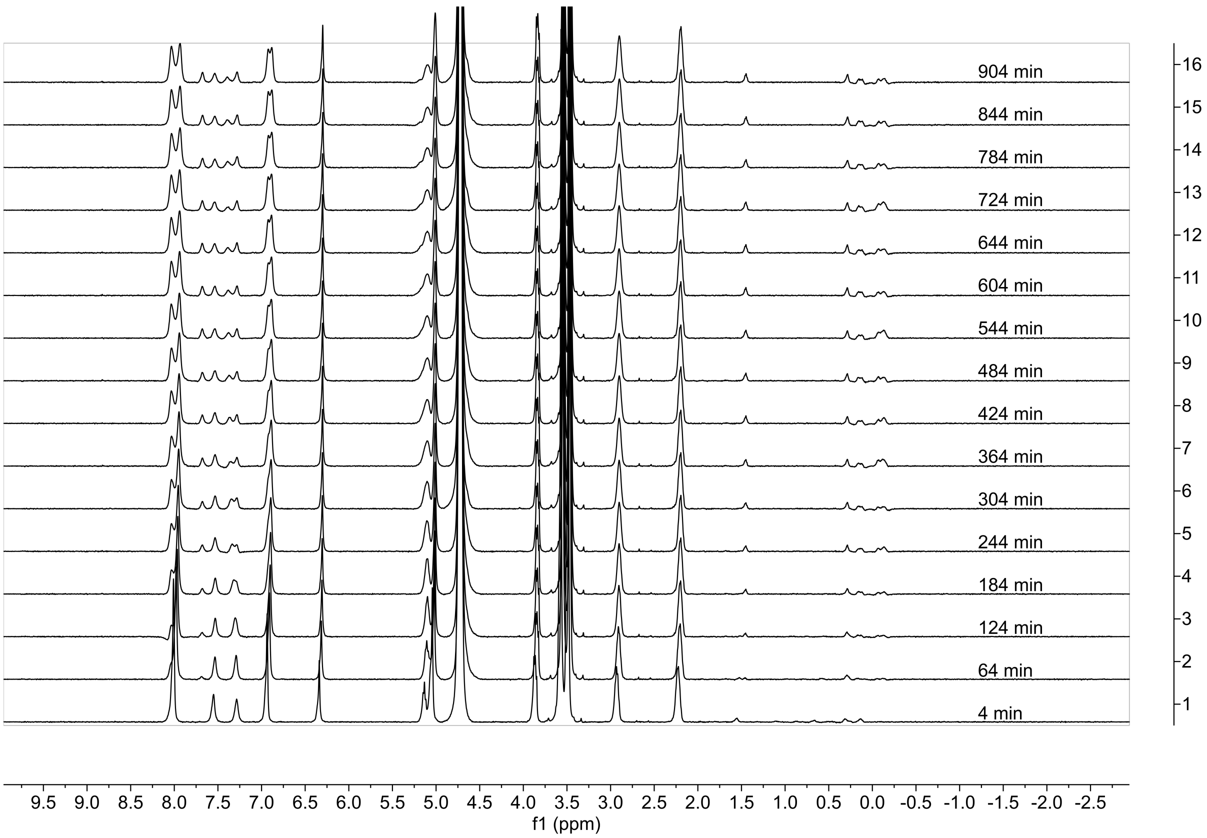


**Figure S79**: Stack of $^1$H NMR spectra showing reaction of encapsulated guest **3** inside of $\mathbf{1_2}$ as a function of time. ($D_2O$, 342 K, [Host **1**] = 1 mM, [Guest **3**] = 0.5 mM, [NaOD] =10 mM, [$NaClO_4$] = 10 mM).

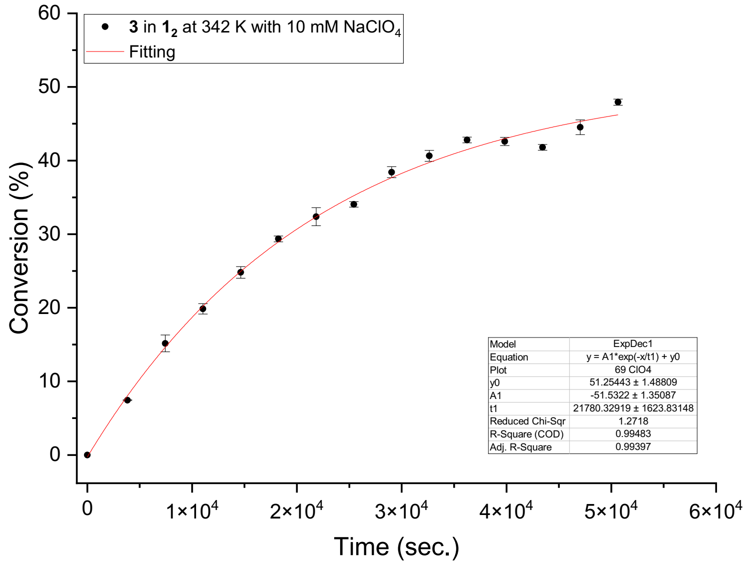


**Figure S80:** Reaction kinetics for guest **3** inside of host **1$_2$** in presence of 10 mM NaClO$_4$ at 342 K.

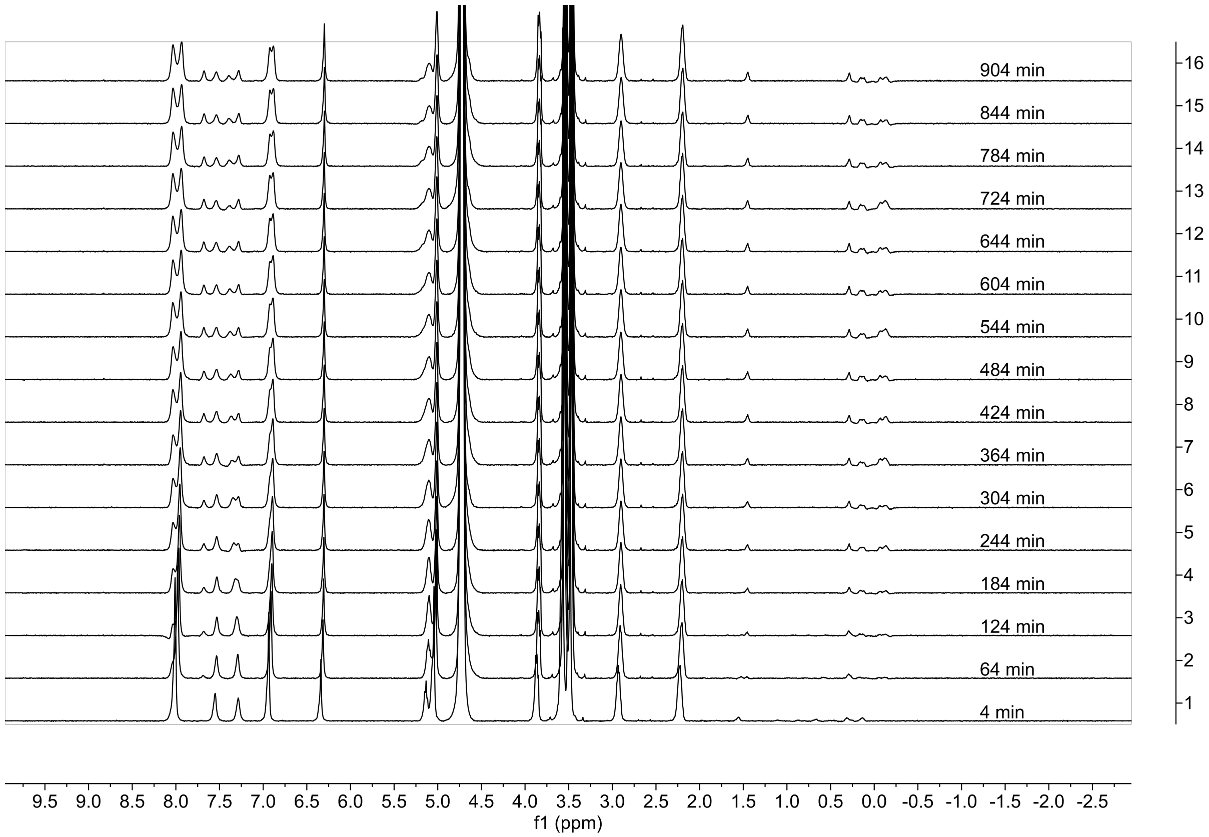


**Figure S81**: Stack of $^1$H NMR spectra showing reaction of encapsulated guest **3** inside of **1$_2$** as a function of time. ($D_2O$, 345 K, [Host **1**] = 1 mM, [Guest **3**] = 0.5 mM, [NaOD] =10 mM, [NaClO$_4$] = 10 mM).

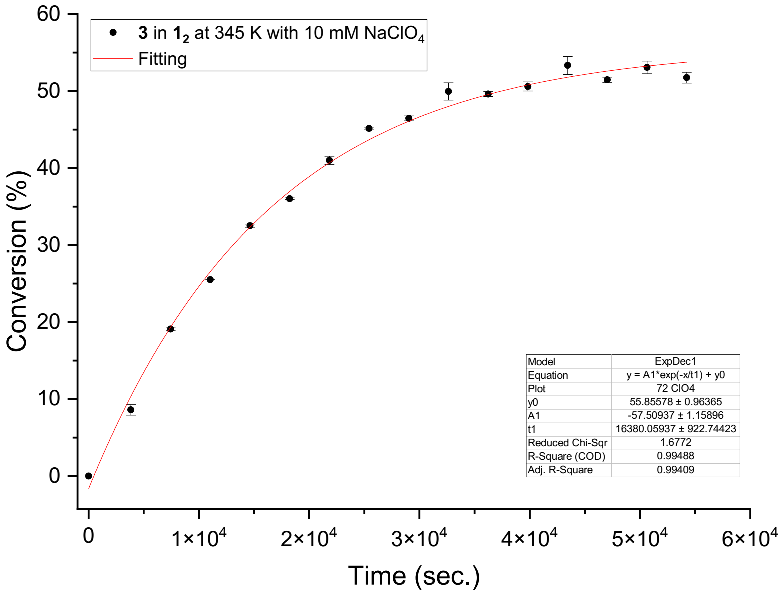


**Figure S82:** Reaction kinetics for guest **3** inside of host $\mathbf{1_2}$ in presence of 10 mM $NaClO_4$ at 345 K.

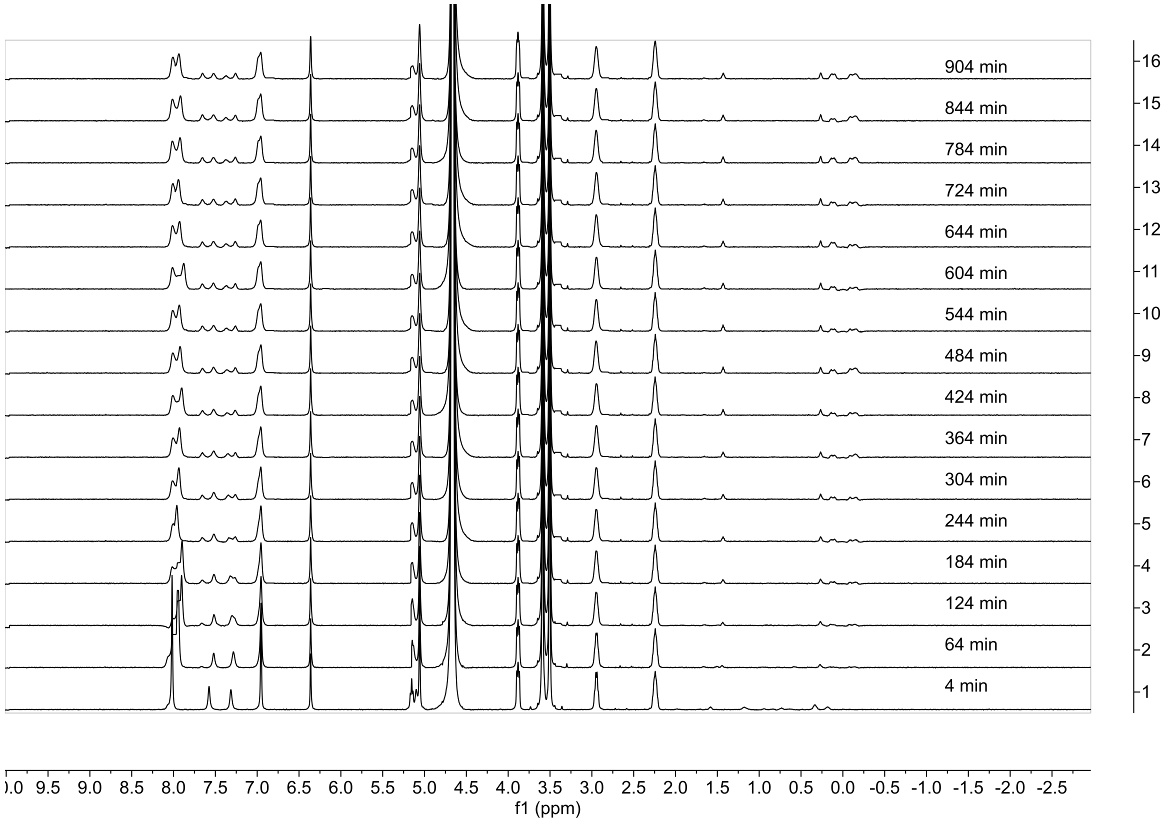


**Figure S83**: Stack of $^1$H NMR spectra showing reaction of encapsulated guest **3** inside of $\mathbf{1_2}$ as a function of time. ($D_2O$, 348K, [Host **1**] = 1 mM, [Guest **3**] = 0.5 mM, [NaOD] =10 mM, [NaClO4] = 10 mM).

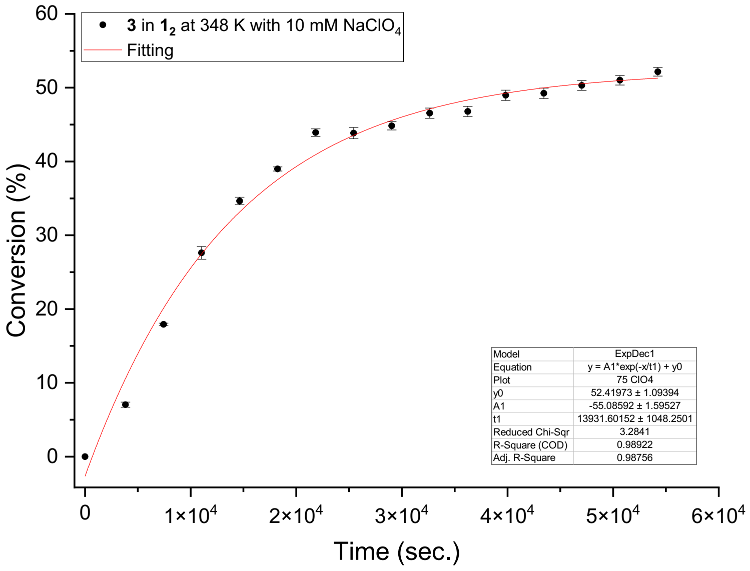


**Figure S84:** Reaction kinetics for guest **3** inside of host $\mathbf{1_2}$ in presence of 10 mM $NaClO_4$ at 348 K.

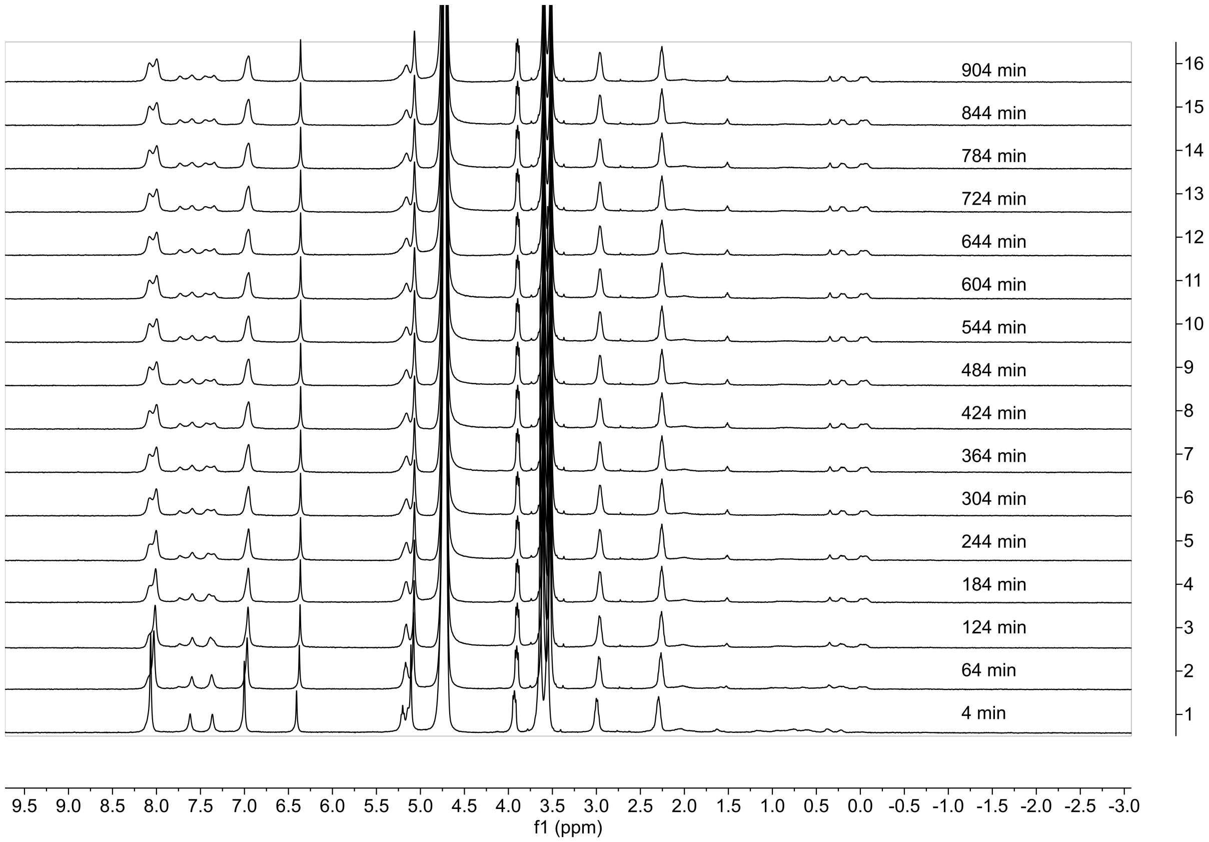


**Figure S85**: Stack of $^1H$ NMR spectra showing reaction of encapsulated guest **3** inside of $\mathbf{1_2}$ as a function of time. ($D_2O$, 351K, [Host **1**] = 1 mM, [Guest **3**] = 0.5 mM, [NaOD] =10 mM, [$NaClO_4$] = 10 mM).

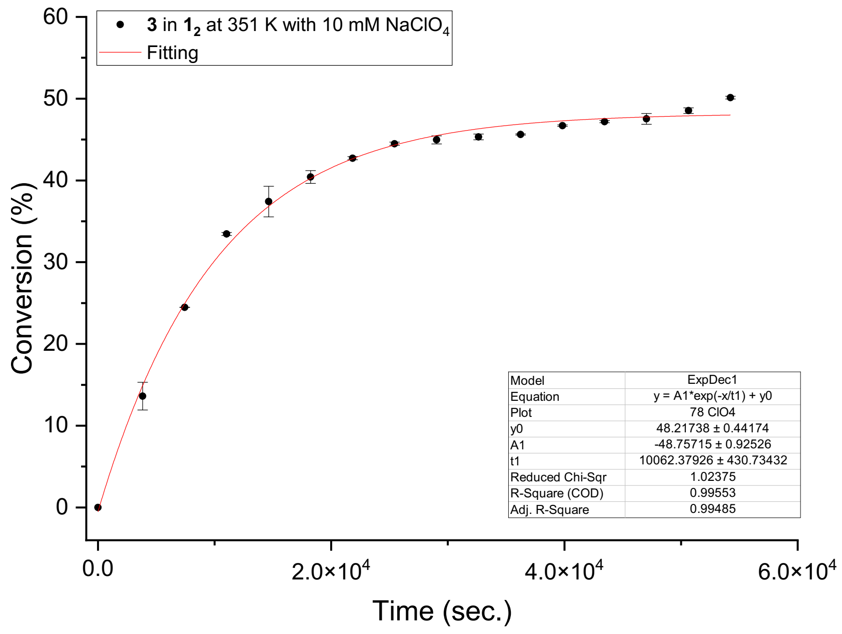


**Figure S86:** Reaction kinetics for guest **3** inside of host $\mathbf{1_2}$ in presence of 10 mM $NaClO_4$ at 351 K.

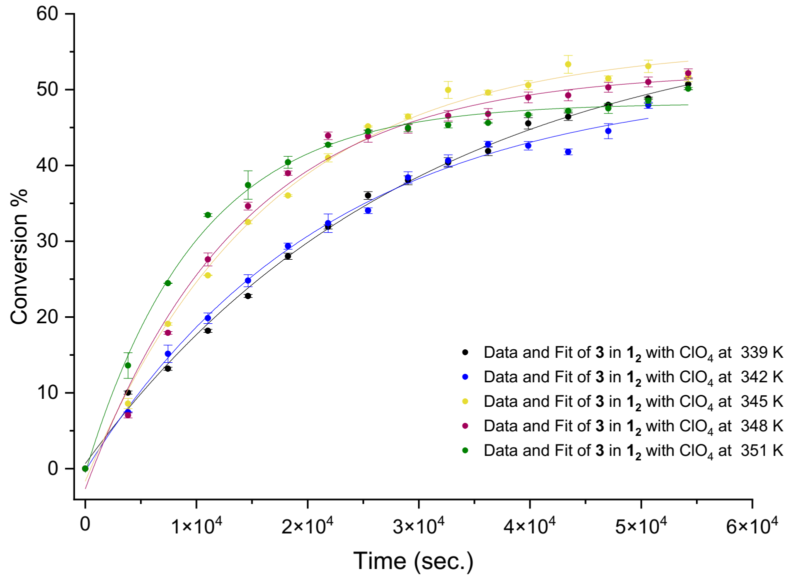


**Figure S87:** Fitting for the cyclization of guest **3** inside of $\mathbf{1_2}$ in presence of 10 mM $NaClO_4$ at five different temperatures.

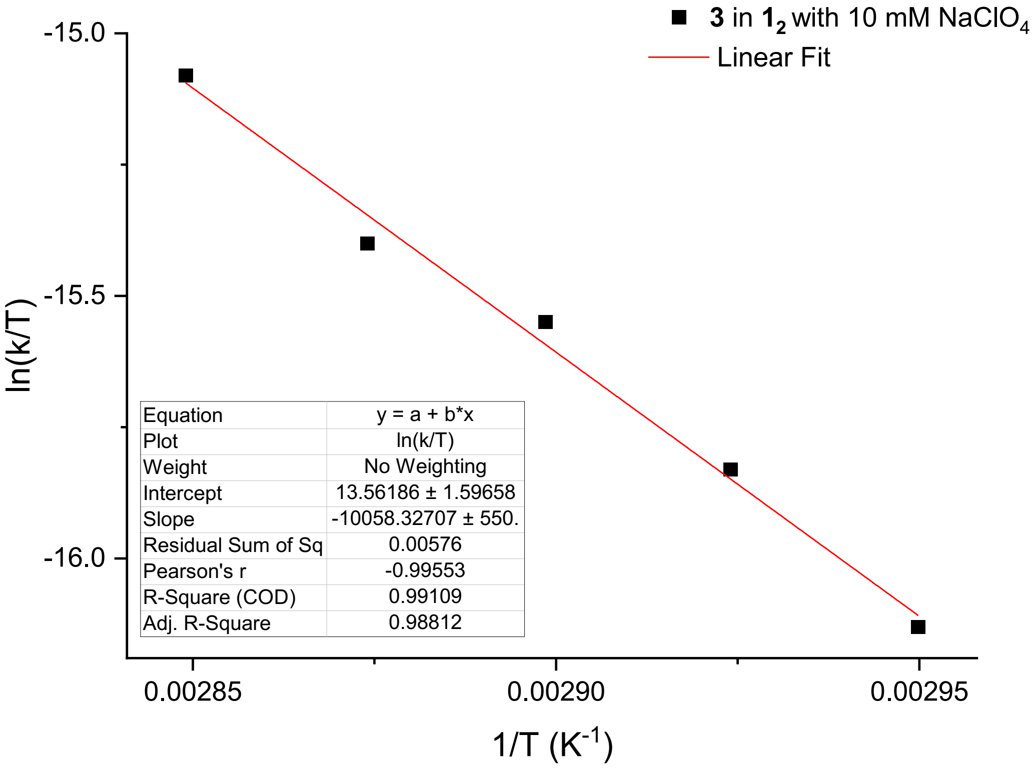


**Figure S88**: Eyring plot for the cyclization of guest **3** in **1**$_2$ in the presence of $NaClO_4$ at five different temperatures.

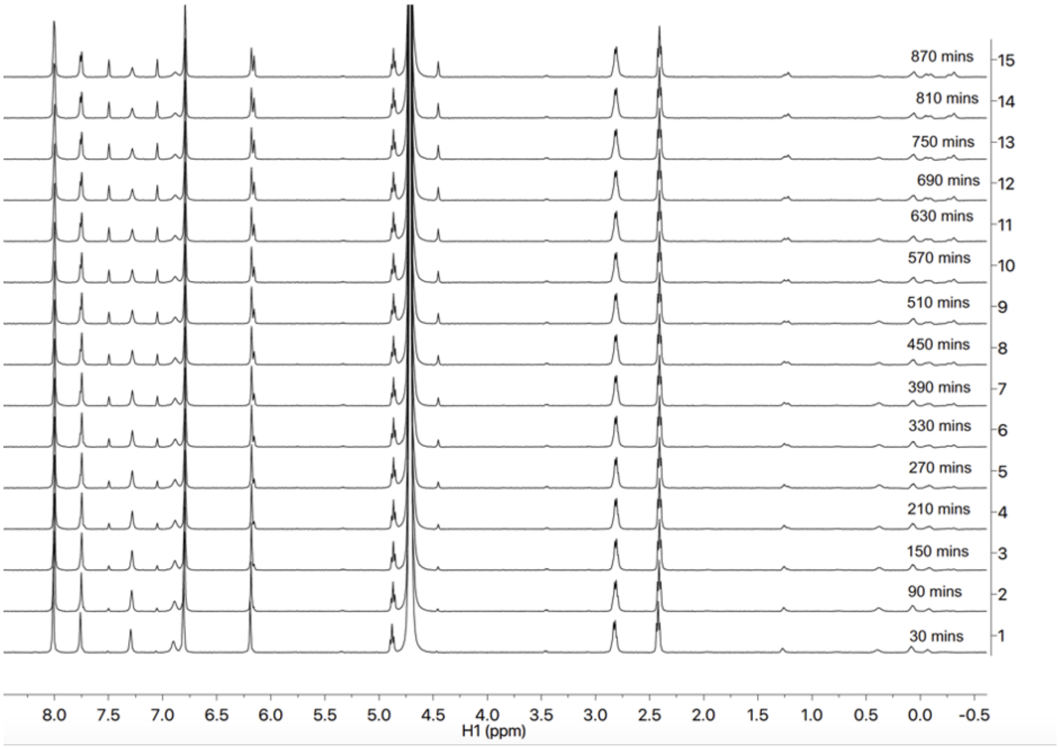


**Figure S89**: Stack of $^{1}$H NMR spectra showing reaction of encapsulated guest **3** inside of $\mathbf{2}_2$ as a function of time. ($D_2O$, 325 K, [Host **2**] = 1 mM, [Guest **3**] = 0.5 mM, [NaOD] =10 mM, [CsCl] = 10 mM).

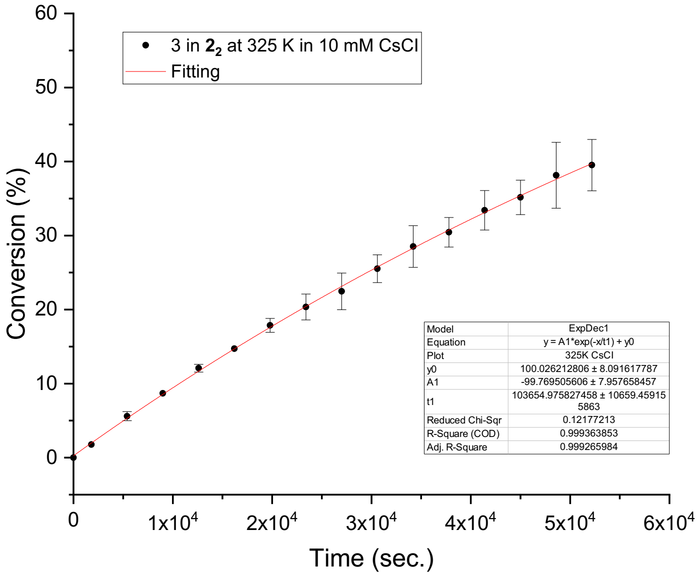


**Figure S90:** Reaction kinetics for guest **3** inside of host $\mathbf{2}_2$ in presence of 10 mM CsCl at 325 K

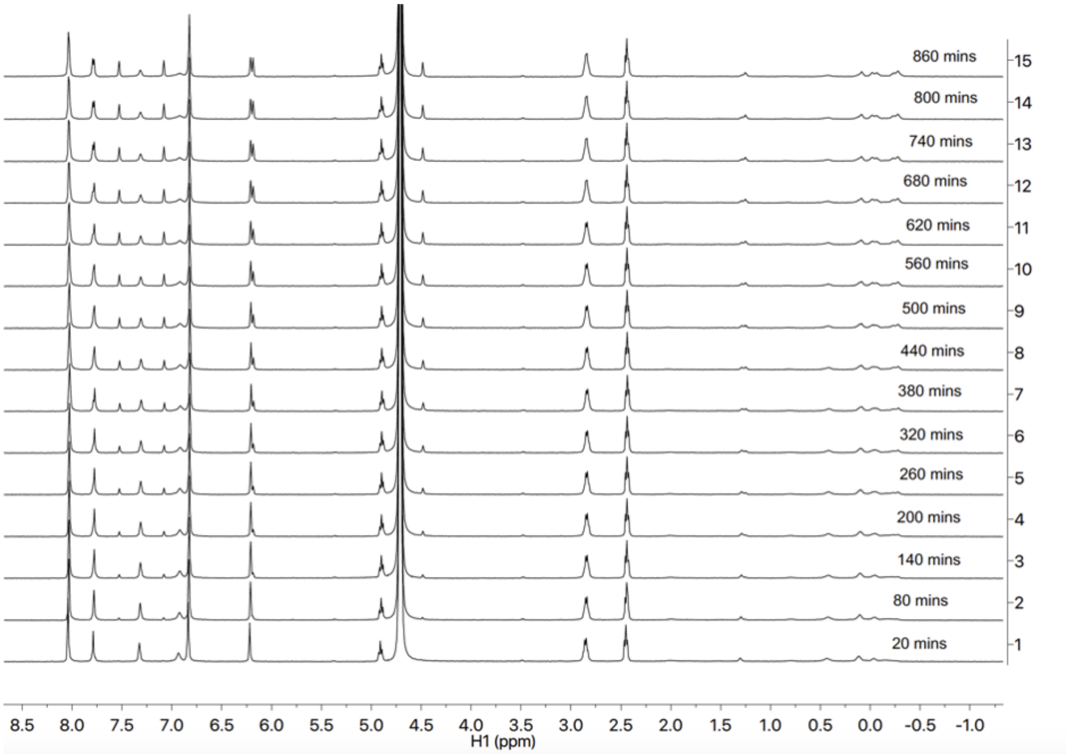


**Figure S91**: Stack of $^{1}$H NMR spectra showing reaction of encapsulated guest **3** inside of **2**$_2$ as a function of time. ($D_2O$, 328 K, [Host **2**] = 1 mM, [Guest **3**] = 0.5 mM, [NaOD] =10 mM, [CsCl] = 10 mM).

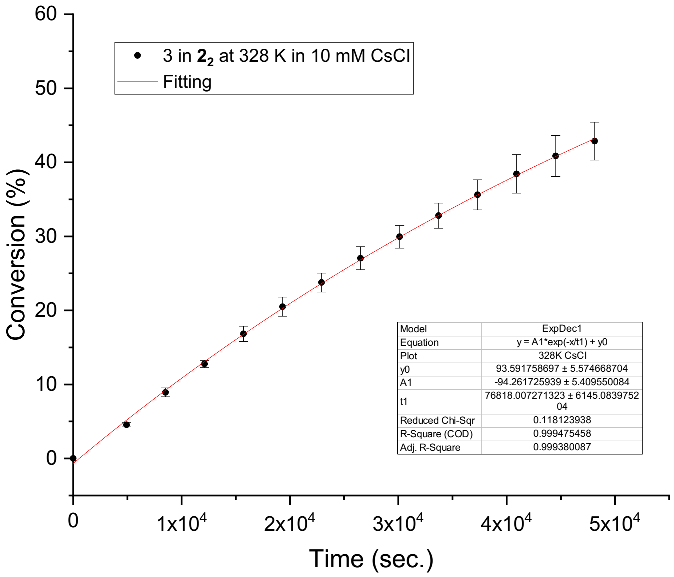


**Figure S92:** Reaction kinetics for guest **3** inside of host **2**$_2$ in presence of 10 mM CsCl at 328 K

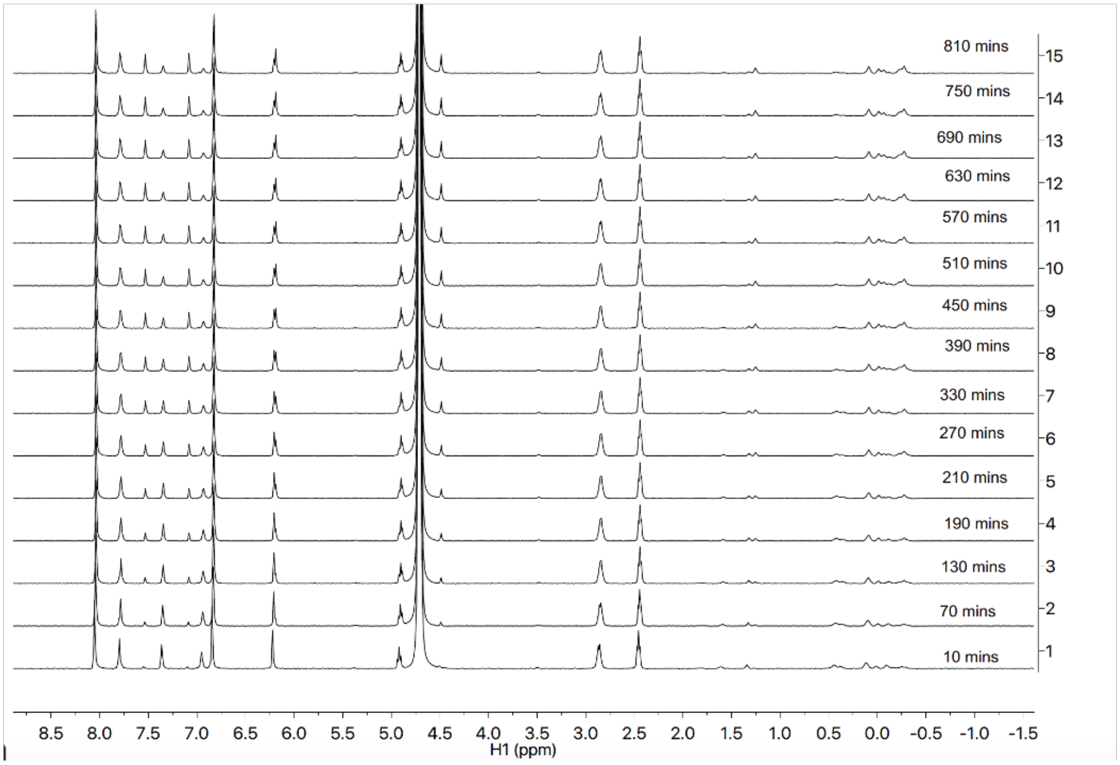


**Figure S93**: Stack of $^{1}$H NMR spectra showing reaction of encapsulated guest **3** inside of $\mathbf{2}_2$ as a function of time. ($D_2O$, 332 K, [Host **2**] = 1 mM, [Guest **3**] = 0.5 mM, [NaOD] =10 mM, [CsCl] = 10 mM).

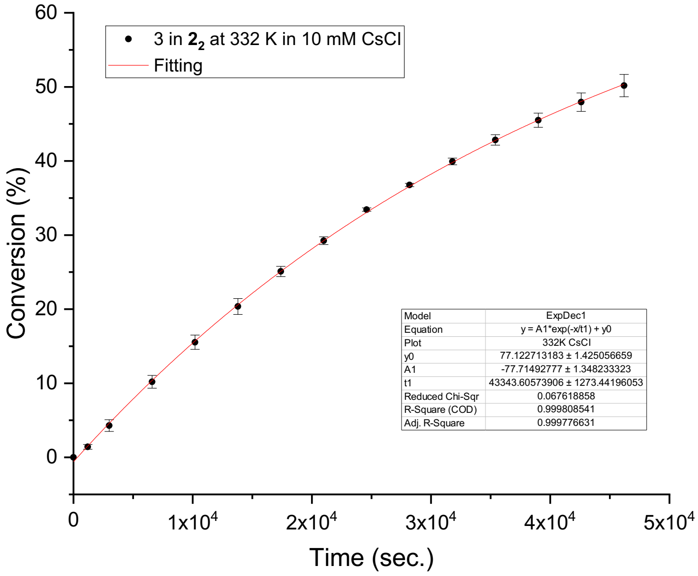


**Figure S94:** Reaction kinetics for guest **3** inside of host $\mathbf{2}_2$ in presence of 10 mM CsCl at 332 K

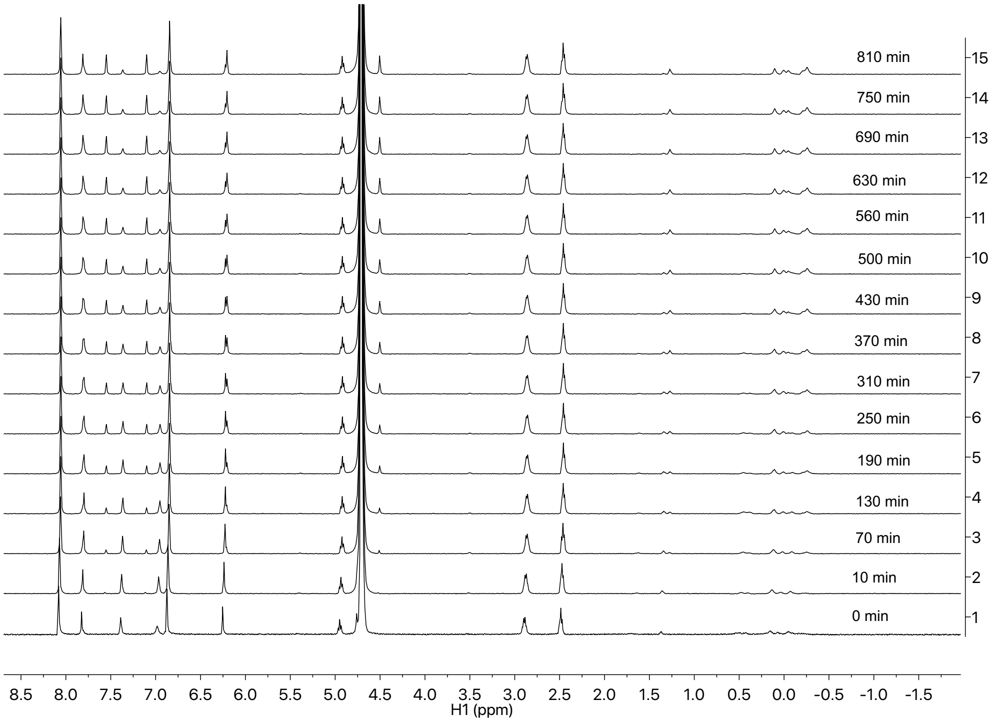


**Figure S95**: Stack of $^{1}$H NMR spectra showing reaction of encapsulated guest **3** inside of $\mathbf{2_2}$ as a function of time. ($D_2O$, 334 K, [Host **2**] = 1 mM, [Guest **3**] = 0.5 mM, [NaOD] =10 mM, [CsCl] = 10 mM).

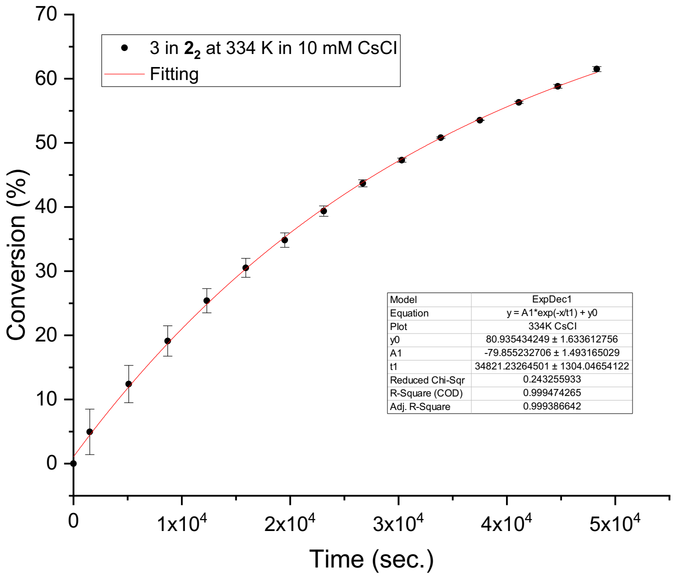


**Figure S96:** Reaction kinetics for guest **3** inside of host $\mathbf{2_2}$ in presence of 10 mM CsCl at 334 K

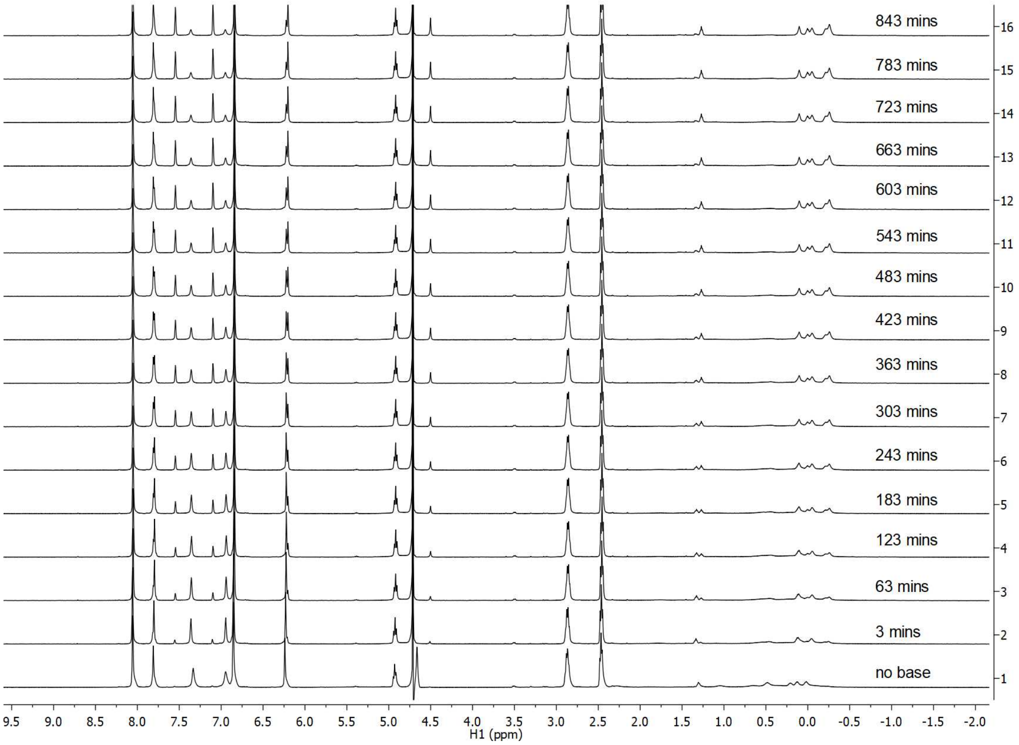


**Figure S97:** Stack of $^{1}$H NMR spectra showing reaction of encapsulated guest 3 inside of $2_2$ as a function of time. ($D_2O$, 336 K, [Host 2] = 1 mM, [Guest 3] = 0.5 mM, [NaOD] =10 mM, [CsCl] = 10 mM).

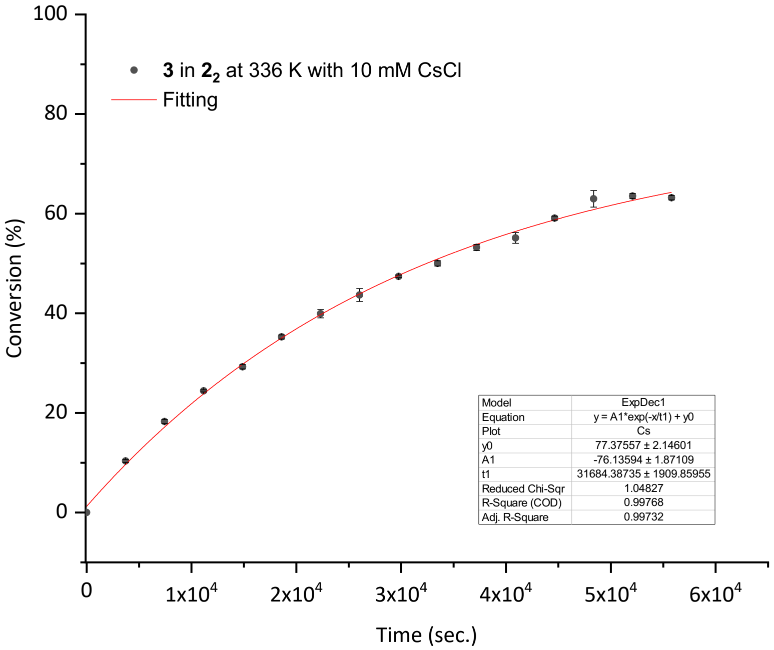


**Figure S98:** Reaction kinetics for guest **3** inside of host $\mathbf{2_2}$ in presence of 10 mM CsCl at 336 K

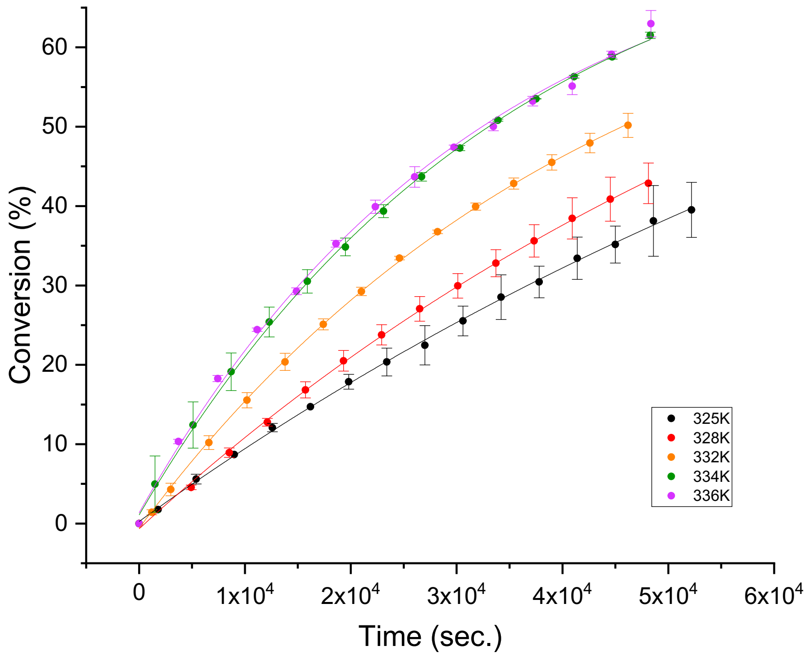


**Figure S99:** Fitting for the cyclization of guest **3** inside of $\mathbf{2}_2$ at five different temperatures with 10mM CsCl

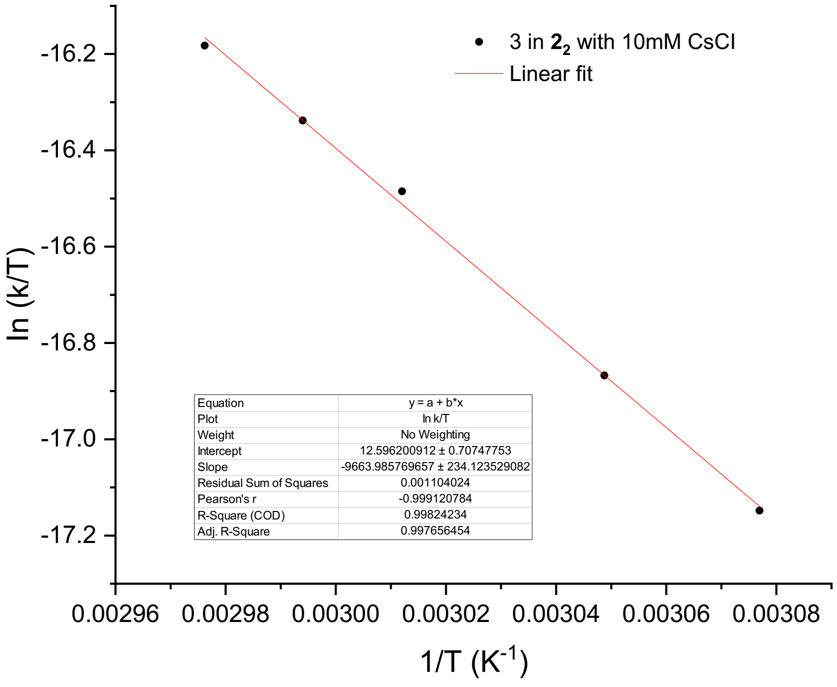


**Figure S100**: Eyring plot for the cyclization of guest **3** in $\mathbf{2}_2$ in presence of 10 mM CsCl at five different temperatures.

## 8) References


1. Gibb, C. L.; Gibb, B. C., Well-defined, organic nanoenvironments in water: the hydrophobic effect drives a capsular assembly. *J. Am. Chem. Soc.,* **2004,** *126* (37), 11408-9.
2. Liu, S.; Whisenhunt-Ioup, S. E.; Gibb, C. L.; Gibb, B. C., An improved synthesis of 'octa-acid' deep-cavity cavitand. *Supramol Chem* **2011,** *23* (6), 480-485.
3. Hillyer, M. B.; Gibb, C. L.; Sokkalingam, P.; Jordan, J. H.; Ioup, S. E.; Mague, J. T.; Gibb, B. C., Correction to "Synthesis of Water-Soluble Deep-Cavity Cavitands". *Org Lett* **2016,** *18* (24), 6523.
4. Greaves, J.; Munro, K. R.; Davidson, S. C.; Riviere, M.; Wojno, J.; Smith, T. K.; Tomkinson, N. C.; Chamberlain, L. H., Molecular basis of fatty acid selectivity in the zDHHC family of S-acyltransferases revealed by click chemistry. *Proc. Natl. Acad. Sci.,* **2017,** *114* (8), E1365-E1374.
5. Vigderman, L.; Manna, P.; Zubarev, E. R., Quantitative replacement of cetyl trimethylammonium bromide by cationic thiol ligands on the surface of gold nanorods and their extremely large uptake by cancer cells. *Angew. Chem. Int. Ed. Engl.,* **2012,** *51* (3), 636-41.
6. Halby, L.; Champion, C.; Senamaud-Beaufort, C.; Ajjan, S.; Drujon, T.; Rajavelu, A.; Ceccaldi, A.; Jurkowska, R.; Lequin, O.; Nelson, W. G.; Guy, A.; Jeltsch, A.; Guianvarc'h, D.; Ferroud, C.; Arimondo, P. B., Rapid synthesis of new DNMT inhibitors derivatives of procainamide. *ChemBioChem* **2012,** *13* (1), 157-65.
7. Jordan, J. H.; Gibb, C. L. D.; Wishard, A.; Pham, T.; Gibb, B. C., Ion-Hydrocarbon and/or Ion-Ion Interactions: Direct and Reverse Hofmeister Effects in a Synthetic Host. *J. Am. Chem. Soc.,* **2018,** *140* (11), 4092-4099.
